# The Cell Wall-Targeting Antibiotic Stimulon of *Enterococcus faecalis*

**DOI:** 10.1371/journal.pone.0064875

**Published:** 2013-06-03

**Authors:** Jacqueline Abranches, Pamella Tijerina, Alejandro Avilés-Reyes, Anthony O. Gaca, Jessica K. Kajfasz, José A. Lemos

**Affiliations:** 1 Center for Oral Biology, University of Rochester Medical Center, Rochester, New York, United States of America; 2 Department of Microbiology and Immunology, University of Rochester Medical Center, Rochester, New York, United States of America; University Medical Center Utrecht, The Netherlands

## Abstract

*Enterococcus faecalis* is an opportunistic nosocomial pathogen that is highly resistant to a variety of environmental insults, including an intrinsic tolerance to antimicrobials that target the cell wall (CW). With the goal of determining the CW-stress stimulon of *E. faecalis*, the global transcriptional profile of *E. faecalis* OG1RF exposed to ampicillin, bacitracin, cephalotin or vancomycin was obtained via microarrays. Exposure to the β-lactams ampicillin and cephalotin resulted in the fewest transcriptional changes with 50 and 192 genes differentially expressed 60 min after treatment, respectively. On the other hand, treatment with bacitracin or vancomycin for 60 min affected the expression of, respectively, 377 and 297 genes. Despite the differences in the total number of genes affected, all antibiotics induced a very similar gene expression pattern with an overrepresentation of genes encoding hypothetical proteins, followed by genes encoding proteins associated with cell envelope metabolism as well as transport and binding proteins. In particular, all drug treatments, most notably bacitracin and vancomycin, resulted in an apparent metabolic downshift based on the repression of genes involved in translation, energy metabolism, transport and binding. Only 19 genes were up-regulated by all conditions at both the 30 and 60 min time points. Among those 19 genes, 4 genes encoding hypothetical proteins (EF0026, EF0797, EF1533 and EF3245) were inactivated and the respective mutant strains characterized in relation to antibiotic tolerance and virulence in the *Galleria mellonella* model. The phenotypes obtained for two of these mutants, ΔEF1533 and ΔEF3245, support further characterization of these genes as potential candidates for the development of novel preventive or therapeutic approaches.

## Introduction

Enterococci are normal inhabitants of the gastrointestinal (GI) tract of humans and animals. While typically harmless to healthy individuals, two enterococcal species, *Enterococcus faecalis* and *E. faecium*, are the leading organisms involved in hospital-acquired infections such as catheter-associated urinary tract infections, endocarditis, and surgical and burn wound infections [1], [2], [3]. Notably, the risk of death for patients infected with multidrug resistant strains, such as vancomycin-resistant enterococci (VRE), is considerably higher than for those infected with antibiotic susceptible strains [4]. In addition, multidrug resistant enterococci pose an additional health care threat as these strains may function as reservoirs for the dissemination of antibiotic resistance determinants to other opportunistic pathogens [5].

Different from Gram-positive pathogens such as *Streptococcus pyogenes* and *Staphylococcus aureus*, enterococci do not appear to possess virulence factors such as pro-inflammatory toxins or immune modulators. The virulence of both *E. faecalis* and *E. faecium* is directly associated with the organism’s ability to survive hostile conditions, including an intrinsic resistance against host immune responses, antagonistic products produced by the competing microbial flora, and stresses imposed by eventual treatments with antimicrobials. When compared to closely related streptococcal and lactococcal species, enterococci are generally more resistant to a variety of stresses that damage the cell envelope, including nearly complete resistance to lysozyme and cephalosporins, and a relatively high tolerance to desiccation, detergents, and other inhibitors of peptidoglycan biosynthesis [1], [2], [3].

The intrinsic resistance to clinically useful β-lactam antibiotics (in particular cephalosporins and penicillinase-resistant penicillins) is likely to provide an additional selective advantage for both *E. faecalis* and *E. faecium*. In fact, treatment with broad-spectrum second and third generation cephalosporins has been long considered a risk factor for nosocomial *E. faecalis* bacteremia [6]. Although low-level resistance to β-lactams in enterococci has been associated with the increased synthesis of a low-affinity penicillin-binding protein (PBP) [7], many of the molecular events that result in the intrinsic resistance of *E. faecalis* and *E. faecium* to cell wall-stressing agents are poorly understood.

Genome-wide transcriptional profiling technologies, such as microarrays, have been applied to reveal new insights on how bacteria respond to antimicrobial challenges and to identify potential new targets for antimicrobial drug discovery [8], [9], [10], [11], [12]. In *E. faecalis,* microarrays have been used to investigate the transcriptional responses of this bacterium to growth in blood or urine [13], [14], to antibiotics that inhibit protein synthesis [12], [15], and to environmental stress such as amino acid starvation [16], iron excess [17] and to bile salts and SDS [18]. More recently, a functional genomic approach, termed Microarray-based Transposon Mapping (M-TraM), was used to identify *E. faecium* genes that contribute to ampicillin tolerance [19]. Despite the clinical relevance of cell wall-targeting agents in enterococcal infections, the genome-wide transcriptional profile of *E. faecalis* in response to this class of antibiotics has not been determined. In this study, microarray analyses of cells treated with four different cell wall-targeting antibiotics were conducted to unravel the scope of the cell wall (CW)-stress stimulon of *E. faecalis*. Based on the list of genes that belong to the CW-stress regulon, we selected six genes encoding conserved hypothetical proteins that appear to be unique to *Enterococcus* or restricted to Gram-positive bacteria for subsequent mutational analysis. Four of these six genes were successfully inactivated and their respective mutant strains characterized in relation to their tolerance to antibiotics and virulence in the *Galleria mellonella* model.

## Results

### Overview of the Microarray Analysis

To identify genes involved in the CW stress responses of *E. faecalis,* a whole genome transcriptional profile was performed using antibiotics that target different CW biosynthesis steps: the β-lactams ampicillin and cephalotin, which competitively inhibit the final transpeptidation step of the peptidoglycan synthesis; bacitracin, a polypeptide antibiotic that interferes with dephosphorylation of the lipid carrier responsible for moving the peptidoglycan precursors through the cytoplasmic membrane to the CW; and vancomycin, a glycopeptide that disrupts cross-linkage of peptidoglycan layers by preventing the incorporation of *N*-acetylmuramic acid- and *N*-acetylglucosamine-peptide subunits into the peptidoglycan matrix. The initial step in evaluating the responses of *E. faecalis* OG1RF to CW-inhibiting antibiotics was to determine the MIC for ampicillin, bacitracin, cephalotin (a first generation cephalosporin) and vancomycin. Then, for each antibiotic, a concentration corresponding to 1.25× the MIC was added to exponentially-grown cultures (OD_600_ 0.3), and aliquots were incubated aerobically at 37°C in the presence of the antibiotics for 30 and 60 min. The rationale for using 1.25× the MIC was to impose a stress without significantly affecting cell viability during the first few hours of antibiotic exposure. Under all tested conditions, OG1RF showed similar, and minimal, growth over the first 60 min incubation period indicating that all samples were at nearly identical growth phases (Fig. S1). These samples were then further analyzed by microarrays.

**Figure 1:**
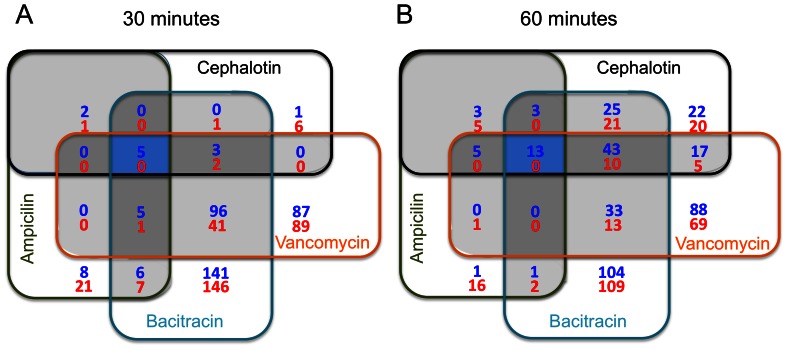
Venn diagrams depicting overlaps in gene expression among ampicilin, cephalotin, bacitracin and vancomycin after 30 and 60 min exposure. Blue shaded areas indicated overlaps among all four antibiotics. Dark grey areas depicts overlaps among three antibiotics and light grey between two antibiotics. Blue numbers represent upregulated genes whereas red numbers indicate downregulated genes.

Combining all test conditions in both 30 and 60 min time-points, 861 genes showed significant changes in total transcript amounts as compared to untreated control (*P* ≤ 0.01) but only a small subset of common genes appeared with altered expression under all conditions tested (Fig. 1). Additional Venn diagrams depicting the overlaps in gene expression for each antibiotic regimen between the two tested points are provided as part of the supplemental material (Fig. S2). The complete list of genes with altered expression under all four antibiotic treatments is provided in Table S1. Given that the *E. faecalis* V583 genome annotation [20] has been widely used in the literature, we adopted the V583 gene designation throughout the text and tables. However, differentially expressed genes present in the OG1RF genome but absent in V583 were presented with the original OG1RF designation [21]. The majority of genes found differentially expressed encoded for hypothetical proteins of unknown function (157 and 101 genes up- and down-regulated, respectively). Not surprisingly, cell envelope-related genes (65 up- and 39 down-regulated), and genes involved in transport and binding processes (45 up- and 86 down-regulated) were also well represented (Table S1).

To validate our microarray data, the expression of 9 selected genes identified with altered expression in the microarrays was verified by qRT-PCR. All genes showed the same expression trends as observed in the microarrays (Table S2).

### Exposure to Bacitracin and Vancomycin, but not to β-lactams, Induced Major Alterations in the Transcriptome of *E. faecalis* OG1RF

Among the four antibiotics tested, treatment with bacitracin resulted in the highest number of genes with altered expression (454 and 377 genes differently expressed 30 and 60 min post-treatment, respectively) (Table S1). In addition to the expected large number of cell envelope-related genes, expression of several genes encoding products that participate in central intermediary metabolism and proteins involved in transport and binding processes was also affected by bacitracin treatment. This large number of genes with altered expression could be related to the fact that bacitracin interference with CW biosynthesis occurs at early stages but also because bacitracin can inhibit isopentenyl pyrophosphate (IPP) biosynthesis. IPP is the central precursor of isoprenoids, via the mevalonate pathway [22]. The mevalonate pathway is the sole pathway for IPP biosynthesis in *E. faecalis* [23], and it is well known that isoprenoids are involved in a wide variety of vital biological processes such as electron transport and peptidoglycan biosynthesis [24]. Of note, four out of the six genes encoding enzymes of the mevalonate pathway were induced by bacitracin treatment (Table S1).

Exposure to vancomycin also resulted in major changes in the transcriptome of OG1RF, with 329 and 297 genes with altered expression at 30 and 60 min, respectively. In particular, a large number of genes (*n* = 40) encoding enzymes associated with cell envelope metabolism were up-regulated in vancomycin-treated cells (Table S1). Interestingly, genes from the mevalonate pathway were also induced by vancomycin, suggesting an association between IPP biosynthesis and CW homeostasis.

**Figure 2:**
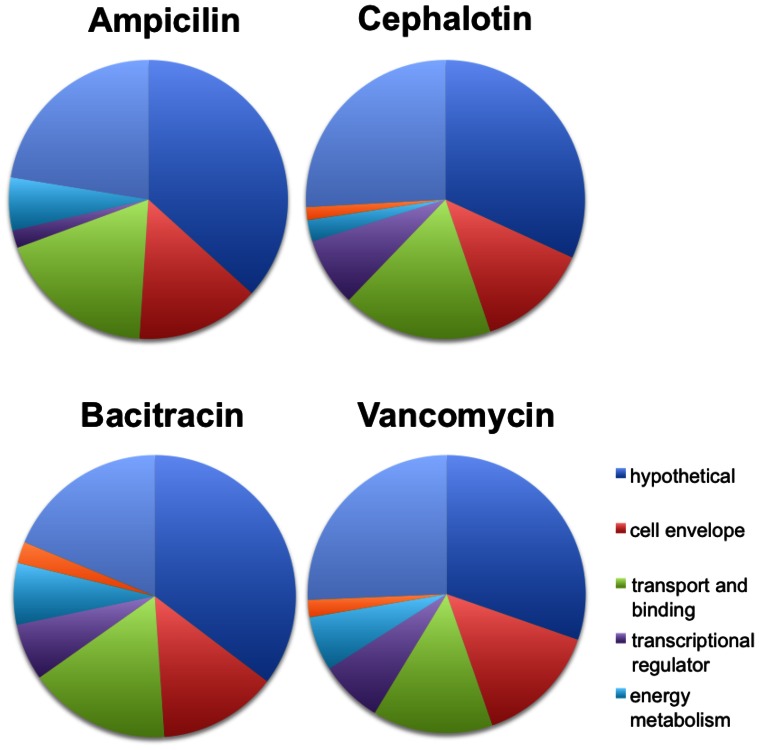
Pie chart of overrepresented functional categories with altered expression after 60 min of exposure to (A) ampicillin, (B) bacitracin, (C) cephalotin, and (D) vancomycin.

Unlike bacitracin and vancomycin, expression of a much smaller number of genes was affected by treatment with the β-lactams ampicillin and cephalotin. Treatment with cephalotin affected the expression of 21 and 192 genes at 30 and 60 min, respectively. Likewise, exposure to ampicillin for 30 and 60 min affected the expression of 56 and 50 genes, respectively. Even though the number of altered genes varied significantly among conditions, when the data were considered as groups of genes falling into particular functional categories, we observed a remarkably similar gene expression pattern for all four antimicrobials (Fig. 2), with an overrepresentation of genes encoding hypothetical proteins followed by genes encoding proteins associated with transport and binding and cell envelope metabolism.

Within the 861 genes shown in Table S1, the expression of 19 genes was found to be up-regulated by all conditions in at least one time-point (Table 1). Interestingly, the majority of these genes were predicted to encode conserved hypothetical proteins. We reasoned that genes that were commonly induced by all four CW-active antibiotics might be key players in the CW stress response of *E. faecalis*. From this list of 19 genes, we selected 6 genes coding for hypothetical proteins that are unique to enterococci or closely Gram-positive bacteria (EF0026, EF0708, EF0797, EF1258, EF1533 and EF3245) for subsequent mutational analysis.

**Table 1:** Genes upregulated by Ampicilin (Amp), Bacitracin (Bac), Cep (Cephalotin) and Vancomycin (Van) 30 or 60 min after exposure.

Gene ID	Definition	Amp 30′	Amp 60′	Bac 30′	Bac 60′	Cep 30′	Cep 60	Van 30′	Van 60′
EF0026	hypothetical protein	2.68	9.07	19.06	7.64	4.20	12.17	6.17	11.9
EF0708	hypothetical protein	3.66	2.99	15.86	5.97		9.01	9.2	16.39
EF0797	hypothetical protein	4.43	8.12	10.19	12.95	4.45	11.30	5.52	10.86
EF0802	hypothetical protein		6.26	36.55	16.21		29.28	134.36	232.04
EF1224	transcriptional regulator		2.96	3.51	16.54		3.01		12.5
EF1258	hypothetical protein		3.8	36.83	11.18		8.47	23.69	21.47
EF1304	Mg-importing ATPase	2.52		5.21	15.98		6.66	24.62	80.97
EF1533	hypothetical protein	6.87	11.7	62.15	38.18	7.46	38.96	13.64	21.61
EF1587	nudix family phosphohydrolase	2.82	2.61	22.39	11.86		6.7	15.45	19.3
EF1753	hypothetical protein	4.40	3.95	25.31	18.40	3.74	10.2	11.63	18.16
EF1814	EmbR/QcaA drug resistance transporter		6.01	8.05	7.86		14.35	14.55	
EF2784	hypothetical protein	2.66		17.35	12.3		4.95	4.93	
EF2892	hypothetical protein		3.8	7.93			6.71	19.78	29.85
EF2896	hypothetical protein		6.23	35.23	30.21		23.38	112.37	97.22
EF2913	membrane protein		2.24	8.4	4.16		3.71		2.95
EF3057	hypothetical protein		6.61	5.34			9.22	4.8	7.35
EF3239	hypothetical protein	3.48	7.44	8.44	5.09		13.76	3.4	
EF3245	lytR-cpsA-psr and PAP-2 superfamilies	4.28	8.76	43.91	30.51	5.51	34.13	81.30	112.6
OG0126	ABC transporter superfamily		3.71		11.12		5.12	20.06	19.1

Fold change results from microarrays comparison of antibiotic-treated samples with untreated samples. Blank cells indicate that there were no significant differences between treated and untreated cells. Genes in bold were selected for subsequent mutational analysis.

### Antimicrobial Susceptibility of Targeted Mutants

**Figure 3:**
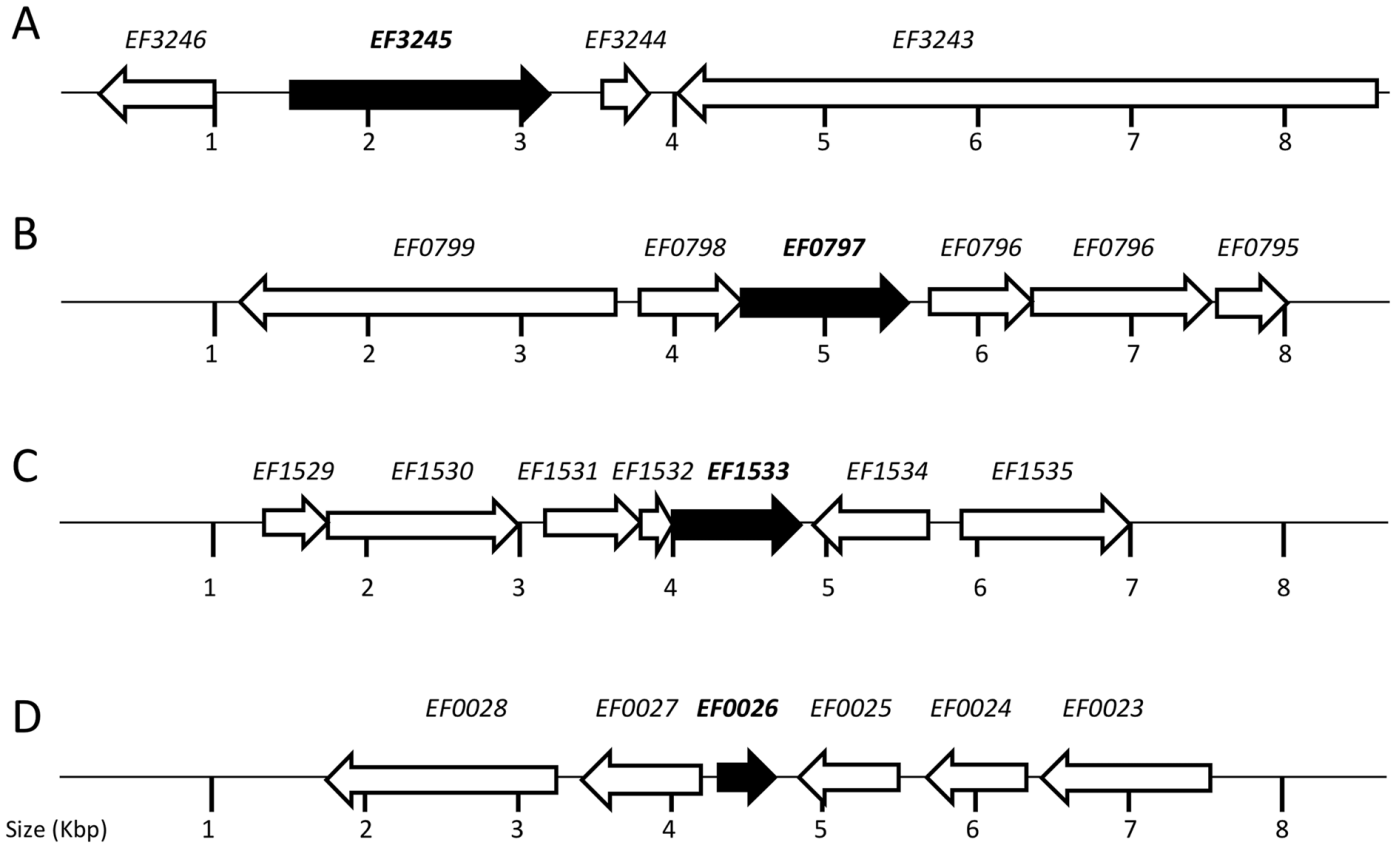
Schematic representation of the EF0026, EF0797, EF1533 and EF3245 loci and flanking regions. (A) EF3245 is in an apparent monocistronic operon and is flanked by a chitin-binding protein (EF3246), a small hypothetical protein (EF3243) and a large hypothetical protein with a CW-binding domain (EF3244). (B) EF0797 is apparently co-transcribed with another hypothetical protein (EF0798) and both genes flanked by a putative protein belonging to the Type 2 phosphatidic acid phosphatase family (EF0796) and a putative autolysin (EF0799). (C) EF1533 appears to be the last gene of a three-gene operon with EF1532 coding for a hypothetical protein and EF1531 coding for a putative TetR transcriptional regulator. EF1531–1533 gene cluster is flanked by a PTS transporter subunit (EF1530) and a peptidyl-prolyl cis-trans isomerase (EF1534). (D) EF0026 is flanked by a putative membrane protein (EF0025) and a putative transcriptional regulator (EF0027) whereas EF0024 and EF0023 encode a hypothetical protein and a mannose-fructose-sorbose PTS porter, respectively.

Despite multiple attempts, we were not able to isolate mutants lacking the EF0708 and EF1258 genes. Both genes are predicted to encode small conserved hypothetical proteins (63 and 68 amino acids, respectively). Interestingly, EF0708 is only found in selected Gram-positive bacteria (e.g., Enterococci, Bacilli, Lactobacilli and Geobacilli) whereas EF1258 appears to be unique to *E. faecalis* strains. Markerless deletions of the remaining genes were readily obtained, and the deletions were confirmed by PCR sequencing. BLAST search analysis indicated that the putative hypothetical proteins encoded by the EF0026, EF0797 and EF1533 genes are relatively conserved among Gram-positive bacteria. Interestingly, EF3245 is unique to *E. faecalis* mostly because it appears to encode a dual function protein possessing two conserved domains, PAP2 and LytR-CpsA-Psr. PAP2 is a superfamily of phosphatases and haloperoxidases that may act as a membrane-associated lipid phosphatase whereas LytR-CpsA-Psr is a superfamily of cell envelope-related transcriptional attenuators. The genetic organization of the four mutated genes is shown in Figure 3.

Under standard growth conditions, e.g. brain heart infusion (BHI) broth at 37°C under aerobic conditions, all mutants grew as well as the parent strain OG1RF (Table 2). With the exception of ΔEF1533, which formed longer cell chains when grown in broth, all other mutants had no obvious morphological cell differences (data not shown). To evaluate the susceptibility of the ΔEF0026, ΔEF0797, ΔEF1533 and ΔEF3245 strains to antibiotics that inhibit CW biosynthesis, the minimum inhibitory concentration (MIC) and growth under sub-MIC of each strain to ampicillin, bacitracin, cephalotin and vancomycin was assessed. When compared to the parent strain, all mutants displayed the same MIC to ampicillin and cephalotin (16 µg ml^−1^ and 64 µg ml^−1^, respectively) and, with the exception of ΔEF1533 that showed a slightly slower growth in cephalotin, all strains grew equally well in sub-inhibitory concentrations of these two β-lactams (Table 2). Strains ΔEF1533 and ΔEF3245 showed, respectively, two- and four-fold lower MIC for bacitracin when compared to OG1RF (MIC = 64 µg ml^−1^). On the other hand, when compared to the MIC of OG1RF in vancomycin (8 µg ml^−1^), ΔEF0797and ΔEF1533 had two-fold lower MIC values whereas ΔEF0026 showed a two-fold higher MIC. In agreement with the MIC values obtained, both ΔEF1533 and ΔEF3245 strains grew slower in sub-inhibitory concentrations (16, 32 and 64 µg ml^−1^) of bacitracin (Table 2). Strains ΔEF0026 and ΔEF0797 grew as well as the parent strain in the presence of different concentrations of bacitracin (Table 2). Also in agreement with the MIC values, ΔEF1533 grew significantly slower in sub-MIC concentrations (1, 2 and 4 µg ml^−1^) of vancomycin (Table 2). Strains ΔEF0026, ΔEF0797 and ΔEF3245 grew as well as the parent strain in the presence of different concentrations of vancomycin (Table 2). Unexpectedly, all strains showed shorter doubling times in medium containing 1 µg ml^−1^ vancomycin than in medium without added antibiotics. While it is clear that this concentration of vancomycin does not cause a negative impact cell growth, the basis for the faster growth rates remains unknown.

**Table 2:** Doubling times of OG1RF, EF0026, EF797, EF1533 and EF3245 in various antibiotics concentrations.

	OG1RF (min ± SD)	EF0026 (min ± SD)	EF0797 (min ± SD)	EF1533 (min ± SD)	EF3245 (min ± SD)
BHI only	62.3±0.8	61.8±0.7	63.5±4.5	67.7±3.4	60±0.6
Vancomycin (1 µg ml^−1^)	57.5±1.3	56.7±1	59.6±1.4	65.9±2.4*	55.9±0.4
Vancomycin (2 µg ml^−1^)	60.7±1.7	57.5±0.9	64.4±2.2	68.1±3.27*	58±0.26
Vancomycin (4 µg ml^−1^)	73.4±1	73.8±1.6	72±1.4	88.5±1.4*	81.8±7.9
Bacitracin (8 µg ml^−1^)	71.3±0.8	68.7±1.4	72.9±0.5	85.8±0.7*	73.5±2.5
Bacitracin (16 µg ml^−1^)	73.6±6.1	70.6±1.8	72.2±2.3	98.4±2*	96.1±1.7*
Bacitracin (32 µg ml^−1^)	76.4±0.6	78.8.0±3	75.6±2	128.8±2.4*	132. ±1*
Cephalotin (15 µg ml^−1^)	65.6±0.92	63.9±0.72	63.5±0.2	71.3±0.27*	61.4±0.28
Ampicilin (1 µg ml^−1^)	72.9±2.4	70.7±3.2	70.7±1.2	72.6±1.4	70. 8±1.7

Data presented represents the average in minutes and standard deviation of at least three independent experiments. Numbers followed by * represent a statistically significant difference (*p*≤0.05) compared to the parental strain OG1RF under the same growth condition using Student’s *t* test.

**Figure 4:**
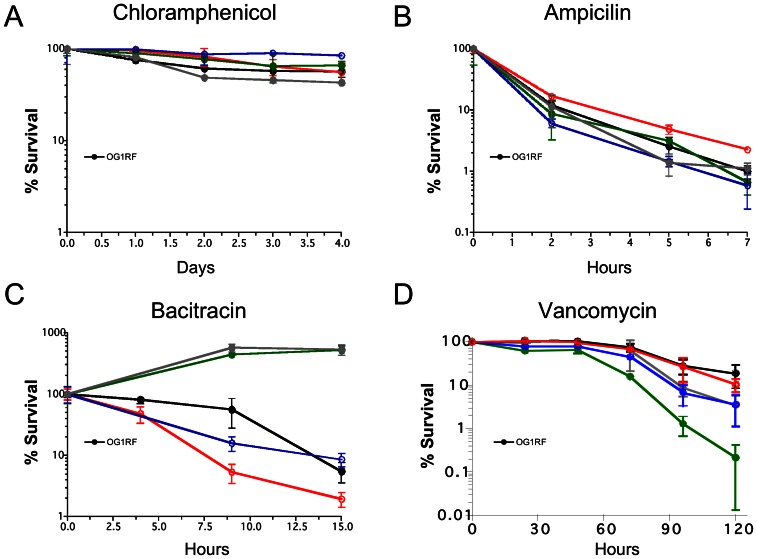
Cell death kinetics of OG1RF, ΔEF3245, ΔEF0797, ΔEF1533 and ΔEF0026 in (A) chloramphenicol (160 µg ml^−1^), (B) ampicillin (80 µg ml^−1^), (C) bacitracin (320 µg ml^−1^), and (D) vancomycin (80 µg ml^−1^). Experiments were performed in triplicates with averages and standard deviations calculated for each time-point. Student’s *t* test was performed to verify significance.

**Figure 5:**
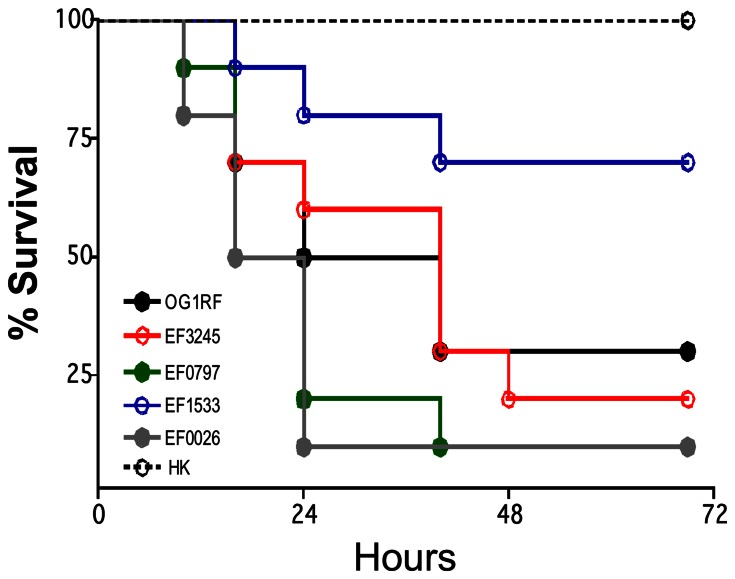
*Galleria mellonella* survival after injection with 5×10^5^ CFU of OG1RF, ΔEF3245, ΔEF0797, ΔEF1533, ΔEF0026 and heat-killed (HK) OG1RF. Using the log-rank test to compare against the wild-type OG1RF strain, ΔEF1533 showed attenuated virulence whereas ΔEF0026 and ΔEF0797 were more virulent (*p*<0.05). Data presented is a representative of at least three independent experiments.

Next, we carried out time-kill assays in the presence of ampicillin, bacitracin or vancomycin such that all strains were tested under concentrations that correspond to 5 or 10X the MIC for the selected antibiotic. The bacteriostatic antibiotic chloramphenicol was used as a control to monitor cell viability over time. Incubation in the presence of chloramphenicol (10X MIC) did not result in statistically significant loss of cell viability when compared to untreated cells and all strains survived equally well over a 4-day period (Fig. 4A). Essentially, no differences between strains were observed in cells treated with ampicillin (Fig. 4B), albeit ΔEF3245 showed a modest, but not statistically significant, increased survival. Consistent with lower MIC and slower growth rates in bacitracin, ΔEF3245 was killed significantly more rapidly by bacitracin (320 µg ml^−1^, which corresponds to 5X the MIC for the parent strain but 20X for ΔEF3245 (Fig. 5C). On the other hand, despite showing a lower MIC and slower sub-MIC growth in the presence bacitracin, the time-kill kinetics of ΔEF1533 was not significantly different than the parent OG1RF strain when cells were exposed to 320 µg ml^−1^ (10X MIC) of bacitracin (Fig. 4C). Of note, ΔEF0026 and ΔEF0797 were completely resistant to bacitracin killing over the course of the experiment (Fig. 4C). Interestingly, lower concentrations of bacitracin corresponding to 5X the MIC for bacitracin for EF1533 strain (160 µg ml^−1^) and EF3245 (80 µg ml^−1^) did not affect cell viability of all tested strains over the course of the experiment (Fig. S3). Despite a two-fold higher MIC for vancomycin, ΔEF0026 survived as well as the parent strain in vancomycin time-kill assays, even when higher concentrations of vancomycin were used (Fig. 4C and Fig. S3). Consistent with the lower MIC in vancomycin, ΔEF0797 and ΔEF1533 were killed more rapidly by concentrations that correspond to 20X (80 µg ml^−1^, ΔEF0797 only) or 10X (40 µg ml^−1^, both ΔEF0797 and ΔEF1533) the MIC for vancomycin (Fig. 4D and Fig. S3). The summary of the antibiotic-resistant profile of each mutant strain is shown in Table 3.

**Table 3:** Summary of the antibiotic-resistance profile of ΔEF0026, ΔEF0797, ΔEF1533 and ΔEF3245 in relation to that of OG1RF (wild-type strain).

Strain	Ampicillin			Bacitracin			Cephalotin			Vancomycin		
	Growth	MIC	Killing	Growth	MIC	Killing	Growth	MIC	Killing	Growth	MIC	Killing
ΔEF0026	ND	ND	ND	ND	ND	↑	ND	ND	NT	ND	↑	ND
ΔEF0797	ND	ND	ND	ND	ND	↑	ND	ND	NT	ND	↓	↓
ΔEF1533	ND	ND	ND	↓	↓	ND	↓	ND	NT	↓	↓	↓
ΔEF3245	ND	ND	ND	↓	↓	↓	ND	ND	NT	ND	ND	ND

ND, no differences in comparison to OG1RF.

NT, not tested.

↓, sensitive in comparison to OG1RF.

↑, resistant in comparison to OG1RF.

### Virulence of ΔEF0026 and ΔEF0797 and ΔEF1533 was Altered in the *G. mellonella* Model

Larva of the Lepidoptera *Galleria mellonella* has been shown to serve as a surrogate model to study bacterial virulence for a number of pathogens [25], including *E. faecalis* [16], [26], [27], [28]. Here, we tested the virulence potential of the ΔEF0026, ΔEF0797, ΔEF1533 and ΔEF3245 strains injected into *G. mellonella* (Fig. 5). While the ΔEF3245 strain was able to kill *G. mellonella* as efficiently as the parent OG1RF strain, virulence of ΔEF1533 was attenuated (*p*<0.05). Conversely, strains ΔEF0026 and ΔEF0797 were significantly more virulent than OG1RF (*p*< 0.05).

## Discussion

In this study, we used microarrays to obtain a snapshot of the transcriptional responses of *E. faecalis* to four different antibiotics that target CW biosynthesis, namely ampicillin, bacitracin, cephalotin and vancomycin. Treatment with all four drugs, most notably bacitracin and vancomycin, resulted in an apparent metabolic downshift based on the repression of a number of genes involved in translation, energy metabolism, transport and binding. This may be an indication that cells are saving or redirecting energy resources to maintain CW integrity and survive the physiological stress imposed by the antibiotics. In fact, genes involved in CW metabolism were highly affected, and overwhelmingly up-regulated during antibiotic treatment. For example, several genes coding for penicillin binding proteins (PBPs), responsible for CW cross-linking, and genes from the *dlt* operon, responsible for D-alanylation of lipoteichoic acids (LTA), were highly induced by bacitracin, cephalotin and vancomycin. Notably, esterification of LTA with D-alanine has been directly linked to vancomycin tolerance in both *S. aureus* and *E. faecium* [29], [30].

Some attention has been devoted to investigate the possible association of oxidative stress with antibiotic-mediated cell death [31]. More specifically, these authors proposed that bactericidal drugs, irrespective of the cellular target, kill bacteria by triggering endogenous production of hydroxyl radical. In a separate study, a panel of strains deficient in the production of known antioxidant enzymes was used to suggest that accumulation of superoxide anion, but not peroxide, was responsible for the bactericidal effects of penicillin and vancomycin in *E. faecalis* [32]. However, two recent investigations using the Gram-negative paradigm *E. coli* challenged the concept of the common ROS-mediated antibiotic killing pathway. Specifically, Liu and Imlay showed that different antibiotics targeting the cell wall, DNA replication or protein synthesis had little impact on respiration rates, did not promote H_2_O_2_ formation, caused no damage to ROS-sensitive metallo-enzymes and did not activate the OxyR regulon [33]. In the second study, Keren and co-workers observed no obvious differences in antibiotic survival of *E. coli* cells under aerobic or anaerobic conditions [34]. In our study, we found that the expression of a number of oxidative stress genes, such as *ahpC* (alkyl hydroperoxide), *katA* (heme-dependent catalase), *msrA* (methionine sulfoxide reductase A), *nox* (H_2_O-forming NADH oxidase), *npr* (NADH peroxidase) and *ohr* (organic hydroperoxide resistance), was induced during bacitracin exposure. However, a few other typical antioxidant genes, such as *sodA* (superoxide dismutase) and *tpx* (thiol peroxidase), showed the opposite transcriptional pattern (e.g. repressed by bacitracin). Thus, the linkage of oxidative stress gene expression with CW stress in *E. faecalis* is also not clear. Of note, previous transcriptional profiling studies of the envelope stress responses of *Bacillus subtilis*, *S. aureus* and *Streptococcus pneumoniae* did not establish a firm correlation between the induction of oxidative stress genes and CW stress responses [35], [36], [37]. Given the recent controversy surrounding the common ROS-mediated antibiotic killing pathway model [31], [33], [34], further studies, perhaps by measuring the production of reactive oxygen species (ROS) as well as the activity of antioxidant enzymes during antibiotic treatment, are necessary to conclusively determine the relevance of oxidative stress damage in antibiotic-mediated killing of *E. faecalis* as well as other closely-related Gram-positive bacteria.

The expression of over 50 transcriptional regulators was affected by the different test conditions. Among those were 19 genes coding for two-component signal transduction systems (TCSTS) including members of the LiaRS, LytRS and VanRS families. The *vanS* and *vanR* TCSTS genes in OG1RF encode proteins with high homology with the *van*_G_ locus responsible for vancomycin resistance in certain strains of *E. faecalis* [38]. Multiple evidences derived from different Gram-positive bacteria indicate that the LiaRS and LytRS TCSTS are involved in regulation of CW metabolism. In *Streptococcus gordonii*, LiaSR was shown to control the expression of the *dlt* operon in response to cell envelope perturbations and extracellular pH [39]. In *Listeria monocytogenes*, transcription of the *liaR* and *liaS* genes was induced by bacitracin and vancomycin [40], and a *liaS* mutant strain exhibited increased sensitivity to cephalosporins [41]. Finally, treatment with vancomycin induced the expression of the LiaRS-encoding genes in *S. pneumoniae* [42]. In *B. subtilis* and *S. aureus*, *lytR*-like genes were induced by CW-active antibiotics [35], [36]. The LytRS TCSTS of *S. aureus* has been shown to play a role in the control of autolysis [43]. Notably, in a vancomycin intermediate *S. aureus* (VISA) strain, *lytRS* expression was repressed in comparison to the parental strain upon exposure to vancomycin, suggesting a role for LytRS in sensing envelope damage and controlling CW turn over and autolysis [8]. In addition to the putative LytRS TCSTS, transcription of 3 additional genes with putative LytR domains (EF0465, EF1212 and EF3245) was induced by at least 2 of the 4 tested antibiotics.

In an effort to discover new genes responsible for the intrinsic tolerance of *E. faecalis* to CW-damaging agents, we selected 6 genes for mutational analysis based on three major criteria: (i) the genes were induced by all 4 antibiotics tested, (ii) the genes appeared to be uniquely found in enterococci or were restricted to Gram-positive bacteria, and (iii) the genes encode hypothetical proteins of unknown function or putative regulatory proteins based on the presence of a DNA-binding domain. Initially, we attempted to generate deletions in EF0026, EF0708, EF0797, EF1533, EF1753 and EF3245. However, we were unable to confirm mutations of EF0708 and EF1258, suggesting that these genes may perform an essential role for cell viability, or that we did not use appropriate conditions for mutant isolation. Further studies, such as the construction of conditional mutants, are necessary to confirm the essentiality of these genes. If proven to be essential, both EF0708 and EF1258 may be viewed as desirable targets for the development of novel therapeutic and prevention approaches.

The ΔEF0026 strain showed a two-fold higher MIC for vancomycin and enhanced survival during bacitracin killing (see Table 3). Given that EF0026 was induced under all tested conditions, these findings were unexpected. The ΔEF0797 strain also showed enhanced survival during bacitracin killing but had a lower MIC and increased susceptibility to vancomycin. In addition, both ΔEF0026 and ΔEF0797 were more virulent in *G. mellonella*, an obviously undesirable trait in the drug discovery process. Based on these results, the EF0026 and EF0797 genes may not be desirable candidates for the development of new antibacterial therapies. In a recent study, a library of 177 insertion mutations in genes encoding putative surface or stress-response factors in *E. faecalis* V583 was screened for several phenotypes including resistance to antibiotics and virulence in *G. mellonella* [27]. Two of the four genes investigated in our study, EF0797 and EF3245, were also characterized in the aforementioned large-scale study. In contrast with our results, the authors did not observe any important phenotypic differences in their EF0797 and EF3245 mutant strains [27]. However, both studies have relevant differences ranging from the different type wild-type strain used in the study (OG1RF vs. V583), mutant construction and general growth conditions.

EF1533 is a gene coding for a conserved hypothetical protein with 125 amino acid residues that is ubiquitously found in *E. faecalis* strains. Homologues of EF1533 can also be found in *E. faecium* and other enterococcal species as well as in lactobacilli, staphylococci and bacilli (≈50% similarity). EF1533 appears to be the last gene of a three-gene operon with the first gene (EF1531) coding for a putative TetR transcriptional regulator and the second gene (EF1532) coding for a hypothetical protein (see Fig. 3). The EF1533 mutant showed increased susceptibility to vancomycin and bacitracin and grew more slowly under sub-MIC of cephalotin (see Table 3). Furthermore, virulence of ΔEF1533 was significantly attenuated in *G. mellonella*. Collectively, these results indicate that EF1533 may play a role in CW homeostasis and support further characterization of EF1533 as a potential new drug target.

The ΔEF3245 strain showed attenuated virulence in the *G. mellonella* model and displayed increased sensitivity to bacitracin. Interestingly, the 538 amino acid residues long EF3245 is unique to *E. faecalis* and possess two distinct domains: an N-terminal type-2 phosphatidic acid phosphatase (PAP2) superfamily domain and a C-terminal lytR-cpsA-psr superfamily domain of cell envelope-associated transcriptional attenuators. Members of the PAP2 superfamily have been proposed to serve as integral membrane proteins that catalyze the dephosphorylation of phospholipids [44], [45], [46]. In *B. subtillis*, overexpression of a PAP2 protein, named BcrC, is directly involved in bacitracin tolerance [47]. Thus, it is conceivable that EF3245 encodes a dual function protein that contributes to cell homeostasis and survival by modulating both membrane and CW composition. Members of the lytR-cpsA-psr superfamily are routinely found as part of the CW stress stimulon, and have been proposed to regulate CW homeostasis and biogenesis [48]. Noteworthy, LytR-CpsA-Psr proteins are present in all Gram-positive organisms, with the exception of the CW-deficient *Mollicutes*, and rarely found in Gram-negative bacteria. In *S. aureus* and *S. mutans*, inactivation of a member of the lytR-CpsA-Psr superfamily resulted in multiple phenotypes including increased susceptibility to CW-active antibiotics [49], [50], [51], [52].

In this study, we unveiled the scope of the CW stress stimulon of *E. faecalis* and conducted a preliminary characterization of four genes encoding putative hypothetical proteins that are unique to Gram-positive bacteria, or exclusively found in enterococci. In addition to the four isogenic mutants characterized here, several other genes with a potential role in CW homeostasis and cell survival were identified and may serve as the foundation for future studies. The identification of the *E. faecalis* CW antibiotic targeting stimulon is an important step for a better understanding of the intrinsic tolerance of enterococci towards CW-damaging agents.

## Materials and Methods

### Bacterial Strains and Growth Conditions

Bacterial strains used in this study are listed in Table 4. Strains were routinely maintained in Brain Heart Infusion (BHI) agar plates (BD, Franklin Lakes, NJ). For microarray analysis, a minimum of 4 independent cultures of *E. faecalis* OG1RF were grown in FMC medium [53] supplemented with 10 mM glucose to an optical density at 600 nm (OD_600_) of 0.3 and the cultures were divided in 5 aliquots. One aliquot was collected by centrifugation and immediately frozen (untreated control cells). The other aliquots were treated for 30 and 60 min with 1.25× the minimum inhibitory concentration (MIC) of one of the following CW inhibitors: ampicillin (20 µg ml^−1^), bacitracin (80 µg ml^−1^), cephalotin (80 µg ml^−1^), or vancomycin (10 µg ml^−1^). To assess the ability of *E. faecalis* OG1RF and its derivative mutant strains to grow in the presence of sub-inhibitory concentrations of antibiotics, mid-exponential-phase (OD_600_ ∼ 0.4) cultures were diluted 1∶100 into conditioned BHI media and growth was monitored for 24 hours using a Bioscreen C growth monitor (Oy Growth Curves AB Ltd., Helsinki, Finland).

**Table 4:** Bacterial strains used in this study.

Strains	Relevant Characteristics	Source or reference
***Enterococcus faecalis***		
OG1RF	Laboratory strain; Rifampicin-R Fusisic Acid-R	Lab stock
CK111/pCF10-101	OG1Spectomycin *upp4::*P23repA4	[55]
ΔEF3245	Deletion mutant of OGIRF	This Study
ΔEF0797	Deletion mutant of OGIRF	This Study
ΔEF1533	Deletion mutant of OGIRF	This Study
ΔEF0026	Deletion mutant of OGIRF	This Study
***Escherichia coli***		
DH10B	Cloning Host	Lab stock
EC1000	Host for cloning RepA-dependent plasmid	[56]

### RNA Extraction

To isolate RNA from *E. faecalis*, cells were harvested by centrifugation at 4°C and then treated with the RNA Protect reagent (QIAGEN, Inc., Chatsworth, CA). Total RNA was isolated from homogenized *E. faecalis* cells by the hot acid-phenol method as described previously [16]. RNA pellets were resuspended in nuclease-free H_2_O, and treated with DNase I (Ambion) at 37°C for 30 minutes. The RNA was purified again using the RNeasy mini kit (QIAGEN), including a second on-column DNase treatment that was performed as recommended by the supplier.

### Microarray Experiments

Transcriptome analysis was performed using the *E. faecalis* microarrays provided by the J. Craig Venter Institute Pathogen Functional Genomics Resource Center (PFGRC) (http://pfgrc.jcvi.org/index.php/microarray). The microarray slides consist of 6600, 70mer DNA probes encompassing all unique open reading frames (ORFs) from six sequenced *E. faecalis* strains: OG1RF, ATCC 29200, TUSoD Ef11, HH22, TX0104, TX1322, and V583 including its three plasmids printed in duplicate. Additional details regarding the arrays can be found at http://pfgrc.jcvi.org/index.php/microarray/array_description/enterococcus_faecalis/version1.html.

A reference RNA prepared from a large-scale culture of *E. faecalis* OG1RF cells that had been grown in BHI to an OD_600_ of 0.5 was used in every experiment. The benefits of the use of a reference RNA as a normalization tool in microarray studies is detailed elsewhere [54]. Four individual cDNA samples originating from four independent cultures of each strain grown under control (FMC) and experimental conditions (FMC containing selected antibiotic) were hybridized to the arrays along with reference cDNA. Test cDNA was coupled with Cy3-dUTP, while reference cDNA was coupled with Cy5-dUTP (Amersham Biosciences). A mixture containing labeled test and reference cDNAs was used to hybridize the slides for 16 h at 42°C in a MAUI hybridization chamber (BioMicro Systems, Salt Lake City, UT). Hybridized slides were washed and scanned using a GenePix scanner (Axon Instruments, Union City, CA). Single-channel images of the slides were loaded into Spotfinder and overlaid. MIDAS software was used to normalize the spot intensity data using LOWESS and iterative log mean centering with default setting, followed by in-slide replicate analysis. Statistical analysis was carried out with BRB array tools (http://linus.nci.nih.gov/BRB-ArrayTools.html) with a cutoff *P* value of 0.01. Additional details regarding array protocols are available at http://pfgrc.jcvi.org/index.php/microarray/protocols.html.

### Microarray Data Accession Number

Microarray data have been deposited in the NCBI Gene Expression Omnibus (GEO) database (http://www.ncbi.nlm.nih.gov/geo) under GEO Series accession number GSE45306.

### Real-time Quantitative PCR

Microarrays were validated by real-time quantitative RT-PCR (qRT-PCR) by using primers specific for the six genes chosen for inactivation and three other randomly selected genes. Gene-specific primers (Table S3) were designed using Beacon Designer 2.0 software (Premier Biosoft International). Reverse transcription and real-time reverse transcriptase PCR were carried out according to protocols described elsewhere [55]. Student’s *t* test was performed to verify significance of the real-time PCR quantifications.

### Construction of Mutant Strains

A markerless genetic exchange system [55] was used to obtain in-frame isogenic deletion strains in *E. faecalis* OG1RF. Briefly, two PCR products flanking the gene of interest were obtained with the primers listed in Table 5. The PCR products of the 5′ and 3′ flanking regions of each gene were digested with restriction enzymes (XbaI and BamHI or BamHI and NcoI) and individually ligated to the previously digested pCJK47 vector [56] using *E. coli* EC1000 [56] as the host strain. The resulting plasmids were electroporated into competent *E. faecalis* CK111/pCF10-101 (donor strain). Donor strains harboring the desired pCJK47 derivative plasmid were conjugated with *E. faecalis* OG1RF (recipient strain) and transconjugants were selected on BHI agar medium containing rifampin (200 µg ml^−1^), fusidic acid (25 µg ml^−1^), and erythromycin (10 µg ml^−1^). The PheS* negative counter-selection system was utilized to isolate double-crossover deletions [56]. Individual gene deletions were confirmed by PCR and further sequencing of the deletion site and flanking regions.

**Table 5:** Primers used for gene deletion.

Gene Locus	Direction	Sequence
EF3245	Forward	CGATAGCGATCTAGAACTTTGTGC
	Reverse	AGCTTCGTCCGGATCCTGAAACCA
	Forward	GGGAATGAAGGGATCCCTTATCAAC
	Reverse	GCTACAATGCCATGGAAAAGAGAGG
EF0797	Forward	GCATAATCATCTAGACTTTCTAGCG
	Reverse	CGGTTGATACGGATCCTGATGATAA
	Forward	CGCTAAGACAGGATCCGATAAGAGA
	Reverse	GATTTCCTCCCATGGTACTACTCG
EF1533	Forward	GCCTCAGACTCTAGAATTGCATTG
	Reverse	GGTCTTGTTGGATCCCTTCATACG
	Forward	GCATCAATGAGGATCCTTGGATAATC
	Reverse	GAAATACTTCCATGGCCCATGTGA
EF0026	Forward	TTGTTCATCTCTAGAAGATCGTCG
	Reverse	CCACTAAAAGGGATCCTATAAACATTG
	Forward	GGAAATAATGGATCCTGCCGCCG
	Reverse	CACGATGAAACCATGGGACACCTTG

The underlined bases correspond to the restriction sites included to aid in the subsequent cloning of the PCR products.

### Minimum Inhibitory Concentration (MIC)

The MIC of selected CW inhibitors (ampicillin, bacitracin, cephalotin and vancomycin) to OG1RF and its derivative mutants was determined by a broth microdilution method in triplicates. Briefly, 3 µl of exponentially-grown cultures (OD_600 nm_ ∼ 0.7) was added to the wells of microtiter plates containing two-fold serial dilutions of antibiotics in BHI. Plates were incubated aerobically at 37°C for 18 h. The lowest concentration of antibiotic that prevented growth was recorded as the MIC.

### Antibiotic Time-kill Kinetics

Cultures were grown in BHI to exponential phase, serially diluted in fresh BHI to obtain 5×10^6^ to 1×10^7^ CFU ml^−1^. Time-kill studies were initiated by adding 5 or 10 times the MIC for each antibiotic for each mutant strain of ampicillin (80 µg ml^−1^), bacitracin (80, 160 and 320 µg ml^−1^), chloramphenicol (80 µg ml^−1^), or vancomycin (40, 80, 160 µg ml^−1^). Viable counts were determined by plating cultures on BHI plates at time zero and then every 24 h. All experiments were performed at least in triplicates and Student’s *t* test was performed to verify significance at each time-point.

### Galleria Mellonella Infection

For the *G. mellonella* killing assays, insects in the final larval stage were purchased from Vanderhorst Inc (St. Marys, OH). Groups of 20 larvae, ranging from 200 to 300 mg in weight with no signs of melanization were randomly chosen and used for subsequent infection. A Hamilton syringe (Hamilton, Reno, NV) was used to inject 5 µl aliquots of bacterial inoculum (5×10^5^ CFU) into the haemocoel of each larva via the last left proleg. Aliquots of the initial inocula were serially diluted and plated onto BHI agar plates to confirm initial infection dose. Control groups were injected with heat-inactivated *E. faecalis* OG1RF (45 min at 75°C). After injection, larvae were kept at 37°C, and monitored for death at various time points during the course of 72 h. Kaplan–Meier killing curves were plotted and estimation of differences in survival compared by using the Mantel-Cox log-rank test. All data were analyzed with GraphPad Prism 4.0 software. Experiments were performed independently at least three times.

## Supporting Information

Figure S1 Growth curve of *E. faecalis* OG1RF in FMC. When cultures reached OD_600_ of 0.3, ampicilin (20 µg ml^−1^), bacitracin (80 µg ml^−1^), cephalotin (40 µg ml^−1^) or vancomycin (10 µg ml^−1^) were added to culture aliquots. For microarray analysis, cells were harvested at 0, 30 and 60 min post antibiotic exposure.
(PDF)

Figure S2 Venn diagrams depicting overlaps in gene expression for each antibiotic between 30 and 60 minutes. Blue numbers indicate upregulated genes and numbers in red represent downregulated genes.
(PDF)

Figure S3 Cell death kinetics of the mutant strains with altered MICs. Experiments were performed using antibiotic concentrations ranging from 5 to 10X the MIC for each mutant strain. Experiments were performed in triplicates with average and standard deviations calculated for each time-point. Student’s *t* test was performed to verify significance (*p*<0.05) in comparison to the parent strain.
(PDF)

Table S1 Genes differentially expressed in *Enterococcus faecalis* OG1RF cells treated with cell-wall inhibiting antibiotics for 30 and 60 min at a *p*-value <0.01.
(DOCX)

Table S2 Validation of microarrays by qRT-PCR. Data is presented in fold of change relative to control conditions measured by qRT-PCR, and by microarrays in parenthesis.
(DOCX)

Table S3 Quantitative RT-PCR primers used to validate microarray data.
(DOCX)
