# Supplementary figures and images for: The Cell Wall-Targeting Antibiotic Stimulon of Enterococcus faecalis
Source: PLoS One. 2013 Jun 3;8(6):e64875. doi: 10.1371/journal.pone.0064875 (PMC3670847; doi:10.1371/journal.pone.0064875)

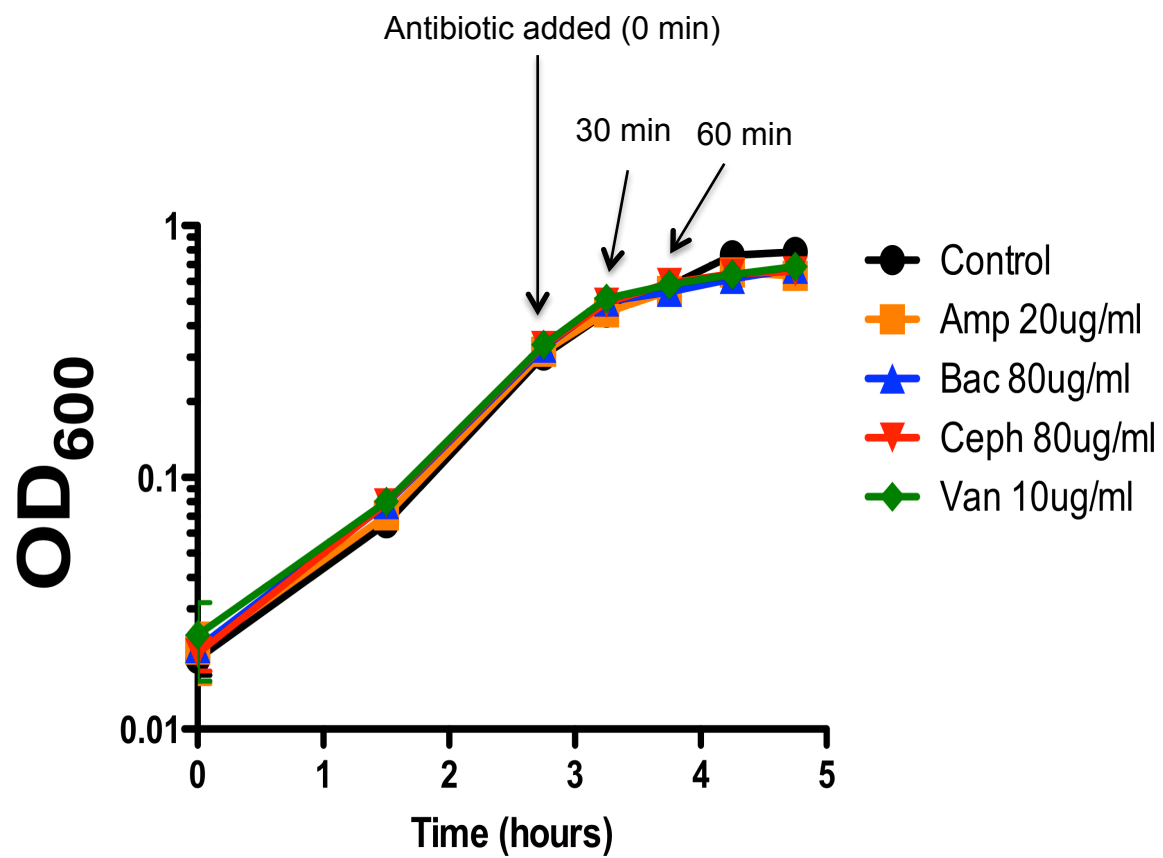

Supplement: Figure S1 — Growth curve of E. faecalis OG1RF in FMC. When cultures reached OD600 of 0.3, ampicilin (20 µg ml−1), bacitracin (80 µg ml−1), cephalotin (40 µg ml−1) or vancomycin (10 µg ml−1) were added to culture aliquots. For microarray analysis, cells were harvested at 0, 30 and 60 min post antibiotic exposure. (PDF) [file pone.0064875.s001.pdf]

Ampicilin

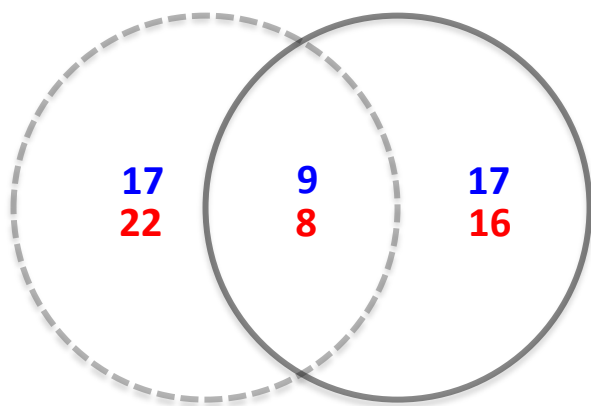

Bacitracin

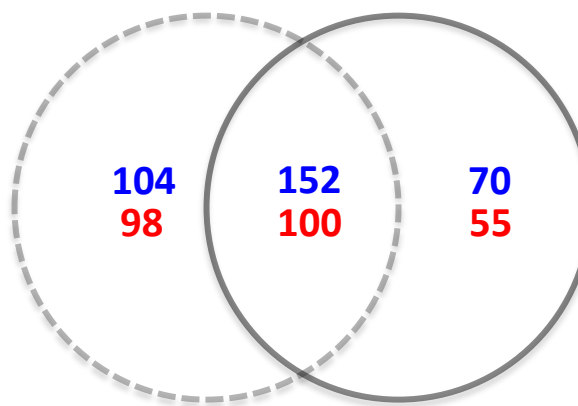

Cephalotin

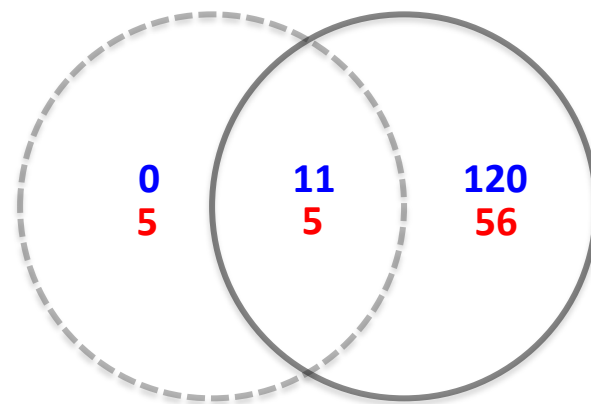

Vancomycin

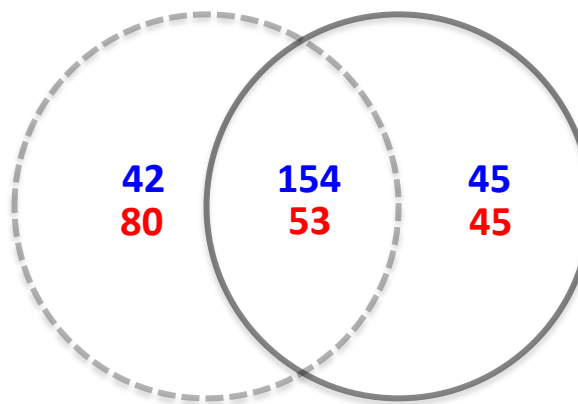

----- 30 min  
———— 60 min

Supplement: Figure S2 — Venn diagrams depicting overlaps in gene expression for each antibiotic between 30 and 60 minutes. Blue numbers indicate upregulated genes and numbers in red represent downregulated genes. (PDF) [file pone.0064875.s002.pdf]

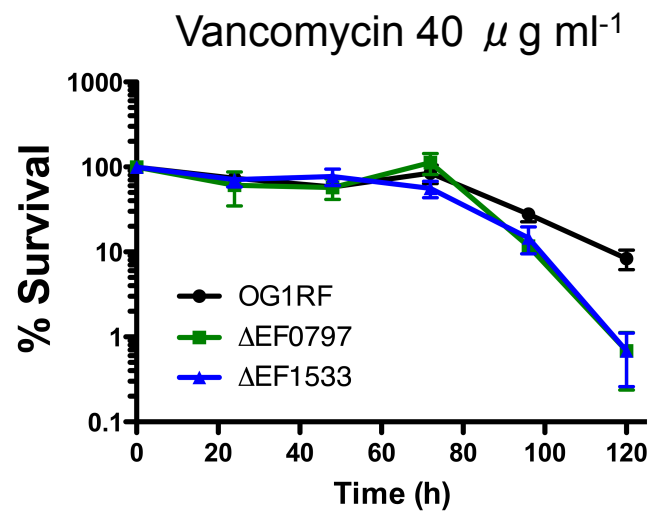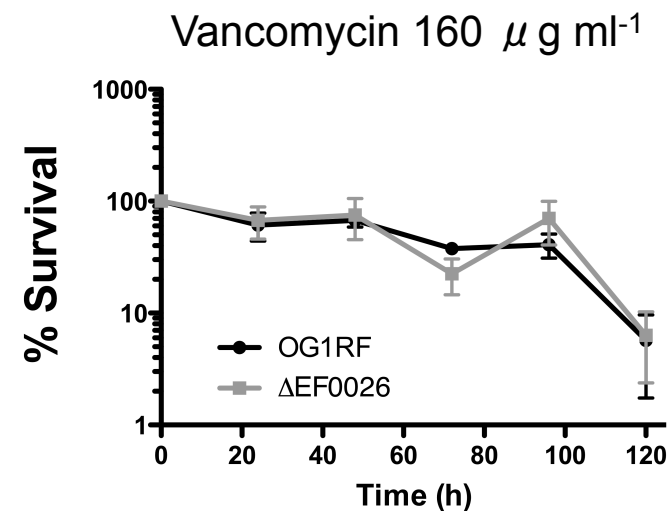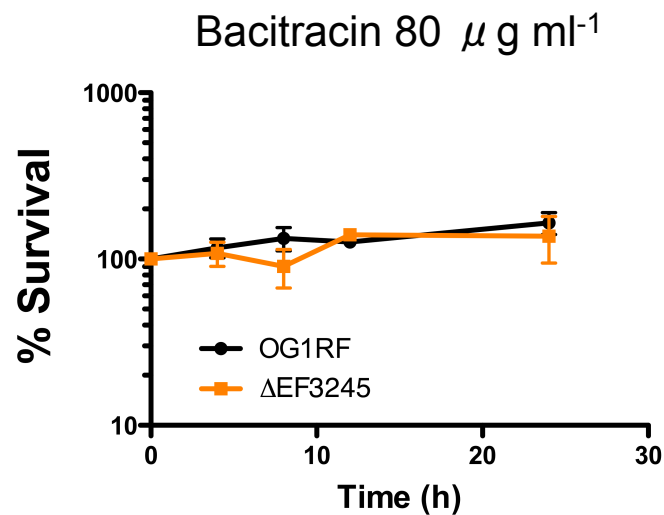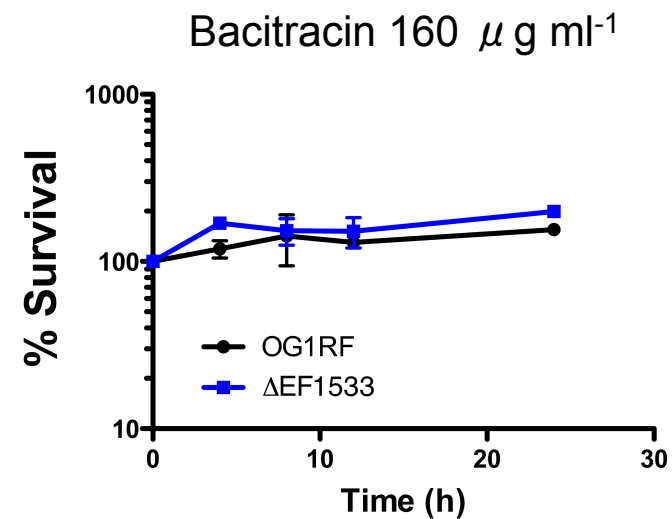

Supplement: Figure S3 — Cell death kinetics of the mutant strains with altered MICs. Experiments were performed using antibiotic concentrations ranging from 5 to 10X the MIC for each mutant strain. Experiments were performed in triplicates with average and standard deviations calculated for each time-point. Student’s t test was performed to verify significance (p<0.05) in comparison to the parent strain. (PDF) [file pone.0064875.s003.pdf]
