# Supplementary material for: The Cell Wall-Targeting Antibiotic Stimulon of Enterococcus faecalis
Source: PLoS One. 2013 Jun 3;8(6):e64875. doi: 10.1371/journal.pone.0064875 (PMC3670847; doi:10.1371/journal.pone.0064875)
Supplement: Table S1 — Genes differentially expressed in Enterococcus faecalis OG1RF cells treated with cell-wall inhibiting antibiotics for 30 and 60 min at a p-value <0.01. (DOCX) [file pone.0064875.s004.docx]

**Table S1** - Genes differentially expressed in *Enterococcus faecalis* OG1RF cells treated with cell-wall inhibiting antibiotics for 30 and 60 min at a *p*-value < 0.01.

| **Gene Number** | **Description** | **Gene symbol** | **Amp 30’** | **Amp 60’** | **Bac**  **30’** | **Bac**  **60’** | **Cep 30’** | **Cep**  **60’** | **Van**  **30’** | **Van**  **60’** | **Annotation** |
| --- | --- | --- | --- | --- | --- | --- | --- | --- | --- | --- | --- |
| OG1RF_12079 | membrane protein |  | 3.46999E+11 | 1591452074 |  |  | 1707113391 | 1592472158 |  |  | cell envelope |
| EF1301 | bacterial cell division protein FtsW | ftsW |  |  |  | 2.04942E+14 |  | 404217610.9 |  |  | replication |
| **EF1533** | conserved hypothetical protein |  | 6.872 | 11.77 | 62.153 | 38.181 | 7.469 | 38.962 | 13.642 | 21.615 | hypothetical |
| EF3245 | transcriptional regulator |  | 4.287 | 8.766 | 43.912 | 30.517 | 5.517 | 34.137 | 81.303 | 112.665 | cell envelope |
| EF0802 | conserved hypothetical protein |  |  | 6.266 | 36.558 | 16.211 |  | 29.287 | 134.366 | 232.041 | hypothetical |
| EF2896 | conserved hypothetical protein |  |  | 6.235 | 35.234 | 30.214 |  | 23.387 | 112.373 | 97.228 | hypothetical |
| EF1814 | EmrB/QacA family drug resistance transporter |  |  | 6.015 | 8.054 | 7.867 |  | 14.353 | 14.553 |  | antibiotic resistance |
| EF3239 | conserved hypothetical protein |  | 3.486 | 7.441 | 8.444 | 5.098 |  | 13.763 | 3.406 |  | hypothetical |
| EF1517 | conserved hypothetical protein |  |  |  | 31.738 | 22.986 |  | 12.788 | 27.634 | 23.373 | hypothetical |
| **EF0026** | conserved hypothetical protein |  | 2.684 | 9.071 | 19.062 | 7.644 | 4.201 | 12.177 | 6.175 | 11.904 | hypothetical |
| **EF0797** | conserved hypothetical protein |  | 4.431 | 8.128 | 10.193 | 12.959 | 4.458 | 11.303 | 5.522 | 10.863 | hypothetical |
| EF0634 | decarboxylase |  |  |  |  |  |  | 10.869 |  |  | hypothetical |
| EF1753 | conserved hypothetical protein |  | 4.408 | 3.953 | 25.319 | 18.408 | 3.742 | 10.203 | 11.632 | 18.169 | hypothetical |
| EF3057 | conserved hypothetical protein |  |  | 6.614 | 5.344 |  |  | 9.225 | 4.801 | 7.355 | hypothetical |
| EF0708 | conserved hypothetical protein |  | 3.663 | 2.992 | 15.868 | 5.97 |  | 9.018 | 9.202 | 16.395 | hypothetical |
| EF0297 | transcriptional repressor CopY | [copY](http://www.ncbi.nlm.nih.gov/entrez/query.fcgi?cmd=search&db=gene&term=copY) |  |  |  |  |  | 8.953 |  |  | transcriptional regulator |
| EF1258 | conserved hypothetical protein |  |  | 3.8 | 36.837 | 11.185 |  | 8.478 | 23.697 | 21.473 | hypothetical |
| EF1947 | conserved hypothetical protein |  |  |  | 6.887 | 6.138 | 3.133 | 8.477 | 4.771 |  | hypothetical |
| EF0972 | phosphoesterase |  |  |  | 8.084 | 10.282 |  | 8.462 | 34.931 | 73.492 | DNA repair/recombination |
| EF0798 | conserved hypothetical protein |  |  |  | 13.508 | 15.826 |  | 8.131 | 6.109 | 9.108 | hypothetical |
| EF2750 | conserved hypothetical protein |  |  |  | 30.265 | 7.459 |  | 7.229 | 11.858 | 21.079 | hypothetical |
| EF0298 | copper-exporting ATPase | [actP1](http://www.ncbi.nlm.nih.gov/entrez/query.fcgi?cmd=search&db=gene&term=actP1) |  |  |  |  | 5.434 | 7.159 |  |  | transport and binding |
| EF1518 | soluble lytic murein transglycosylase |  |  |  |  |  |  | 6.984 | 23.239 | 20.299 | cell envelope |
| EF2892 | conserved hypothetical protein |  |  | 3.806 | 7.931 |  |  | 6.712 | 19.787 | 29.85 | hypothetical |
| EF1587 | nudix family phosphohydrolase |  | 2.824 | 2.61 | 23.39 | 11.864 |  | 6.706 | 15.458 | 19.302 | central and intermediate metabolism |
| EF1304 | magnesium-importing ATPase | [mgtA-2](http://www.ncbi.nlm.nih.gov/entrez/query.fcgi?cmd=search&db=gene&term=mgtA-2) | 2.526 |  | 5.21 | 15.981 |  | 6.669 | 24.622 | 80.974 | transport and binding |
| EF1946 | conserved hypothetical protein |  |  |  | 6.028 | 4.572 | 2.851 | 6.536 | 3.924 |  | hypothetical |
| EF3193 | murein hydrolase regulator LrgB |  |  |  | 6.738 | 8.017 |  | 6.454 |  |  | cell envelope |
| EF0469 | protein of hypothetical function DUF477 |  | 6.106 |  |  | 3.562 | 4.681 | 6.406 |  |  | hypothetical |
| EF1412 | conserved hypothetical protein |  |  | 4.182 |  |  |  | 6.157 |  |  | hypothetical |
| EF0746 | possible beta-lactamase |  |  |  | 12.426 | 9.085 |  | 5.736 | 31.989 | 47.89 | antibiotic resistance |
| EF0932 | conserved hypothetical protein |  |  | 3.771 |  |  |  | 5.551 | 8.544 | 17.813 | hypothetical |
| EF1303 | LysR family transcriptional regulator |  |  |  |  | 10.757 |  | 5.476 | 55.719 | 133.592 | transcriptonal regulator |
| EF0443 | peptidoglycan-binding protein |  |  |  | 16.602 | 28.533 | 2.987 | 5.266 | 40.15 | 68.329 | cell envelope |
| EF2697 | 5-bromo-4-chloroindolyl phosphate hydrolysis protein |  |  |  | 26.57 | 7.086 |  | 5.179 | 3.464 | 7.315 | hypothetical |
| EF0747 | protein of hypothetical function DUF1003 |  |  |  | 18.442 | 7.62 |  | 5.165 |  |  | hypothetical |
| EF2734 | dehydrogenase |  |  |  | 12.518 | 6.717 |  | 5.14 | 10.826 | 10.103 | central and intermediate metabolism |
| OG1RF_11451 | possible ABC superfamily ATP binding cassette, membrane protein |  |  | 3.711 |  | 11.128 |  | 5.123 | 20.068 | 19.108 | transport and binding |
| EF2784 | conserved hypothetical protein |  | 2.661 |  | 17.351 | 12.301 |  | 4.958 | 4.933 |  | hypothetical |
| EF0635 | APC family amino acid-polyamine-organocation transporter |  |  |  |  |  |  | 4.854 |  |  | transport and binding |
| EF1264 | phosphatidylglycerol--membrane-oligosaccharide glycerophosphotransferase |  |  |  | 4.337 | 5.939 |  | 4.459 |  | 6.572 | cell envelope |
| EF1300 | FtsW/RodA/SpoVE family cell division protein |  |  |  |  | 14.367 |  | 4.447 | 10.237 | 36.395 | replication |
| EF0299 | copper transport protein CopZ | [copZ](http://www.ncbi.nlm.nih.gov/entrez/query.fcgi?cmd=search&db=gene&term=copZ) |  |  |  |  |  | 4.421 |  |  | transport and binding |
| EF0440 | POT family proton (H+)-dependent oligopeptide transporter |  |  | 2.152 |  |  |  | 4.37 | 0.074 |  | transport and binding |
| EF1903 | integral membrane protein |  |  |  |  |  |  | 4.247 | 2.376 | 3.018 | cell envelope |
| EF3058 | protein-tyrosine-phosphatase | [yfkJ](http://www.ncbi.nlm.nih.gov/entrez/query.fcgi?cmd=search&db=gene&term=yfkJ) |  |  |  |  |  | 4.234 | 4.039 | 6.345 | aa metabolism |
| EF1904 | glycerophosphoryl diester phosphodiesterase family protein |  |  |  | 4.372 | 3.188 |  | 4.229 | 2.104 |  | cell envelope |
| EF1352 | magnesium-importing ATPase | [mgtA-1](http://www.ncbi.nlm.nih.gov/entrez/query.fcgi?cmd=search&db=gene&term=mgtA-1) |  |  | 2.95 | 3.65 |  | 4.153 | 3.373 | 8.68 | transport and binding |
| EF1751 | membrane protein of hypothetical function |  |  |  | 9.26 | 4.177 |  | 4.073 | 7.764 | 8.539 | cell envelope |
| EF2703 | LytR family transcriptional regulator |  |  |  | 6.549 | 18.974 |  | 4.062 | 8.705 | 27.749 | transcriptional reguralor |
| EF0636 | Na+/H+ antiporter | [nhaC-2](http://www.ncbi.nlm.nih.gov/entrez/query.fcgi?cmd=search&db=gene&term=nhaC-2) |  |  |  |  |  | 4.001 |  |  | transport and binding |
| EF0783 | acyltransferase |  |  |  | 4.565 |  |  | 3.947 | 8.589 | 16.008 | Central and Intermediate metabolism |
| EF0926 | response regulator |  |  |  | 11.853 | 5.341 |  | 3.921 | 2.01 |  | Two component system |
| EF1685 | hemolysin III | [hlyIII](http://www.ncbi.nlm.nih.gov/entrez/query.fcgi?cmd=search&db=gene&term=hlyIII) |  |  | 2.939 | 2.606 |  | 3.921 |  |  | virulence factors |
| EF1302 | transcriptional regulator |  |  |  |  | 6.996 |  | 3.855 | 36.949 | 75.321 | transcriptional regulator |
| *EF1534* | peptidylprolyl isomerase |  |  |  | 3.42 | 2.895 |  | 3.767 | 4.111 | 7.26 | protein folding |
| EF2911 | LuxR family response regulator |  |  |  | 5.152 | 4.184 |  | 3.731 |  |  | Two component system |
| EF2913 | membrane protein |  |  | 2.242 | 8.4 | 4.169 |  | 3.711 |  | 2.958 | cell envelope |
| EF1198 | ABC superfamily ATP binding cassette transporter, membrane protein |  |  | 3.098 |  |  |  | 3.658 | 8.049 | 17.526 | transport and binding |
| EF0904 | mevalonate kinase |  |  |  | 4.069 | 3.641 |  | 3.618 | 7.304 | 7.543 | Central and Intermediate metabolism |
| EF1340 | sex pheromone cAM373 precursor |  |  |  | 10.865 | 7.097 |  | 3.597 | 5.773 | 3.901 | DNA repair/recombination |
| EF2068 | MFS family major facilitator transporter |  |  |  |  |  |  | 3.569 |  | 10.658 | transport and binding |
| EF1752 | stress-responsive transcriptional regulator |  |  |  |  | 4.436 |  | 3.516 | 7.377 | 10.829 | transcriptonal regulator |
| EF2470 | HD superfamily metal-dependent phosphohydrolase |  |  |  | 6.029 |  |  | 3.473 | 7.671 | 6.398 | hypothetical |
| EF2355 | S14 family endopeptidase Clp | [clpB](http://www.ncbi.nlm.nih.gov/entrez/query.fcgi?cmd=search&db=gene&term=clpB) |  |  | 11.773 | 5.444 |  | 3.426 | 2.681 | 4.516 | peptidase/protease |
| EF0680 | penicillin-binding protein |  |  |  | 13.661 | 7.169 |  | 3.356 | 9.967 | 13.759 | cell envelope |
| EF1199 | hypothetical membrane Spanning protein |  |  |  | 2.61 |  |  | 3.355 | 6.776 | 14.364 | cell envelope |
| EF2428 | PadR family transcriptional regulator |  |  |  |  |  |  | 3.301 | 3.947 | 6.707 | transcriptonal regulator |
| EF2479 | conserved hypothetical protein |  |  |  | 9.405 | 7.939 |  | 3.261 |  | 3.182 | hypothetical |
| EF3289 | response regulator |  |  |  | 10.131 | 6.175 |  | 3.256 | 14.943 | 13.466 | Two component system |
| EF1067 | conserved hypothetical protein |  |  |  |  |  |  | 3.239 | 5.198 |  | hypothetical |
| EF2749 | D-alanine--D-alanyl carrier protein ligase |  |  |  | 12.601 | 3.795 |  | 3.233 | 6.601 | 18.107 | cell envelope |
| OG1RF_10064 | hypothetical protein |  |  |  |  | 3.263 |  | 3.213 |  |  | hypothetical |
| EF0359 | DMT superfamily drug/metabolite transporter | [sugE-1](http://www.ncbi.nlm.nih.gov/entrez/query.fcgi?cmd=search&db=gene&term=sugE-1) |  |  |  |  |  | 3.18 |  |  | transport and binding |
| EF2067 | phospholipid-binding protein |  |  |  | 4.643 | 3.094 |  | 3.149 | 11.872 | 21.25 | cell envelope |
| EF2478 | conserved hypothetical protein |  |  |  | 4.75 | 5.944 |  | 3.136 | 2.005 | 2.982 | hypothetical |
| EF1597 | catalase/peroxidase | [katA](http://www.ncbi.nlm.nih.gov/entrez/query.fcgi?cmd=search&db=gene&term=katA) | 3.491 |  | 5.178 |  |  | 3.07 |  |  | oxidative stress |
| EF1268 | P-ATPase superfamily cation transporter |  |  | 2.097 |  |  |  | 3.052 | 9.389 | 14.447 | transport and binding |
| EF2477 | conserved hypothetical protein |  |  |  | 9.277 | 5.135 |  | 3.048 |  | 2.603 | hypothetical |
| EF0116 | conserved hypothetical protein |  |  |  |  |  |  | 3.037 |  |  | hypothetical |
| EF2857 | penicillin binding protein |  |  |  | 10.764 | 5.103 |  | 3.03 | 6.355 | 9.231 | cell envelope |
| EF1224 | Cro/CI family transcriptional regulator |  |  | 2.962 | 3.517 | 16.545 |  | 3.018 |  | 12.506 | transcriptional regulator |
| EF2476 | penicillin-binding protein |  |  |  | 12.136 | 6.602 |  | 2.984 | 5.15 | 6.803 | cell envelope |
| EF1535 | outer surface protein |  |  |  | 3.593 |  |  | 2.983 |  |  | cell envelope |
| [EF3303](http://www.ncbi.nlm.nih.gov/sites/entrez?db=gene&cmd=search&term=1202146&RID=6CVNCR6601S&log$=geneexplicitprot&blast_rank=2) | myosin-cross-reactive antigen |  |  |  | 2.146 |  |  | 2.967 | 3.967 | 7.442 | hypothetical |
| OG1RF_11094 | flotillin |  |  |  | 4.046 | 6.429 |  | 2.957 |  |  | cell envelope |
| EF1180 | conserved hypothetical protein |  |  |  | 6.074 | 5.757 |  | 2.873 |  |  | hypothetical |
| EF2912 | possible histidine kinase | [liaS](http://www.ncbi.nlm.nih.gov/entrez/query.fcgi?cmd=search&db=gene&term=liaS) |  |  | 5.15 | 6.979 |  | 2.861 | 2.112 | 3.039 | Two component system |
| EF2215 | conserved hypothetical protein |  |  |  | 4.35 | 2.366 |  | 2.75 | 5.639 | 8.369 | hypothetical |
| EF0927 | possible histidine kinase | [yxdK](http://www.ncbi.nlm.nih.gov/entrez/query.fcgi?cmd=search&db=gene&term=yxdK) |  |  | 4.478 | 2.866 |  | 2.729 |  |  | Two component system |
| EF2986 | ABC superfamily ATP binding cassette transporter, ABC protein | [phnC-4](http://www.ncbi.nlm.nih.gov/entrez/query.fcgi?cmd=search&db=gene&term=phnC-4) |  |  | 2.799 | 3.728 |  | 2.727 |  |  | transport and binding |
| EF1263 | conserved hypothetical protein |  |  |  | 4.15 | 3.837 |  | 2.726 | 19.257 | 20.433 | hypothetical |
| EF3290 | sensor histidine kinase |  |  |  | 7.191 | 3.311 |  | 2.719 | 9.234 | 13.063 | Two component system |
| EF0032 | ABC superfamily ATP binding cassette transporter, membrane protein |  |  |  | 3.138 | 2.639 |  | 2.708 | 2.951 | 5.745 | transport and binding |
| EF1314 | aspartate transaminase |  |  |  |  |  |  | 2.665 | 3.832 | 4.495 | aa metabolism |
| EF2501 | arsenate reductase |  |  |  |  |  |  | 2.608 |  |  | oxidative stress |
| EF1768 | ABC superfamily ATP binding cassette transporter, ABC protein |  |  |  |  |  |  | 2.604 |  |  | transport and binding |
| EF3041 | peptide ABC superfamily ATP binding cassette transporter, binding protein |  |  |  |  |  |  | 2.539 |  |  | transport and binding |
| EF3149 | transcription regulation protein |  |  |  | 2.933 | 2.36 |  | 2.528 | 2.586 | 2.731 | transcriptional regulator |
| EF2746 | DltD protein | [dltD](http://www.ncbi.nlm.nih.gov/entrez/query.fcgi?cmd=search&db=gene&term=dltD) |  |  | 17.647 | 4.844 |  | 2.526 | 9.682 | 18.699 | cell envelope |
| EF1726 | CspC family cold shock transcriptional regulator |  |  |  |  |  |  | 2.498 |  |  | transcriptonal regulator |
| EF1363 | hydroxymethylglutaryl-CoA synthase |  |  |  |  |  |  | 2.495 | 6.405 | 11.838 | cofactor biosynthesis |
| EF1491 | ribonucleotide reductase family protein |  |  |  | 3.007 | 3.812 |  | 2.484 |  |  | hypothetical |
| EF2195 | family 2 glucosyltransferase |  |  |  | 2.669 | 2.81 |  | 2.47 | 3.054 | 4.626 | glycosyltransferase |
| OG1RF_11113 | hypothetical protein |  |  |  | 3.445 | 3.858 |  | 2.436 |  |  | hypothetical |
| EF2362 | phosphoribosylaminoimidazole carboxylase, ATPase subunit | [purK-2](http://www.ncbi.nlm.nih.gov/entrez/query.fcgi?cmd=search&db=gene&term=purK-2) |  |  | 4.877 | 3.045 |  | 2.424 |  |  | purine metabolism |
| EF0665 | conserved hypothetical protein |  |  | 2.261 |  | 2.385 |  | 2.414 |  |  | hypothetical |
| EF2985 | ABC superfamily ATP binding cassette transporter, membrane protein |  |  |  | 2.239 | 2.847 |  | 2.409 |  |  | transport and binding |
| EF1032 | excinuclease subunit A | [drrC](http://www.ncbi.nlm.nih.gov/entrez/query.fcgi?cmd=search&db=gene&term=drrC) |  |  |  | 2.361 |  | 2.409 |  |  | DNA repair/recombination |
| EF3301 | conserved hypothetical protein |  |  |  |  |  |  | 2.401 | 2.729 |  | hypothetical |
| EF2677 | competence negative regulator MecA |  |  |  | 3.862 |  |  | 2.329 |  | 2.648 | transcriptonal regulator |
| EF1364 | acetyl-CoA acetyltransferase/HMG-CoA reductase |  |  |  | 4.605 |  |  | 2.286 | 5.846 | 15.085 | cofactor biosynthesis |
| OG1RF_11599 | possible GntR family transcriptional regulator |  |  |  | 3.142 | 2.562 |  | 2.267 |  |  | transcriptonal regulator |
| EF1500 | V family ATP synthase subunit D |  |  |  |  |  |  | 2.236 |  |  | energy metabolism |
| EF2863 | Mannosyl-glycoprotein endo-beta-N-acetylglucosaminidase |  |  |  |  |  |  | 2.228 | 6.373 |  | cell envelope |
| EF0859 | CDF family cation diffusion facilitator |  |  |  |  |  |  | 2.21 |  |  | transport and binding |
| OG1RF_12096 | possible membrane protein |  |  |  |  | 2.538 |  | 2.208 | 3.082 |  | cell envelope |
| EF0256 | peptidyl-tRNA hydrolase | [pth](http://www.ncbi.nlm.nih.gov/entrez/query.fcgi?cmd=search&db=gene&term=pth) |  |  |  |  |  | 2.207 | 2.409 |  | translation |
| OG1RF_11803 | regulator of penicillin binding proteins and beta lactamase transcription | bolA |  |  | 2.549 | 3.607 |  | 2.186 |  |  | transcriptional regulator |
| EF0188 | iron (Fe3+) ABC superfamily ATP binding cassette transporter, binding protein |  |  |  |  |  |  | 2.184 | 6.768 | 6.732 | transport and binding |
| EF1573 | conserved hypothetical protein |  |  |  | 4.227 | 3.503 |  | 2.119 | 2.529 | 2.746 | hypothetical |
| EF2183 | ABC superfamily ATP binding cassette transporter, membrane protein |  |  |  | 4.079 | 2.67 |  | 2.11 | 2.514 | 4.934 | transport and binding |
| EF2369 | conserved hypothetical protein |  |  |  | 2.633 | 2.484 |  | 2.105 |  |  | hypothetical |
| EF0698 | acetyltransferase |  |  |  |  |  |  | 2.103 |  |  | Central and Intermediate metabolism |
| EF0902 | phosphomevalonate kinase |  |  |  | 3.743 | 3.012 |  | 2.102 | 6.682 | 6.617 | Central and Intermediate metabolism |
| EF2190 | group 2 family glycosyl transferase |  |  |  |  |  |  | 2.095 | 2.625 | 4.479 | glycosyltransferase |
| EF0748 | rhodanese domain sulfurtransferase |  |  |  | 3.318 |  |  | 2.047 | 2.437 |  | hypothetical |
| EF0901 | isopentenyl pyrophosphate isomerase | [fni](http://www.ncbi.nlm.nih.gov/entrez/query.fcgi?cmd=search&db=gene&term=fni) |  |  | 2.167 |  |  | 2.025 | 4.883 | 4.9 | Central and Intermediate metabolism |
| EF2445 | 2-dehydropantoate 2-reductase |  |  |  | 2.441 |  |  | 2.014 | 4.635 | 6.124 | cofactor biosynthesis |
| EF1679 | S41A family carboxy-terminal peptidase |  |  |  |  | 2.526 |  | 1.976 |  |  | peptidase/protease |
| EF0259 | RNA-binding heat shock protein |  |  |  |  | 2.648 |  | 1.968 | 2.391 | 2.773 | stress |
| EF3152 | large conductance mechanosensitive channel protein | [mscL](http://www.ncbi.nlm.nih.gov/entrez/query.fcgi?cmd=search&db=gene&term=mscL) |  |  | 0.338 | 0.376 |  | 0.51 | 0.204 | 0.244 | transport and binding |
| EF2835 | conserved hypothetical protein |  |  |  |  |  |  | 0.507 |  |  | hypothetical |
| EF3163 | ribose-phosphate pyrophosphokinase | [prs1](http://www.ncbi.nlm.nih.gov/entrez/query.fcgi?cmd=search&db=gene&term=prs1) |  |  | 0.22 | 0.276 |  | 0.502 |  | 0.469 | Central and Intermediate metabolism |
| EF0235 | conserved hypothetical protein |  |  |  | 0.22 |  |  | 0.489 |  |  | hypothetical |
| EF3048 | cellobiose PTS family porter CelC |  |  |  |  |  |  | 0.486 |  |  | energy metabolism |
| EF2651 | spermidine/putrescine ABC superfamily ATP binding cassette transporter, membrane protein |  |  |  | 0.189 |  |  | 0.458 |  |  | transport and binding |
| EF0227 | preprotein translocase subunit SecY | [secY](http://www.ncbi.nlm.nih.gov/entrez/query.fcgi?cmd=search&db=gene&term=secY) |  |  |  |  |  | 0.458 |  |  | secretion |
| EF1820 | accessory gene regulator protein C |  |  |  | 0.347 |  |  | 0.452 |  |  | Two component system |
| EF0197 | ribose-5-phosphate isomerase A | [rpiA](http://www.ncbi.nlm.nih.gov/entrez/query.fcgi?cmd=search&db=gene&term=rpiA) |  |  | 0.157 | 0.249 |  | 0.448 | 0.315 | 0.456 | energy metabolism |
| EF2047 | APC family amino acid-polyamine-organocation transporter |  |  |  | 0.147 | 0.401 |  | 0.448 |  |  | transport and binding |
| EF0180 | ABC superfamily, ATP binding cassette transporter, membrane protein |  |  |  | 0.244 | 0.447 |  | 0.44 | 0.275 |  | transport and binding |
| EF1967 | conserved hypothetical protein |  |  |  | 0.199 | 0.292 |  | 0.439 |  |  | hypothetical |
| EF0094 | FNT family formate-nitrite transporter |  |  |  | 0.129 | 0.295 |  | 0.438 |  |  | transport and binding |
| EF1643 | glycerol-3-phosphate acyltransferase PlsY |  |  |  | 0.127 | 0.24 |  | 0.436 | 0.161 | 0.385 | cell envelope |
| EF3070 | 30S ribosomal protein S4 | [rpsD](http://www.ncbi.nlm.nih.gov/entrez/query.fcgi?cmd=search&db=gene&term=rpsD) |  |  | 0.23 | 0.258 |  | 0.435 |  |  | Ribosomal |
| EF0372 | response regulator |  |  |  |  |  |  | 0.433 |  |  | two component system |
| EF1248 | conserved hypothetical protein |  |  |  | 0.103 | 0.264 |  | 0.432 |  | 0.313 | hypothetical |
| EF0044 | serine O-acetyltransferase | [cysE](http://www.ncbi.nlm.nih.gov/entrez/query.fcgi?cmd=search&db=gene&term=cysE) |  |  | 0.397 |  |  | 0.432 |  |  | aa metabolism |
| EF1209 | possible histidine kinase | [malK](http://www.ncbi.nlm.nih.gov/entrez/query.fcgi?cmd=search&db=gene&term=malK) |  |  |  | 0.335 |  | 0.429 |  |  | Two component system |
| EF0025 | membrane protein |  |  |  | 0.139 | 0.229 |  | 0.424 | 0.264 |  | cell envelope |
| EF0058 | purine operon repressor | [purR](http://www.ncbi.nlm.nih.gov/entrez/query.fcgi?cmd=search&db=gene&term=purR) |  |  | 0.151 | 0.241 |  | 0.418 | 0.269 | 0.273 | transcriptional regulator |
| EF1748 | prolipoprotein diacylglyceryl transferase | [lgt](http://www.ncbi.nlm.nih.gov/entrez/query.fcgi?cmd=search&db=gene&term=lgt) |  | 0.4 |  |  |  | 0.418 |  |  | cell envelope |
| EF3015 | DAACS family dicarboxylate/amino acid:sodium (Na+) symporter |  |  |  | 0.313 | 0.274 |  | 0.416 |  |  | transport and binding |
| EF2372 | aspartate aminotransferase | [aspB](http://www.ncbi.nlm.nih.gov/entrez/query.fcgi?cmd=search&db=gene&term=aspB) |  |  |  |  |  | 0.414 |  |  | aa metabolism |
| EF0097 | transcriptional regulator |  |  |  | 0.167 |  |  | 0.409 | 0.189 |  | transcriptional regulator |
| EF0790 | multidrug resistance ABC superfamily ATP binding cassette transporter, membrane protein |  |  | 0.434 |  |  |  | 0.406 |  |  | antibiotic resistance |
| EF2151 | D-fructose-6-phosphate amidotransferase | [glmS](http://www.ncbi.nlm.nih.gov/entrez/query.fcgi?cmd=search&db=gene&term=glmS) |  |  | 0.325 |  |  | 0.404 | 0.253 |  | energy metabolism |
| EF0910 | oligopeptide ABC superfamily ATP binding cassette transporter, membrane protein |  |  |  |  |  |  | 0.401 | 0.306 | 0.395 | transport and binding |
| EF1601 | possible protein-N(pi)-phosphohistidine--sugar phosphotransferase | [scrA](http://www.ncbi.nlm.nih.gov/entrez/query.fcgi?cmd=search&db=gene&term=scrA) |  |  |  | 0.347 |  | 0.399 |  |  | energy metabolism |
| EF1716 | carbamoyl phosphate synthase large subunit | [carB](http://www.ncbi.nlm.nih.gov/entrez/query.fcgi?cmd=search&db=gene&term=carB) | 0.394 | 0.411 |  |  |  | 0.397 |  |  | aa metabolism |
| EF0470 | ribonucleotide-diphosphate reductase subunit beta | [nrdF](http://www.ncbi.nlm.nih.gov/entrez/query.fcgi?cmd=search&db=gene&term=nrdF) |  |  |  |  |  | 0.395 |  |  | replication |
| EF0290 | cystathionine gamma-synthase/cystathionine beta-lyase | [metC](http://www.ncbi.nlm.nih.gov/entrez/query.fcgi?cmd=search&db=gene&term=metC) |  |  | 0.136 | 0.276 |  | 0.393 |  |  | aa metabolism |
| EF1567 | shikimate kinase | [aroK](http://www.ncbi.nlm.nih.gov/entrez/query.fcgi?cmd=search&db=gene&term=aroK) |  |  |  |  |  | 0.378 | 0.314 |  | central and intermediate metabolism |
| EF1938 | possible calcium-transporting ATPase |  |  |  | 0.254 | 0.293 |  | 0.37 | 0.416 |  | transport and binding |
| EF0057 | metal cation ABC superfamily ATP binding cassette transporter, membrane protein |  |  |  | 0.29 | 0.318 |  | 0.369 |  |  | transport and binding |
| EF2623 | cadmium-translocating P family ATPase | [cadA](http://www.ncbi.nlm.nih.gov/entrez/query.fcgi?cmd=search&db=gene&term=cadA) |  |  |  |  |  | 0.366 | 0.12 | 0.128 | transport and binding |
| EF2266 | conserved hypothetical protein |  |  |  | 0.235 | 0.315 | 0.339 | 0.366 |  |  | hypothetical |
| EF2904 | beta-lactamase domain protein |  |  | 0.388 | 0.386 |  |  | 0.355 |  |  | antibiotic resistance |
| EF2075 | ABC superfamily ATP binding cassette transporter, membrane protein |  | 0.319 |  |  |  | 0.37 | 0.355 |  |  | transport and binding |
| EF1651 | family U48 amino terminal protease |  | 0.399 |  | 0.393 |  |  | 0.351 |  |  | peptidase/protease |
| EF_B0032 | conserved hypothetical protein |  |  |  |  |  |  | 0.345 |  |  | hypothetical |
| EF0368 | aspartate kinase |  |  |  | 0.342 | 0.34 |  | 0.332 |  |  | aa metabolism |
| EF0289 | cysteine synthase | [cysM](http://www.ncbi.nlm.nih.gov/entrez/query.fcgi?cmd=search&db=gene&term=cysM) |  |  | 0.205 |  |  | 0.319 |  |  | aa metabolism |
| EF2554 | peptide chain release factor 1 | [prfA](http://www.ncbi.nlm.nih.gov/entrez/query.fcgi?cmd=search&db=gene&term=prfA) |  |  | 0.242 | 0.276 |  | 0.318 | 0.48 |  | translation |
| EF1190 | BS_ykrK family protein |  |  |  |  |  |  | 0.313 |  | 0.253 | hypothetical |
| EF1192 | MIP family major intrinsic protein channel protein |  |  |  | 0.217 | 0.184 |  | 0.31 | 0.136 | 0.194 | transport and binding |
| EF3047 | integral membrane protein |  |  |  | 0.286 |  |  | 0.308 |  |  | cell envelope |
| EF3020 | ECF subfamily RNA polymerase sigma-24 factor |  |  |  |  | 0.271 |  | 0.305 |  |  | transcriptional regulator |
| EF3091 | protein of hypothetical function DUF161 |  |  |  | 0.131 | 0.155 |  | 0.303 | 0.249 | 0.388 | hypothetical |
| EF0909 | oligopeptide ABC superfamily ATP binding cassette transporter, membrane protein |  |  |  | 0.289 |  |  | 0.302 | 0.281 | 0.4 | transport and binding |
| EF1582 | MFS family major facilitator transporter |  |  |  | 0.068 | 0.113 |  | 0.298 |  |  | transport and binding |
| EF0243 | branched-chain amino acid transport system II carrier protein | [brnQ](http://www.ncbi.nlm.nih.gov/entrez/query.fcgi?cmd=search&db=gene&term=brnQ) |  |  | 0.108 | 0.145 | 0.406 | 0.279 | 0.114 | 0.133 | transport and binding |
| EF2793 | conserved hypothetical protein |  |  |  | 0.049 | 0.094 | 0.4 | 0.278 | 0.102 | 0.14 | hypothetical |
| EF0633 | tyrosine--tRNA ligase | [tyrS1](http://www.ncbi.nlm.nih.gov/entrez/query.fcgi?cmd=search&db=gene&term=tyrS1) |  |  |  |  |  | 0.276 | 0.357 | 0.24 | translation |
| EF2504 | conserved hypothetical protein |  |  |  |  |  | 0.246 | 0.276 |  |  | hypothetical |
| EF3072 | biotin synthesis protein BioY |  |  |  |  | 0.161 |  | 0.275 |  |  | cofactor biosynthesis |
| EF1591 | AraC family transcriptional regulator |  | 0.266 |  |  |  |  | 0.266 |  |  | transcriptonal regulator |
| EF1247 | conserved hypothetical protein |  |  |  | 0.048 | 0.069 |  | 0.252 |  |  | hypothetical |
| EF0944 | LysM domain protein |  | 0.351 | 0.22 | 0.311 |  |  | 0.247 |  |  | cell envelope |
| EF3090 | isochorismatase family protein |  |  |  |  | 0.113 |  | 0.214 |  |  | Central and Intermediate metabolism |
| EF1384 | conserved hypothetical protein |  |  |  | 0.126 | 0.131 |  | 0.191 | 0.266 |  | hypothetical |
| EF1231 | possible phosphoesterase |  |  |  |  |  |  |  | 35.791 | 67.746 | hypothetical |
| EF2859 | 4-oxalocrotonate tautomerase |  |  |  |  |  |  |  | 29.292 | 25.367 | central and intermediate metabolism |
| OG1RF_12161 | M15 family muramoylpentapeptide carboxypeptidase | [vanY](http://www.ncbi.nlm.nih.gov/entrez/query.fcgi?cmd=search&db=gene&term=vanY) |  |  | 3.935 |  |  |  | 22.17 | 20.601 | cell envelope |
| EF1262 | conserved hypothetical protein |  |  |  | 5.123 | 4.194 |  |  | 16.345 | 17.199 | hypothetical |
| EF1647 | ATP-dependent protease peptidase subunit | [hslV](http://www.ncbi.nlm.nih.gov/entrez/query.fcgi?cmd=search&db=gene&term=hslV) |  |  | 10.465 | 4.881 |  |  | 12.01 | 9.749 | peptidase/protease |
| EF1646 | ATP-dependent protease ATP-binding subunit | [hslU](http://www.ncbi.nlm.nih.gov/entrez/query.fcgi?cmd=search&db=gene&term=hslU) |  |  | 11.056 | 3.997 |  |  | 10.182 | 10.122 | peptidase/protease |
| EF1648 | site-specific recombinase XerD |  |  |  | 4.578 |  |  |  | 9.94 | 11.259 | DNA repair/recombination |
| EF1511 | possible Chloromuconate cycloisomerase | [clcB](http://www.ncbi.nlm.nih.gov/entrez/query.fcgi?cmd=search&db=gene&term=clcB) |  |  |  |  |  |  | 9.918 | 7.252 | aa metabolism |
| EF2747 | D-alanine--poly(phosphoribitol) ligase subunit 2 | [dltC](http://www.ncbi.nlm.nih.gov/entrez/query.fcgi?cmd=search&db=gene&term=dltC) |  |  | 11.037 | 5.959 |  |  | 9.503 | 22.94 | cell envelope |
| EF1645 | transcriptional repressor CodY | [codY](http://www.ncbi.nlm.nih.gov/entrez/query.fcgi?cmd=search&db=gene&term=codY) |  |  | 10.204 | 4.835 |  |  | 8.44 | 7.547 | transcriptonal regulator |
| EF1644 | aldose 1-epimerase |  |  |  |  |  |  |  | 7.567 | 8.059 | energy metabolism |
| EF2750 | D-alanine transfer protein DltB | [dtlB](http://www.ncbi.nlm.nih.gov/entrez/query.fcgi?cmd=search&db=gene&term=dtlB) |  |  | 13.236 | 5.149 |  |  | 7.343 | 18.47 | cell envelope |
| EF1006 | conserved hypothetical protein |  |  |  |  |  |  |  | 7.335 | 13.748 | hypothetical |
| EF0903 | possible diphosphomevalonate decarboxylase | [mvd](http://www.ncbi.nlm.nih.gov/entrez/query.fcgi?cmd=search&db=gene&term=mvd) |  |  | 3.651 | 2.82 |  |  | 6.993 | 7.115 | Central and Intermediate metabolism |
| EF2214 | lactoylglutathione lyase |  |  |  |  |  |  |  | 6.728 | 11.38 | stress |
| EF3203 | 50S ribosomal protein L33 | [rpmG-4](http://www.ncbi.nlm.nih.gov/entrez/query.fcgi?cmd=search&db=gene&term=rpmG-4) |  |  | 12.673 | 5.07 |  |  | 6.499 | 9.276 | Ribosomal |
| EF2985 | conserved hypothetical protein |  |  |  | 64.459 | 10.648 |  |  | 6.271 |  | hypothetical |
| EF2986 | ABC superfamily ATP binding cassette transporter, ABC protein |  |  |  | 7.562 |  |  |  | 6.223 | 5.929 | transport and binding |
| EF1119 | glutamine ABC superfamily ATP binding cassette transporter, binding protein |  |  |  |  |  |  |  | 6.175 |  | transport and binding |
| EF2987A | RND family efflux transporter MFP subunit |  |  |  | 45.774 | 4.187 |  |  | 6.113 |  | transport and binding |
| EF1118 | amino acid ABC superfamily ATP binding cassette transporter, membrane protein |  |  |  | 0.189 |  |  |  | 6.04 |  | transcriptional regulator |
| EF2985 | ABC superfamily ATP binding cassette transporter, membrane protein |  |  |  | 14.515 | 7.258 |  |  | 5.817 |  | transport and binding |
| EF1513 | oligopeptide ABC superfamily ATP binding cassette transporter |  |  |  |  |  |  |  | 5.715 |  | transport and binding |
| EF1512 | transglutaminase domain protein |  |  |  |  |  |  |  | 5.69 | 5.129 | hypothetical |
| EF3202 | 30S ribosomal protein S14 | [rpsN-2](http://www.ncbi.nlm.nih.gov/entrez/query.fcgi?cmd=search&db=gene&term=rpsN-2) |  |  | 5.17 |  |  |  | 5.359 | 5.727 | Ribosomal |
| EF2836 | conserved hypothetical protein |  |  |  | 2.954 |  |  |  | 5.157 |  | hypothetical |
| EF1569 | transcriptional regulator, PSR protein | [psr](http://www.ncbi.nlm.nih.gov/entrez/query.fcgi?cmd=search&db=gene&term=psr) |  |  |  |  |  |  | 4.975 | 7.156 | transcriptional regulator |
| EF3059 | transcriptional regulator |  |  |  |  |  |  |  | 4.953 | 8.205 | transcriptional regulator |
| EF3027 | S1 family peptidase | [htrA](http://www.ncbi.nlm.nih.gov/entrez/query.fcgi?cmd=search&db=gene&term=htrA) |  |  |  |  |  |  | 4.888 | 3.97 | peptidase/protease |
| EF2721 | L-serine dehydratase, iron-sulfur-dependent, beta subunit | [sdhB-2](http://www.ncbi.nlm.nih.gov/entrez/query.fcgi?cmd=search&db=gene&term=sdhB-2) |  |  |  |  |  |  | 4.876 |  | transport and binding |
| EF0819 | conserved hypothetical protein |  |  |  |  |  |  |  | 4.731 | 5.511 | hypothetical |
| EF2698 | tellurite resistance protein |  |  |  | 14 | 13.648 |  |  | 4.631 | 7.813 | resistance |
| EF2427 | conserved hypothetical protein |  |  |  |  |  |  |  | 4.424 | 6.296 | hypothetical |
| EF3235 | gluconokinase | [gntK](http://www.ncbi.nlm.nih.gov/entrez/query.fcgi?cmd=search&db=gene&term=gntK) |  |  |  |  |  |  | 4.353 | 8.11 | energy metabolism |
| EF2589 | adenosylcobyric acid synthase |  |  |  | 3.759 | 2.746 |  |  | 4.349 | 5.045 | energy metabolism |
| EF3000 | allantoin permease |  |  |  |  |  |  |  | 4.313 |  | central and intermediate metabolism |
| EF2733 | UDP-N-acetylenolpyruvoylglucosamine reductase | [murB](http://www.ncbi.nlm.nih.gov/entrez/query.fcgi?cmd=search&db=gene&term=murB) |  |  |  |  |  |  | 3.913 | 4.188 | cell envelope |
| EF3277 | cytosine permease |  |  |  |  |  |  |  | 3.887 |  | transport and binding |
| EF2585 | UDP-N-acetylmuramoylalanyl-D-glutamate--2, 6-diaminopimelate ligase |  |  |  | 3.638 |  |  |  | 3.884 | 5.318 | cell envelope |
| EF1906 | integral membrane protein |  |  |  |  |  |  |  | 3.802 | 6.289 | cell envelope |
| EF1132 | cystathionine beta-synthase (CBS) domain protein |  |  |  | 3.445 |  |  |  | 3.664 | 7.777 | hypothetical |
| EF3234 | lipoprotein |  |  |  |  |  |  |  | 3.66 | 5.473 | cell envelope |
| EF1572 | conserved hypothetical protein |  |  |  | 4.025 | 2.855 |  |  | 3.66 | 3.701 | hypothetical |
| EF1035 | possible lipoprotein |  |  |  | 5.858 | 4.905 |  |  | 3.645 | 8.644 | cell envelope |
| EF1212 | LytR family transcriptional regulator |  |  |  | 5.315 | 2.893 |  |  | 3.543 | 5.283 | transcriptional regulator |
| EF1149 | recombination protein RecU | [recU](http://www.ncbi.nlm.nih.gov/entrez/query.fcgi?cmd=search&db=gene&term=recU) |  |  |  |  |  |  | 3.541 | 3.502 | DNA repair/recombination |
| EF2860 | ErfK/YbiS/YcfS/YnhG family protein |  |  |  |  | 4.41 |  |  | 3.516 | 4.552 | cell envelope |
| EF1523 | conserved hypothetical protein |  |  |  |  |  |  |  | 3.499 | 4.602 | hypothetical |
| EF0258 | PST family polysaccharide transporter |  |  |  |  |  |  |  | 3.463 | 3.962 | energy metabolism |
| EF0260 | conserved hypothetical protein |  |  |  | 2.836 |  |  |  | 3.342 |  | hypothetical |
| EF2217 | alpha-1,2-mannosidase |  |  |  |  |  |  |  | 3.319 | 5.74 | energy metabolism |
| EF1203 | crossover junction endodeoxyribonuclease |  |  |  | 5.606 | 2.753 |  |  | 3.305 | 3.764 | DNA repair/recombination |
| EF1202 | protein of hypothetical function DUF965 |  |  |  | 4.057 |  |  |  | 3.208 | 3.67 | hypothetical |
| OG1RF_12162 | sensor histidine kinase VanSG | [vanSG](http://www.ncbi.nlm.nih.gov/entrez/query.fcgi?cmd=search&db=gene&term=vanSG) |  |  | 5.86 | 3.696 |  |  | 3.153 | 3.347 | Two component system |
| EF1907 | dehydratase |  |  |  |  |  |  |  | 3.135 | 4.515 | central and intermediate metabolism |
| EF2495 | Di-trans,poly-cis-decaprenylcistransferase | [uppS](http://www.ncbi.nlm.nih.gov/entrez/query.fcgi?cmd=search&db=gene&term=uppS) |  |  |  |  |  |  | 3.135 | 4.458 | cell envelope |
| EF2722 | L-serine dehydratase, iron-sulfur-dependent, alpha subunit | [sdhA-2](http://www.ncbi.nlm.nih.gov/entrez/query.fcgi?cmd=search&db=gene&term=sdhA-2) |  |  |  |  |  |  | 3.101 | 6.574 | transport and binding |
| EF1810 | family 8 glycosyltransferase | [gspA-1](http://www.ncbi.nlm.nih.gov/entrez/query.fcgi?cmd=search&db=gene&term=gspA-1) |  |  |  |  |  |  | 3.092 |  | glycosyltransferase |
| EF1677 | lipoprotein |  |  |  | 7.902 | 4.671 |  |  | 3.084 |  | cell envelope |
| EF1368 | conserved hypothetical protein |  |  |  |  | 3.512 |  |  | 3.057 | 3.845 | hypothetical |
| EF2193 | dTDP-4-dehydrorhamnose 3,5-epimerase | [rfbC](http://www.ncbi.nlm.nih.gov/entrez/query.fcgi?cmd=search&db=gene&term=rfbC) |  |  | 2.158 | 2.143 |  |  | 3.017 | 3.527 | cell envelope |
| EF2194 | glucose-1-phosphate thymidylyltransferase | [rfbA](http://www.ncbi.nlm.nih.gov/entrez/query.fcgi?cmd=search&db=gene&term=rfbA) |  |  |  |  |  |  | 3.005 | 4.062 | cell envelope |
| EF3275 | hydantoinase/oxoprolinase |  |  |  |  |  |  |  | 2.994 |  | aa metabolism |
| EF1037 | aspartate aminotransferase |  |  |  |  |  |  |  | 2.976 |  | aa metabolism |
| EF2929 | ABC superfamily ATP binding cassette transporter, membrane protein |  |  |  | 3.536 | 2.386 |  |  | 2.957 | 3.17 | transport and binding |
| EF0267 | tRNA-dihydrouridine synthase |  |  |  | 5.192 |  |  |  | 2.955 |  | translation |
| EF0644 | LysR family transcriptional regulator |  |  |  |  |  |  |  | 2.941 | 2.559 | transcriptional regulator |
| EF2050 | ABC superfamily ATP binding cassette transporter, ABC protein | [phnC-2](http://www.ncbi.nlm.nih.gov/entrez/query.fcgi?cmd=search&db=gene&term=phnC-2) |  |  | 50.025 | 5.306 |  |  | 2.937 |  | transport and binding |
| EF2432 | hydroxyacylglutathione hydrolase |  |  |  | 3.35 |  |  |  | 2.906 | 3.758 | antibiotic resistance |
| EF0063 | oligopeptide ABC superfamily ATP binding cassette transporter, binding protein |  |  |  |  |  |  |  | 2.9 |  | transport and binding |
| EF0477 | conserved hypothetical protein |  |  |  | 0.099 | 0.123 |  |  | 2.887 |  | hypothetical |
| EF1261 | histidine kinase | [yclK](http://www.ncbi.nlm.nih.gov/entrez/query.fcgi?cmd=search&db=gene&term=yclK) |  |  |  |  |  |  | 2.839 | 4.625 | Two component system |
| EF1727 | ebsA protein | [ebsa](http://www.ncbi.nlm.nih.gov/entrez/query.fcgi?cmd=search&db=gene&term=ebsa) |  |  |  |  |  |  | 2.833 | 3.533 | cell envelope |
| EF1148 | peptidoglycan glycosyltransferase |  |  |  |  |  |  |  | 2.796 | 3.729 | cell envelope |
| EF1204 | protein of hypothetical function DUF1292 |  |  |  | 4.058 | 2.952 |  |  | 2.792 | 2.426 | hypothetical |
| EF1135 | MscS family small conductance mechanosenstive ion channel protein |  |  |  | 2.613 |  |  |  | 2.757 | 4.081 | hypothetical |
| EF0453 | organic hydroperoxide reductase |  |  |  |  | 2.679 |  |  | 2.754 |  | stress |
| EF3150 | M16C subfamily protease |  |  |  | 2.645 |  |  |  | 2.752 | 2.677 | peptidase/protease |
| EF2643 | conserved hypothetical protein |  |  |  |  |  |  |  | 2.737 | 2.871 | hypothetical |
| EF0366 | protein of hypothetical function DUF72 |  |  |  | 4.02 | 3.632 |  |  | 2.648 | 4.289 | hypothetical |
| EF1403 | colicin V production protein |  |  |  |  |  |  |  | 2.636 | 2.276 | hypothetical |
| EF2752 | ABC superfamily ATP binding cassette transporter, ABC protein | [phnC-1](http://www.ncbi.nlm.nih.gov/entrez/query.fcgi?cmd=search&db=gene&term=phnC-1) |  |  | 5.554 | 2.621 |  |  | 2.626 |  | transport and binding |
| EF2658 | MurM family protein |  |  |  | 5.487 |  |  |  | 2.597 | 5.023 | cell envelope |
| EF2425 | phosphoglucomutase | [pgcA](http://www.ncbi.nlm.nih.gov/entrez/query.fcgi?cmd=search&db=gene&term=pgcA) |  |  | 5.724 | 3.833 |  |  | 2.592 | 2.65 | energy metabolism |
| EF0994 | acetylglucosaminyltransferase | [murG](http://www.ncbi.nlm.nih.gov/entrez/query.fcgi?cmd=search&db=gene&term=murG) |  |  | 4.552 | 4.304 |  |  | 2.574 | 2.554 | cell envelope |
| EF1039 | HAD superfamily hydrolase |  |  |  |  |  |  |  | 2.565 |  | hypothetical |
| EF0882 | possible replicative DNA helicase DnaB |  |  |  | 3.128 |  |  |  | 2.553 | 3.071 | transcriptional regulator |
| EF2197 | family 2 glycosyl transferase |  |  |  |  |  |  |  | 2.522 | 4.933 | glycosyltransferase |
| EF2494 | phosphatidate cytidylyltransferase | [cdsA](http://www.ncbi.nlm.nih.gov/entrez/query.fcgi?cmd=search&db=gene&term=cdsA) |  |  |  |  |  |  | 2.522 | 3.414 | cell envelope |
| EF0881 | NrdR family transcriptional regulator |  |  |  |  |  |  |  | 2.517 | 3.205 | transcriptional regulator |
| EF2696 | ADP-ribose diphosphatase | [nudF](http://www.ncbi.nlm.nih.gov/entrez/query.fcgi?cmd=search&db=gene&term=nudF) |  |  |  |  |  |  | 2.515 |  | purine/pyrimidine metabolism |
| EF2191 | dTDP-4-dehydrorhamnose reductase | [strL](http://www.ncbi.nlm.nih.gov/entrez/query.fcgi?cmd=search&db=gene&term=strL) |  |  | 3.376 | 3.737 |  |  | 2.51 |  | cell envelope |
| EF2915 | possible aminodeoxychorismate lyase |  |  |  | 2.929 | 2.142 |  |  | 2.5 | 2.932 | Central and Intermediate metabolism |
| EF1519 | P-ATPase superfamily cation transporter |  |  |  |  |  |  |  | 2.498 | 2.479 | transport and binding |
| OG1RF_11544 | transcriptional regulator |  |  |  |  |  |  |  | 2.466 |  | transcriptional regulator |
| EF2182 | ABC superfamily ATP binding cassette transporter, ABC protein |  |  |  | 4.232 |  |  |  | 2.398 | 3.921 | transport and binding |
| EF0257 | transcription-repair coupling factor | [mfd](http://www.ncbi.nlm.nih.gov/entrez/query.fcgi?cmd=search&db=gene&term=mfd) |  |  |  |  |  |  | 2.396 | 3.141 | DNA repair/recombination |
| EF2196 | family 2 glycosyl transferase |  |  |  |  |  |  |  | 2.383 | 4.726 | glycosyltransferase |
| EF1524 | RNA-binding protein |  |  |  | 4.481 | 3.795 |  |  | 2.372 | 3.465 | hypothetical |
| EF1260 | response regulator |  |  |  |  |  |  |  | 2.368 | 5.128 | Two component system |
| EF1543 | ferredoxin | [fer](http://www.ncbi.nlm.nih.gov/entrez/query.fcgi?cmd=search&db=gene&term=fer) |  |  |  |  |  |  | 2.326 | 2.528 | oxidative stress |
| EF2443 | phosphoglycerate mutase |  |  |  |  |  |  |  | 2.326 | 2.404 | energy metabolism |
| EF0988 | cell division protein MraZ |  |  |  |  |  |  |  | 2.325 | 3.09 | replication |
| EF1201 | protein of hypothetical function DUF1212 |  |  |  |  |  |  |  | 2.324 | 2.947 | hypothetical |
| EF1589 | acetyltransferase |  |  |  |  |  |  |  | 2.309 |  | hypothetical |
| EF1415 | glutamate dehydrogenase | [gdhA](http://www.ncbi.nlm.nih.gov/entrez/query.fcgi?cmd=search&db=gene&term=gdhA) |  |  |  |  |  |  | 2.305 | 2.925 | energy metabolism |
| EF0365 | acyl-ACP thioesterase |  |  |  | 3.006 | 2.481 |  |  | 2.294 | 3.647 | hypothetical |
| EF2192 | dTDP-glucose 4,6-dehydratase | [rfbB](http://www.ncbi.nlm.nih.gov/entrez/query.fcgi?cmd=search&db=gene&term=rfbB) |  |  |  |  |  |  | 2.293 | 4.499 | cell envelope |
| EF1402 | stimulator of FtsZ polymerization and component of cell-division Z-ring |  |  |  | 6.081 | 4.017 |  |  | 2.238 | 2.537 | replication |
| EF0465 | LytR family transcriptional regulator |  |  |  |  | 2.06 |  |  | 2.234 |  | transcriptional regulator |
| EF2189 | conserved hypothetical protein |  |  |  |  |  |  |  | 2.204 | 4.445 | hypothetical |
| EF3151 | M16 family metallopeptidase |  |  |  | 2.645 |  |  |  | 2.174 | 2.752 | hypothetical |
| EF1002 | cell division protein DivIVA | [divIVA](http://www.ncbi.nlm.nih.gov/entrez/query.fcgi?cmd=search&db=gene&term=divIVA) |  |  | 6.286 | 4.512 |  |  | 2.121 |  | replication |
| EF0763 | excinuclease ABC, subunit A | [uvrA](http://www.ncbi.nlm.nih.gov/entrez/query.fcgi?cmd=search&db=gene&term=uvrA) |  |  |  |  |  |  | 2.075 |  | DNA repair/recombination |
| EF0906 | conserved hypothetical protein |  |  |  |  |  |  |  | 2.069 |  | hypothetical |
| EF3324 | glutaconyl-CoA decarboxylase | [gcdB](http://www.ncbi.nlm.nih.gov/entrez/query.fcgi?cmd=search&db=gene&term=gcdB) |  |  | 0.37 |  |  |  | 0.492 |  | Central and Intermediate metabolism |
| EF3332 | ribonuclease P | [rnpA](http://www.ncbi.nlm.nih.gov/entrez/query.fcgi?cmd=search&db=gene&term=rnpA) |  |  |  |  |  |  | 0.489 | 0.411 | translation |
| EF0912 | ABC superfamily ATP binding cassette transporter, ABC protein |  |  |  |  |  |  |  | 0.489 | 0.319 | transport and binding |
| EF0913 | transposase |  |  |  |  |  |  |  | 0.489 |  | transposase |
| EF2152 | cobalt (Co2+) ABC superfamily ATP binding cassette transporter, membrane protein |  |  |  | 0.139 | 0.181 |  |  | 0.484 |  | transport and binding |
| EF1327 | 2-hydroxyglutaryl-CoA dehydratase activator |  |  |  |  | 2.362 |  |  | 0.482 |  | transcriptional regulator |
| EF0400 | permease |  |  |  |  |  |  |  | 0.478 |  | hypothetical |
| EF0096 | YheO domain protein |  |  |  |  |  |  |  | 0.471 |  | hypothetical |
| EF3169 | metallophosphoesterase |  |  |  | 0.168 | 0.217 |  |  | 0.463 | 0.421 | hypothetical |
| EF0017 | ABC superfamily ATP binding cassette transporter, ABC protein |  |  |  |  |  |  |  | 0.46 |  | transport and binding |
| EF1170 | transcription termination factor Rho | [rho](http://www.ncbi.nlm.nih.gov/entrez/query.fcgi?cmd=search&db=gene&term=rho) |  |  |  |  |  |  | 0.46 |  | transcriptional regulator |
| EF1246 | integral membrane protein |  |  |  |  |  |  |  | 0.46 |  | cell envelope |
| EF3118 | ribulose-phosphate 3-epimerase | [rpe](http://www.ncbi.nlm.nih.gov/entrez/query.fcgi?cmd=search&db=gene&term=rpe) |  |  |  |  |  |  | 0.46 |  | energy metabolism |
| EF0728 | TrmA family tRNA (uracil-5-)-methyltransferase |  |  |  |  |  |  |  | 0.458 |  | translation |
| EF0891 | aspartate aminotransferase |  |  |  |  |  |  |  | 0.456 |  | replication |
| EF1183 | aspartate-semialdehyde dehydrogenase | [asd](http://www.ncbi.nlm.nih.gov/entrez/query.fcgi?cmd=search&db=gene&term=asd) |  |  |  |  |  |  | 0.454 |  | Central and Intermediate metabolism |
| [EF3294](http://www.ncbi.nlm.nih.gov/sites/entrez?db=gene&cmd=search&term=1202137&RID=6CTW16V501R&log$=geneexplicitprot&blast_rank=13) | conserved hypothetical protein |  |  |  |  |  |  |  | 0.451 |  | hypothetical |
| EF0001 | chromosomal replication initiator protein DnaA | [dnaA](http://www.ncbi.nlm.nih.gov/entrez/query.fcgi?cmd=search&db=gene&term=dnaA) |  |  |  |  |  |  | 0.45 |  | replication |
| EF2934 | thiamine biosynthesis protein | [thiI](http://www.ncbi.nlm.nih.gov/entrez/query.fcgi?cmd=search&db=gene&term=thiI) |  |  |  |  |  |  | 0.446 |  | cofactor biosynthesis |
| EF2208 | phenazine biosynthesis protein |  |  |  |  |  |  |  | 0.445 |  | virulence factors |
| EF2735 | exodeoxyribonuclease | [exoA](http://www.ncbi.nlm.nih.gov/entrez/query.fcgi?cmd=search&db=gene&term=exoA) |  |  | 0.269 | 0.316 |  |  | 0.443 |  | DNA repair |
| EF0196 | recombinase |  |  |  |  |  |  |  | 0.443 |  | DNA repair/recombination |
| EF1494 | proton (H+) or sodium (Na+) translocating V family ATPase (V-ATPase), subunit K |  |  |  |  |  |  |  | 0.442 |  | transport and binding |
| EF1497 | V family ATP synthase subunit F |  |  |  |  |  |  |  | 0.441 |  | transport and binding |
| EF3297 | protein of hypothetical function DUF951 |  |  |  |  |  |  |  | 0.436 | 0.446 | hypothetical |
| EF0270 | protein-N(pi)-phosphohistidine--sugar phosphotransferase | [bglP-2](http://www.ncbi.nlm.nih.gov/entrez/query.fcgi?cmd=search&db=gene&term=bglP-2) | 0.469 |  | 0.374 | 0.377 |  |  | 0.434 |  | energy metabolism |
| EF0778 | conserved hypothetical protein |  |  |  |  |  |  |  | 0.428 |  | hypothetical |
| EF3296 | GTP-binding translation factor YchF |  |  |  |  |  |  |  | 0.427 | 0.426 | hypothetical |
| EF1559 | thioesterase/dihydrolipoamide acyltransferase |  |  |  |  |  |  |  | 0.427 |  | central and intermediate metabolism |
| EF1527 | GTP-binding protein |  |  |  |  | 0.234 |  |  | 0.421 | 0.432 | hypothetical |
| EF2158 | pyruvate synthase |  |  |  |  |  |  |  | 0.42 |  | energy metabolism |
| EF0452 | acetate--CoA ligase |  |  |  |  |  |  |  | 0.419 |  | cofactor biosynthesis |
| EF3298 | chromosome partitioning protein, DNA-binding protein |  |  |  |  |  |  |  | 0.419 |  | replication |
| EF3329 | response regulator |  |  |  | 0.371 | 0.411 |  |  | 0.416 | 0.414 | Two component system |
| EF2153 | ABC superfamily ATP binding cassette transporter, ABC protein |  |  |  | 0.145 | 0.327 |  |  | 0.416 |  | transport and binding |
| EF1635 | possible alcohol dehydrogenase |  |  |  |  |  |  |  | 0.413 |  | energy metabolism |
| EF2058 | ABC superfamily ATP binding cassette transporter, ABC/membrane protein |  |  |  | 0.2 |  |  |  | 0.41 | 0.447 | transport and binding |
| EF0394 | M23B subfamily peptidase |  |  | 0.369 |  | 3.405 |  |  | 0.403 |  | peptidase/protease |
| OG1RF_10404 | CRISPR associated protein |  |  |  |  |  |  |  | 0.397 |  | phage related |
| EF0896 | queuine tRNA-ribosyltransferase | [tgt](http://www.ncbi.nlm.nih.gov/entrez/query.fcgi?cmd=search&db=gene&term=tgt) |  |  |  |  |  |  | 0.391 | 0.398 | translation |
| EF3293 | inositol-5-monophosphate dehydrogenase | [guaB](http://www.ncbi.nlm.nih.gov/entrez/query.fcgi?cmd=search&db=gene&term=guaB) |  |  |  |  |  |  | 0.391 |  | purine/pyrimidine metabolism |
| EF0014 | adenylosuccinate synthetase | [purA](http://www.ncbi.nlm.nih.gov/entrez/query.fcgi?cmd=search&db=gene&term=purA) |  |  |  |  |  |  | 0.39 | 0.303 | purine/pyirmidine metabolism |
| EF2723A | M55 family D-aminopeptidase |  |  |  |  |  |  |  | 0.389 | 0.183 | cell envelope |
| EF2650 | spermidine/putrescine ABC superfamily ATP binding cassette transporter, membrane protein |  |  |  | 0.209 |  |  |  | 0.385 |  | transport and binding |
| EF0751 | WxL domain surface protein |  |  |  |  |  |  |  | 0.373 |  | cell envelope |
| EF1561 | shikimate 5-dehydrogenase | [aroE](http://www.ncbi.nlm.nih.gov/entrez/query.fcgi?cmd=search&db=gene&term=aroE) |  |  |  |  |  |  | 0.372 |  | central and intermediate metabolism |
| EF1293 | endolysin | [ply-1](http://www.ncbi.nlm.nih.gov/entrez/query.fcgi?cmd=search&db=gene&term=ply-1) |  |  |  |  |  |  | 0.371 | 0.331 | cell envelope |
| EF2154 | membrane protein |  |  |  | 0.183 | 0.305 |  |  | 0.371 |  | cell envelope |
| EF0175 | cytidine deaminase | [cdd](http://www.ncbi.nlm.nih.gov/entrez/query.fcgi?cmd=search&db=gene&term=cdd) |  |  |  |  |  |  | 0.37 |  | purine/pyrimidine metabolism |
| EF0174 | deoxyribose-phosphate aldolase | [deoC](http://www.ncbi.nlm.nih.gov/entrez/query.fcgi?cmd=search&db=gene&term=deoC) |  |  |  |  |  |  | 0.368 |  | purine/pyrimidine metabolism |
| EF0700 | hemolysin |  |  |  |  |  |  |  | 0.364 |  | virulence factors |
| EF0248 | conserved hypothetical protein |  |  |  | 0.177 | 0.229 |  |  | 0.359 |  | hypothetical |
| EF0631 | cardiolipin synthetase |  |  |  |  |  |  |  | 0.347 | 0.29 | cell envelope |
| EF3254 | 1,4-dihydroxy-2-naphthoate octaprenyltransferase | [ubiA-2](http://www.ncbi.nlm.nih.gov/entrez/query.fcgi?cmd=search&db=gene&term=ubiA-2) |  |  |  | 0.492 |  |  | 0.346 |  | cofactor biosynthesis |
| EF0911 | ABC superfamily ATP binding cassette transporter, ABC protein |  |  |  |  |  |  |  | 0.343 | 0.399 | transport and binding |
| EF3004 | SulP family sulfate permease |  |  |  |  | 0.295 |  |  | 0.339 |  | transport and binding |
| EF1290 | structural protein |  |  |  |  |  |  |  | 0.338 |  | cell envelope |
| EF2441 | possible phosphate transport regulator |  |  |  |  |  |  |  | 0.33 |  | transport and binding |
| EF1408 | ABC superfamily ATP binding cassette transporter, ABC protein |  |  |  |  |  |  |  | 0.329 | 0.318 | transport and binding |
| EF2601 | [acyl-carrier-protein] phosphodiesterase |  |  |  |  |  |  |  | 0.327 | 0.396 | energy metbolism |
| EF0176 | ABC superfamily ATP binding cassette transporter, binding protein |  |  |  |  |  |  |  | 0.323 | 0.268 | transport and binding |
| EF3300 | glucose-inhibited division protein B | [gidB](http://www.ncbi.nlm.nih.gov/entrez/query.fcgi?cmd=search&db=gene&term=gidB) |  |  |  |  |  |  | 0.322 | 0.331 | replication |
| EF1409 | ABC superfamily ATP binding cassette transporter, membrane protein |  |  |  | 0.262 |  |  |  | 0.318 | 0.359 | transport and binding |
| EF3299 | chromosome partitioning ATPase |  |  |  |  |  |  |  | 0.314 |  | replication |
| EF2851 | conserved hypothetical protein |  |  |  |  |  |  |  | 0.313 |  | hypothetical |
| EF2876 | acetyl-CoA carboxylase subunit beta | [accD](http://www.ncbi.nlm.nih.gov/entrez/query.fcgi?cmd=search&db=gene&term=accD) |  |  |  |  |  |  | 0.309 |  | cofacror metabolism |
| EF0074 | CRP family transcriptional regulator |  |  |  |  |  |  |  | 0.306 | 0.383 | transcriptional regulator |
| EF1596 | lipoprotein |  |  |  |  |  |  |  | 0.306 | 0.163 | cell envelope |
| EF3069 | FNT family formate-nitrite transporter |  |  |  | 0.225 | 0.268 |  |  | 0.303 | 0.375 | transport and binding |
| EF3255 | thiamine biosynthesis lipoprotein |  |  |  | 0.454 | 0.469 |  |  | 0.301 |  | cell envelope |
| EF2878 | (3R)-hydroxymyristoyl-ACP dehydratase | [fabZ](http://www.ncbi.nlm.nih.gov/entrez/query.fcgi?cmd=search&db=gene&term=fabZ) |  |  |  |  |  |  | 0.3 |  | cell envelope |
| EF0872 | KUP family potassium (K+) uptake permease |  |  |  | 0.23 | 0.196 |  |  | 0.292 |  | transport and binding |
| EF0369 | haloacid dehalogenase (HAD) superfamily hydrolase |  |  |  |  |  |  |  | 0.288 | 0.213 | hypothetical |
| EF2621 | acetyltransferase |  |  |  | 4.649 |  |  |  | 0.282 | 0.203 | hypothetical |
| EF0024 | ManO family protein |  |  |  | 0.431 |  |  |  | 0.278 | 0.339 | energy metabolism |
| EF2440 | YdjC family protein |  |  |  |  |  |  |  | 0.269 | 0.141 | hypothetical |
| EF2649 | spermidine/putrescine ABC superfamily ATP binding cassette transporter, binding protein |  |  |  | 0.289 |  |  |  | 0.269 |  | transport and binding |
| EF0108 | C4-dicarboxylate anaerobic carrier |  |  |  | 0.055 | 0.202 |  |  | 0.262 |  | hypothetical |
| EF2725 | oligopeptide ABC superfamily ATP binding cassette transporter, binding protein |  |  |  |  |  |  |  | 0.255 | 0.095 | transport and binding |
| EF0022 | PTS family mannose/fructose/sorbose porter component IID |  |  |  | 0.25 |  |  |  | 0.255 |  | energy metabolism |
| EF0399 | protein of hypothetical function DUF969 |  |  |  |  |  |  |  | 0.255 |  | hypothetical |
| EF2882 | acyl-carrier-protein S-malonyltransferase | [fabD](http://www.ncbi.nlm.nih.gov/entrez/query.fcgi?cmd=search&db=gene&term=fabD) |  |  |  |  |  |  | 0.248 |  | cell envelope |
| EF3257 | NADH dehydrogenase |  |  |  | 0.141 | 0.307 |  |  | 0.245 |  | oxidative stress |
| EF1634 | ethanolamine utilization protein EutS |  |  |  |  |  |  |  | 0.245 |  | energy metabolism |
| EF2883 | enoyl-(acyl-carrier-protein) reductase II | [fabK](http://www.ncbi.nlm.nih.gov/entrez/query.fcgi?cmd=search&db=gene&term=fabK) |  |  |  |  |  |  | 0.244 |  | cell envelope |
| EF1400 | ABC superfamily ATP binding cassette transporter ATPase |  |  |  |  |  |  |  | 0.239 |  | transport and binding |
| OG1RF_10876 | hypothetical protein |  |  |  |  |  |  |  | 0.238 | 0.275 | hypothetical |
| [EF3328](http://www.ncbi.nlm.nih.gov/sites/entrez?db=gene&cmd=search&term=1202171&RID=6CWBNG9P01S&log$=geneexplicitprot&blast_rank=1) | GntR family transcriptional regulator |  |  |  |  |  |  |  | 0.236 |  | transcriptional regulator |
| EF1779 | phosphoribosylglycinamide formyltransferase | [purN](http://www.ncbi.nlm.nih.gov/entrez/query.fcgi?cmd=search&db=gene&term=purN) |  |  |  |  |  |  | 0.212 | 0.207 | purine/pyrimidine mebolism |
| EF2442 | PiT family inorganic phosphate transporter |  |  |  | 0.263 |  |  |  | 0.212 |  | transport and binding |
| OG1RF_11700 | hypothetical protein |  |  |  |  |  |  |  | 0.212 |  | hypothetical |
| EF3211 | protein-N(pi)-phosphohistidine--sugar phosphotransferase | ulaB |  |  | 0.172 |  |  |  | 0.198 | 0.172 | energy metabolism |
| EF0019 | PTS family mannose/fructose/sorbose porter component IIB |  |  |  | 0.153 |  |  |  | 0.197 |  | energy metabolism |
| EF1097 | conserved hypothetical protein |  |  |  |  |  |  |  | 0.189 | 0.244 | hypothetical |
| EF3212 | PTS family fructose/mannitol (fru) porter component IIC |  |  |  | 0.167 | 0.27 |  |  | 0.187 |  | energy metabolism |
| EF3210 | PTS family fructose/mannitol (fru) porter component IIA |  |  |  |  | 0.333 |  |  | 0.181 | 0.129 | energy metabolism |
| OG1RF_10796 | hypothetical protein |  |  |  |  |  |  |  | 0.177 | 0.234 | hypothetical |
| EF0020 | protein-N(pi)-phosphohistidine--sugar phosphotransferase | [manX](http://www.ncbi.nlm.nih.gov/entrez/query.fcgi?cmd=search&db=gene&term=manX) |  |  | 0.194 |  |  |  | 0.167 |  | energy metabolism |
| EF2364 | NCS2 family nucleobase:cation symporter-2 |  |  |  |  | 0.462 |  |  | 0.164 | 0.099 | purine metabolism |
| EF0720 | CPA2 family monovalent cation:proton (H+) antiporter-2 |  |  |  | 0.115 | 0.183 |  |  | 0.159 | 0.289 | transport and binding |
| EF2365 | xanthine phosphoribosyltransferase | [xpt](http://www.ncbi.nlm.nih.gov/entrez/query.fcgi?cmd=search&db=gene&term=xpt) |  |  |  |  |  |  | 0.157 | 0.092 | purine/pyrimidine metabolism |
| EF1818 | possible metalloprotease | [nprE](http://www.ncbi.nlm.nih.gov/entrez/query.fcgi?cmd=search&db=gene&term=nprE) |  |  |  |  |  |  | 0.154 | 0.333 | peptidase/protease |
| EF1041 | NCS2 family nucleobase:cation symporter-2 |  |  |  | 0.25 | 0.25 |  |  | 0.151 | 0.165 | transport and binding |
| OG1RF_10797 | possible DNA repair protein RecN | [recN](http://www.ncbi.nlm.nih.gov/entrez/query.fcgi?cmd=search&db=gene&term=recN) |  |  |  |  |  |  | 0.14 |  | DNA repair/recombination |
| OG1RF_10795 | hypothetical protein |  |  |  |  |  |  |  | 0.126 |  | hypothetical |
| EF2011 | conserved hypothetical protein |  |  |  |  | 0.181 |  |  | 0.124 |  | hypothetical |
| EF1220 | ABC superfamily ATP binding cassette transporter, ATPase |  |  |  |  |  |  |  | 0.121 |  | transport and binding |
| EF3173 | mucin-associated surface protein (MASP) |  |  |  | 0.196 |  |  |  | 0.11 | 0.12 | cell envelope |
| EF1223 | possible S-adenosylhomocysteine deaminase | [mtaD1](http://www.ncbi.nlm.nih.gov/entrez/query.fcgi?cmd=search&db=gene&term=mtaD1) |  |  |  | 0.247 |  |  | 0.104 |  | hypothetical |
| EF1218 | spermidine/putrescine ABC superfamily ATP binding cassette transporter, membrane protein |  |  |  |  |  |  |  | 0.096 |  | transport and binding |
| EF1222 | adenine deaminase | [ade](http://www.ncbi.nlm.nih.gov/entrez/query.fcgi?cmd=search&db=gene&term=ade) |  |  |  | 0.113 |  |  | 0.092 | 0.06 | aa metabolism |
| EF2556 | succinate dehydrogenase |  |  |  |  |  |  |  | 0.069 | 0.177 | energy metabolism |
| EF1054 | ABC superfamily ATP binding cassette transporter, membrane protein |  |  |  |  | 9.603 |  |  |  | 54.347 | transport and binding |
| EF3205 | conserved hypothetical protein |  |  |  |  | 2.811 |  |  |  | 9.262 | hypothetical |
| EF3206 | ABC superfamily ATP binding cassette transporter, binding protein |  |  |  |  |  |  |  |  | 8.046 | transport and binding |
| OG1RF_11452 | ABC superfamily ATP binding cassette transporter, binding protein |  |  |  |  |  |  |  |  | 7.806 | transport and binding |
| EF1305 | coproporphyrinogen dehydrogenase | [hemN](http://www.ncbi.nlm.nih.gov/entrez/query.fcgi?cmd=search&db=gene&term=hemN) |  |  |  |  |  |  |  | 7.611 | Heme |
| EF1226 | possible NAD(P)H dehydrogenase (quinone) |  |  |  |  | 2.599 |  |  |  | 7.155 | hypothetical |
| EF1656 | conserved hypothetical protein |  |  |  |  |  |  |  |  | 6.955 | transcriptional regulator |
| EF1225 | possible thiamine biosynthesis lipoprotein |  |  |  |  | 2.146 |  |  |  | 6.887 | cofactor biosynthesis |
| EF227 | NADPH-dependent FMN reductase domain protein |  |  |  |  |  |  |  |  | 6.763 | hypothetical |
| EF1672 | peptide ABC superfamily ATP binding cassette transporter permease |  | 4.75 |  |  |  |  |  |  | 6.449 | transport and binding |
| EF1053 | taurine-transporting ATPase |  |  |  |  |  |  |  |  | 6.022 | transport and binding |
| EF0692 | RpiR family transcriptional regulator |  |  |  |  |  |  |  |  | 5.498 | transcriptional regulator |
| EF3136 | PTS family mannose/fructose/sorbose porter component IIA |  |  |  |  |  |  |  |  | 5.478 | energy metabolism |
| EF1707 | alpha-mannosidase |  |  |  |  | 2.979 |  |  |  | 5.393 | energy metabolism |
| EF0879 | hypothetical protein |  |  |  |  |  |  |  |  | 5.203 | hypothetical |
| EF1127 | ascorbate-specific PTS system enzyme IIC | [ulaA-1](http://www.ncbi.nlm.nih.gov/entrez/query.fcgi?cmd=search&db=gene&term=ulaA-1) |  |  |  |  |  |  |  | 4.377 | energy metabolism |
| EF0447 | possible isochorismate synthase | [menF](http://www.ncbi.nlm.nih.gov/entrez/query.fcgi?cmd=search&db=gene&term=menF) |  |  |  |  |  |  |  | 3.936 | Central and Intermediate metabolism |
| EF0367 | Cof family protein |  |  |  | 3.219 | 2.798 |  |  |  | 3.27 | hypothetical |
| OG1RF_10622 | hypothetical protein |  |  |  |  |  |  |  |  | 3.266 | hypothetical |
| EF1546 | LysM domain protein |  |  |  | 4.829 | 2.696 |  |  |  | 3.258 | hypothetical |
| EF0739 | NMN family nicotinamide monnucleotide uptake permease |  |  |  |  |  |  |  |  | 3.18 | cofactor biosynthesis |
| EF1552 | conserved hypothetical protein |  |  |  |  |  |  |  |  | 3.019 | hypothetical |
| EF2930 | integral membrane protein |  |  |  |  |  |  |  |  | 2.983 | cell envelope |
| EF2157 | protein of hypothetical function DUF147 |  |  |  |  |  |  |  |  | 2.863 | hypothetical |
| EF0613 | hypothetical protein |  |  |  |  |  |  |  |  | 2.837 | hypothetical |
| EF2899 | thioredoxin-disulfide reductase |  |  |  |  | 0.244 |  |  |  | 2.767 | oxidative stress |
| EF1793 | branched-chain amino acid aminotransferase | [ilvE](http://www.ncbi.nlm.nih.gov/entrez/query.fcgi?cmd=search&db=gene&term=ilvE) |  |  |  |  |  |  |  | 2.731 | aa metabolism |
| EF1157 | M20/M25/M40 family peptidase |  |  |  |  |  |  |  |  | 2.721 | peptidase/protease |
| EF0989 | S-adenosyl-methyltransferase MraW | [mraW](http://www.ncbi.nlm.nih.gov/entrez/query.fcgi?cmd=search&db=gene&term=mraW) |  |  |  |  |  |  |  | 2.71 | cell envelope |
| EF2181 | group 2 family glycosyl transferase |  |  |  |  |  |  |  |  | 2.673 | glycosyltransferase |
| EF2424 | possible pyrroline-5-carboxylate reductase |  |  |  |  |  |  |  |  | 2.607 | aa metabolism |
| EF2180 | family 2 glycosyl transferase |  |  |  |  |  |  |  |  | 2.574 | glycosyltransferase |
| EF1156 | transcriptional regulator |  |  |  |  |  |  |  |  | 2.466 | transcriptional regulator |
| EF2500 | glycine cleavage system protein H |  |  |  |  |  |  |  |  | 2.413 | aa metabolism |
| EF0669 | MOP superfamily multidrug/oligosaccharidyl-lipid/polysaccharide flippase transporter |  |  |  |  |  |  |  |  | 2.373 | energy metabolism |
| EF0766 | ATP-binding protein |  |  |  | 2.65 |  |  |  |  | 2.326 | hypothetical |
| EF2178 | conserved hypothetical protein |  |  |  |  |  |  |  |  | 2.268 | hypothetical |
| EF0992 | phospho-N-acetylmuramoyl-pentapeptide- transferase | [mraY](http://www.ncbi.nlm.nih.gov/entrez/query.fcgi?cmd=search&db=gene&term=mraY) |  |  |  |  |  |  |  | 2.095 | cell envelope |
| EF2061 | cytochrome d ubiquinol oxidase, subunit I | [cydA](http://www.ncbi.nlm.nih.gov/entrez/query.fcgi?cmd=search&db=gene&term=cydA) |  |  | 0.148 | 0.432 |  |  |  | 0.486 | oxidative stress |
| OG1RF_12484 | hypothetical protein |  |  |  | 0.364 |  |  |  |  | 0.474 | hypothetical |
| EF2207 | leucine-rich protein |  |  |  |  |  |  |  |  | 0.451 | hypothetical |
| EF2680 | ABC superfamily ATP binding cassette transporter, membrane protein | [lmrA](http://www.ncbi.nlm.nih.gov/entrez/query.fcgi?cmd=search&db=gene&term=lmrA) |  |  |  |  |  |  |  | 0.443 | transport and binding |
| EF0066 | Holliday junction DNA helicase RuvA | [ruvA](http://www.ncbi.nlm.nih.gov/entrez/query.fcgi?cmd=search&db=gene&term=ruvA) |  |  |  |  |  |  |  | 0.436 | DNA repair/recombination |
| EF0702 | conserved hypothetical protein |  |  |  |  |  |  |  |  | 0.429 | hypothetical |
| EF1288 | conserved hypothetical protein |  |  |  |  |  |  |  |  | 0.429 | hypothetical |
| EF3127 | guanylate kinase | [gmk](http://www.ncbi.nlm.nih.gov/entrez/query.fcgi?cmd=search&db=gene&term=gmk) |  |  |  |  |  |  |  | 0.422 | purine/pyrimidine metabolism |
| EF3126 | DNA-directed RNA polymerase subunit omega | [rpoZ](http://www.ncbi.nlm.nih.gov/entrez/query.fcgi?cmd=search&db=gene&term=rpoZ) |  |  |  |  |  |  |  | 0.413 | transcription |
| EF0016 | YitS spore protein |  |  |  |  |  |  |  |  | 0.412 | hypothetical |
| EF0676 | arginine repressor | [argR](http://www.ncbi.nlm.nih.gov/entrez/query.fcgi?cmd=search&db=gene&term=argR) |  |  |  |  |  |  |  | 0.409 | transcriptional regulator |
| EF1905 | conserved hypothetical protein |  |  | 0.094 |  |  |  |  |  | 0.386 | hypothetical |
| EF0003 | RNA-binding S4 protein |  |  |  |  |  |  |  |  | 0.385 | hypothetical |
| EF2056 | 1,4-dihydroxy-2-naphthoate octaprenyltransferase | [ubiA-1](http://www.ncbi.nlm.nih.gov/entrez/query.fcgi?cmd=search&db=gene&term=ubiA-1) |  |  |  |  |  |  |  | 0.383 | cofactor biosynthesis |
| EF2720 | ABC superfamily ATP binding cassette transporter, ABC protein Lsa |  |  |  |  |  |  |  |  | 0.366 | transport and binding |
| EF1991 | cold shock protein CspC | [cspC](http://www.ncbi.nlm.nih.gov/entrez/query.fcgi?cmd=search&db=gene&term=cspC) |  |  |  |  |  |  |  | 0.363 | stress |
| OG1RF_10129 | possible membrane protein |  |  |  |  |  |  |  |  | 0.36 | cell envelope |
| EF1089 | conserved hypothetical protein |  |  |  | 0.374 |  |  |  |  | 0.341 | hypothetical |
| EF2377 | APC family amino acid-polyamine-organocation transporter |  |  |  |  |  |  |  |  | 0.339 | aa metabolism |
| EF1146 | DNA-directed RNA polymerase subunit delta |  |  |  |  |  |  |  |  | 0.338 | transcription |
| EF0929 | APC family amino acid-polyamine-organocation transporter |  |  |  | 0.182 | 0.367 |  |  |  | 0.332 | transport and binding |
| EF2136 | conserved hypothetical protein |  |  |  | 0.204 | 0.254 |  |  |  | 0.327 | hypothetical |
| EF0178 | possible monosaccharide-transporting ATPase | [rbsA3](http://www.ncbi.nlm.nih.gov/entrez/query.fcgi?cmd=search&db=gene&term=rbsA3) |  |  |  |  |  |  |  | 0.324 | transport and binding |
| EF0855 | conserved hypothetical protein |  |  |  |  |  |  |  |  | 0.321 | hypothetical |
| EF1407 | conserved hypothetical protein |  |  |  |  |  |  |  |  | 0.32 | hypothetical |
| EF0907 | oligopeptide ABC superfamily ATP binding cassette transporter, binding protein |  |  |  |  |  |  |  |  | 0.319 | transport and binding |
| EF2175 | conserved hypothetical protein |  |  |  |  |  |  |  |  | 0.314 | hypotetical |
| EF1292 | holin |  |  |  |  |  |  |  |  | 0.303 | cell envelope |
| EF1925 | conserved hypothetical protein |  |  |  |  |  |  |  |  | 0.291 | hypothetical |
| EF0027 | phosphosugar-binding transcriptional regulator |  |  |  | 0.208 |  |  |  |  | 0.283 | transcriptional regulator |
| EF0241 | protein of hypothetical function DUF74 |  |  |  |  |  |  |  |  | 0.277 | hypothetical |
| EF1191 | DegV family protein |  |  |  |  |  |  |  |  | 0.265 | hypothetical |
| EF3213 | PTS family porter component II |  |  |  |  |  |  |  |  | 0.264 | energy metabolism |
| EF1177 | conserved hypothetical protein |  |  |  |  |  |  |  |  | 0.25 | hypothetical |
| EF1033 | 6-aminohexanoate-cyclic-dimer hydrolase |  |  |  |  |  |  |  |  | 0.249 | Central and Intermediate metabolism |
| EF0402 | Na+/H+ antiporter | [nhaC-1](http://www.ncbi.nlm.nih.gov/entrez/query.fcgi?cmd=search&db=gene&term=nhaC-1) |  |  | 0.129 | 0.243 |  |  |  | 0.239 | transport and binding |
| OG1RF_10596 | hypothetical protein |  |  |  |  |  |  |  |  | 0.235 | hypothetical |
| EF0177 | ABC superfamily ATP binding cassette transporter, binding protein |  |  |  |  |  |  |  |  | 0.214 | transport and binding |
| EF2622 | conserved hypothetical protein |  |  |  | 6.448 | 5.413 |  |  |  | 0.168 | hypothetical |
| OG1RF_11817 | hypothetical protein |  |  |  |  |  |  |  |  | 0.156 | hypothetical |
| EF2724 | possible glutamyl aminopeptidase |  |  |  |  |  |  |  |  | 0.146 | peptidase/protease |
| OG1RF_12376 | possible response regulator |  |  |  |  |  |  |  |  | 0.13 | Two component system |
| EF3060 | secreted lipase |  |  |  |  | 10.818 |  |  |  |  | cell envelope |
| OG1RF_10075 | hypothetical protein |  |  |  |  | 7.45 |  |  |  |  | hypothetical |
| EF0681 | protein of hypothetical function DUF964 |  | 3.092 |  | 11.517 | 7.107 |  |  |  |  | hypothetical |
| EF1933 | conserved hypothetical protein |  | 4.419 |  | 7.501 | 6.875 |  |  |  |  | hypothetical |
| EF1227 | NADPH-dependent FMN reductase domain protein |  |  |  |  | 6.763 |  |  |  |  | hypothetical |
| EF1215 | conserved hypothetical protein |  |  |  | 3.852 | 6.04 |  |  |  |  | hypothetical |
| EF1673 | ABC superfamily ATP binding cassette transporter ABC protein | [phnC-3](http://www.ncbi.nlm.nih.gov/entrez/query.fcgi?cmd=search&db=gene&term=phnC-3) |  |  |  | 5.61 |  |  |  |  | transport and binding |
| EF0468 | membrane protein LemA family protein |  |  |  | 9.749 | 5.252 |  |  |  |  | cell envelope |
| OG1RF_10977 | cro/CI family TPR domain transcriptional regulator |  |  |  |  | 5.16 |  |  |  |  | transcriptional regulator |
| EF1022 | conserved hypothetical protein |  |  |  | 6.52 | 5.147 |  |  |  |  | hypothetical |
| OG1RF_12236 | hypothetical protein |  |  |  | 3.178 | 4.985 |  |  |  |  | hypothetical |
| EF2885 | 3-oxoacyl-(acyl carrier protein) synthase III | [fabH](http://www.ncbi.nlm.nih.gov/entrez/query.fcgi?cmd=search&db=gene&term=fabH) |  |  | 7.676 | 4.937 |  |  |  |  | cell envelope |
| [OG1RF_10628](http://www.ncbi.nlm.nih.gov/entrez/viewer.fcgi?db=protein&val=327534481) | isopentenyl pyrophosphate isomerase | [fni](http://www.ncbi.nlm.nih.gov/entrez/query.fcgi?cmd=search&db=gene&term=fni) |  |  | 2.025 | 4.9 |  |  |  |  | Central and Intermediate metabolism |
| OG1RF_12163 | response regulator VanRG | vanRG |  |  | 10.518 | 4.872 |  |  |  |  | Two component system |
| OG1RF_11535 | WxL domain surface protein |  |  |  |  | 4.85 |  |  |  |  | cell envelope |
| EF1586 | NADH oxidase | [nox](http://www.ncbi.nlm.nih.gov/entrez/query.fcgi?cmd=search&db=gene&term=nox) |  |  |  | 4.759 |  |  |  |  | oxidative stress |
| EF1936 | conserved hypothetical protein |  |  |  | 6.267 | 4.743 |  |  |  |  | hypothetical |
| EF1193 | DNA-binding response regulator |  |  |  | 4.648 | 4.735 |  |  |  |  | Two component system |
| EF2856 | ribosomal protein L33 | [rpmG-3](http://www.ncbi.nlm.nih.gov/entrez/query.fcgi?cmd=search&db=gene&term=rpmG-3) |  |  | 5.287 | 4.594 |  |  |  |  | ribosomal |
| EF2929A | conserved hypothetical protein |  |  |  |  | 4.575 |  |  |  |  | hypothetical |
| EF1250 | conserved hypothetical protein |  |  |  | 3.556 | 4.286 |  |  |  |  | hypothetical |
| EF1315 | conserved hypothetical protein |  |  |  | 5.031 | 4.258 |  |  |  |  | hypothetical |
| EF1165 | nucleotide-binding protein |  |  |  | 6.172 | 4.168 |  |  |  |  | hypothetical |
| EF1025 | transcriptional regulator |  |  |  |  | 4.146 |  |  |  |  | transcriptional regulator |
| EF3018 | conserved hypothetical protein |  |  |  |  | 4.128 |  |  |  |  | hypothetical |
| OG1RF_10820 | possible LytR family response regulator |  |  |  | 7.068 | 3.968 |  |  |  |  | Two component system |
| OG1RF_11924 | cell wall surface anchor protein |  |  |  |  | 3.89 |  |  |  |  | cell envelope |
| OG1RF_11718 | hypothetical protein |  |  |  |  | 3.889 |  |  |  |  | hypothetical |
| EF1525 | Fur family transcriptional regulator Fur |  |  |  | 4.569 | 3.866 |  |  |  |  | transcriptional regulator |
| EF1324 | conserved hypothetical protein |  |  |  | 4.34 | 3.85 |  |  |  |  | hypothetical |
| OG1RF_10819 | possible membrane protein |  |  |  | 4.297 | 3.745 |  |  |  |  | cell envelope |
| EF0990 | cell division protein |  |  |  | 5.551 | 3.708 |  |  |  |  | replication |
| EF1764 | ribosome-associated inhibitor protein Y | [yfiA](http://www.ncbi.nlm.nih.gov/entrez/query.fcgi?cmd=search&db=gene&term=yfiA) |  |  |  | 3.702 |  |  |  |  | translation |
| OG1RF_10811 | possible collagen adhesion protein |  |  |  |  | 3.699 |  |  |  |  | cell envelope |
| OG1RF_10809 | hypothetical protein |  |  |  |  | 3.639 |  |  |  |  | hypothetical |
| EF0417 | periplasmic component of efflux system |  |  |  | 3.77 | 3.597 |  |  |  |  | transport and binding |
| EF1692 | conserved hypothetical protein |  |  |  | 4.325 | 3.57 |  |  |  |  | hypothetical |
| EF2884 | acyl carrier protein | [acpP1](http://www.ncbi.nlm.nih.gov/entrez/query.fcgi?cmd=search&db=gene&term=acpP1) |  |  | 5.553 | 3.533 |  |  |  |  | cell envelope |
| EF2296 | conserved hypothetical protein |  | 3.329 |  |  | 3.515 |  |  |  |  | antibiotic resistance |
| OG1RF_11534 | possible membrane protein |  |  |  |  | 3.513 |  |  |  |  | cell envelope |
| EF2160 | glutamate--ammonia ligase repressor | [glnR](http://www.ncbi.nlm.nih.gov/entrez/query.fcgi?cmd=search&db=gene&term=glnR) |  |  | 0.171 | 3.442 |  |  |  |  | transcriptonal regulator |
| OG1RF_11100 | hypothetical protein |  |  | 3.344 | 7.442 | 3.415 |  |  |  |  | hypothetical |
| EF0685 | peptidylprolyl isomerase | [prsA](http://www.ncbi.nlm.nih.gov/entrez/query.fcgi?cmd=search&db=gene&term=prsA) |  |  | 7.281 | 3.415 |  |  |  |  | protein folding |
| EF1059 | conserved hypothetical protein |  |  |  |  | 3.376 |  |  |  |  | hypothetical |
| EF1316 | transcriptional regulator |  |  |  | 4.602 | 3.366 |  |  |  |  | transcriptional regulator |
| EF0979 | exodeoxyribonuclease VII large subunit | [xseA](http://www.ncbi.nlm.nih.gov/entrez/query.fcgi?cmd=search&db=gene&term=xseA) |  |  |  | 3.342 |  |  |  |  | DNA repair/recombination |
| EF2890 | glycosyltransferase |  |  |  | 3.899 | 3.313 |  |  |  |  | glycosyltransferase |
| EF2826 | conserved hypothetical protein |  |  |  |  | 3.312 |  |  |  |  | hypothetical |
| EF2999 | allantoinase | [allB](http://www.ncbi.nlm.nih.gov/entrez/query.fcgi?cmd=search&db=gene&term=allB) |  |  |  | 3.311 |  |  |  |  | Central and Intermediate metabolism |
| EF1251 | conserved hypothetical protein |  |  |  | 4.301 | 3.263 |  |  |  |  | hypothetical |
| OG1RF_10826 | hypothetical protein |  |  |  |  | 3.255 |  |  |  |  | hypothetical |
| EF1599 | transcriptional regulator |  |  |  |  | 3.248 |  |  |  |  | transcriptional regulator |
| EF1761 | cell division ATP-binding protein FtsE | [ftsE-1](http://www.ncbi.nlm.nih.gov/entrez/query.fcgi?cmd=search&db=gene&term=ftsE-1) |  |  |  | 3.222 |  |  |  |  | replication |
| OG1RF_10609 | NrdR family transcriptional regulator |  |  |  |  | 3.205 |  |  |  |  | transcriptonal regulator |
| EF0380 | conserved hypothetical protein |  |  |  |  | 3.177 |  |  |  |  | hypothetical |
| EF1406 | excinuclease ABC subunit C | [uvrC-1](http://www.ncbi.nlm.nih.gov/entrez/query.fcgi?cmd=search&db=gene&term=uvrC-1) |  |  |  | 3.163 |  |  |  |  | DNA repair/recombination |
| EF1934 | conserved hypothetical protein |  |  |  | 4.463 | 3.158 |  |  |  |  | hypothetical |
| EF1605 | conserved hypothetical protein |  |  |  |  | 3.071 |  |  |  |  | hypothetical |
| [OG1RF_10012](http://www.ncbi.nlm.nih.gov/entrez/viewer.fcgi?db=protein&val=327533865) | possible replicative DNA helicase DnaB |  |  |  |  | 3.071 |  |  |  |  | replication |
| EF1404 | MutS family DNA mismatch repair protein |  |  |  | 2.845 | 3.064 |  |  |  |  | DNA repair/recombination |
| EF1254 | ABC superfamily ATP binding cassette transporter, membrane protein |  |  |  |  | 2.983 |  |  |  |  | transport and binding |
| EF1151 | cell division initiation protein DivIVA |  |  |  | 3.446 | 2.978 |  |  |  |  | replication |
| EF1150 | protein of hypothetical function DUF1273 |  |  |  | 3.705 | 2.971 |  |  |  |  | hypothetical |
| EF0737 | amidase |  |  |  |  | 2.961 |  |  |  |  | hypothetical |
| EF2889 | 2-hydroxy-3-oxopropionate reductase | [glxR](http://www.ncbi.nlm.nih.gov/entrez/query.fcgi?cmd=search&db=gene&term=glxR) |  |  | 3.462 | 2.959 |  |  |  |  | energy metabolism |
| EF3003 | lipoprotein |  |  |  |  | 2.868 |  |  |  |  | cell envelope |
| OG1RF_10078 | hypothetical protein |  |  |  |  | 2.863 |  |  |  |  | hypothetical |
| EF2768 | ABC superfamily ATP binding cassette transporter, permease protein |  |  |  |  | 2.825 |  |  |  |  | transport and binding |
| EF1410 | transcriptional regulator |  |  |  |  | 2.809 |  |  |  |  | transcriptional regulator |
| EF0540 | N-acetylmannosamine-6-phosphate 2-epimerase | [nanE](http://www.ncbi.nlm.nih.gov/entrez/query.fcgi?cmd=search&db=gene&term=nanE) |  |  |  | 2.779 |  |  |  |  | cell envelope |
| EF2775 | phosphomethylpyrimidine kinase | [thiD-1](http://www.ncbi.nlm.nih.gov/entrez/query.fcgi?cmd=search&db=gene&term=thiD-1) |  |  |  | 2.778 |  |  |  |  | cofactor biosynthesis |
| EF_B0019 | hypothetical protein |  |  |  | 2.759 | 2.775 |  |  |  |  | hypothetical |
| EF2203 | TetR/AcrR family transcriptional regulator |  |  |  | 5.114 | 2.774 |  |  |  |  | transcriptonal regulator |
| EF1702 | protein of hypothetical function DUF523 |  |  |  | 3.729 | 2.759 |  |  |  |  | hypothetical |
| EF2961 | ribokinase | [rbsK](http://www.ncbi.nlm.nih.gov/entrez/query.fcgi?cmd=search&db=gene&term=rbsK) |  |  | 2.995 | 2.751 |  |  |  |  | energy metabolism |
| EF1341 | ABC superfamily ATP binding cassette transporter, ABC protein |  |  |  | 3.664 | 2.729 |  |  |  |  | transport and binding |
| EF2888 | conserved hypothetical protein |  |  |  | 5.046 | 2.711 |  |  |  |  | hypothetical |
| EF0639 | phosphatidylglycerophosphatase |  |  |  | 4.315 | 2.701 |  |  |  |  | stress |
| OG1RF_10808 | hypothetical protein |  |  |  |  | 2.697 |  |  |  |  | hypothetical |
| EF0857 | conserved hypothetical protein |  |  |  | 4.059 | 2.688 |  |  |  |  | hypothetical |
| EF1276 | bacteriophage gp35 family protein |  |  |  | 2.623 | 2.674 |  |  |  |  | phage-related |
| EF1542 | conserved hypothetical protein |  |  |  | 2.391 | 2.639 |  |  |  |  | hypothetical |
| EF1028 | hydrolase |  |  |  |  | 2.607 |  |  |  |  | hypothetical |
| EF1560 | conserved hypothetical protein |  |  |  | 2.69 | 2.584 |  |  |  |  | hypothetical |
| OG1RF_12430 | hypothetical protein |  |  |  |  | 2.545 |  |  |  |  | hypothetical |
| EF2071 | conserved hypothetical protein |  |  |  | 2.471 | 2.544 |  |  |  |  | hypothetical |
| EF2776 | thiamine-phosphate pyrophosphorylase | [thiE](http://www.ncbi.nlm.nih.gov/entrez/query.fcgi?cmd=search&db=gene&term=thiE) |  |  |  | 2.513 |  |  |  |  | cofactor biosynthesis |
| EF2909 | HesB/YadR/YfhF-family protein |  |  |  | 4.392 | 2.51 |  |  |  |  | hypothetical |
| EF1088 | dithiol-disulfide isomerase |  |  |  |  | 2.41 |  |  |  |  | hypothetical |
| EF2433 | phosphoglycerate mutase |  |  |  |  | 2.404 |  |  |  |  | energy metabolism |
| EF1745 | methyl-accepting chemotaxis family protein |  |  |  | 2.578 | 2.352 |  |  |  |  | hypothetical |
| EF1094 | sortase |  |  |  | 2.792 | 2.339 |  |  |  |  | cell envelope |
| EF0385 | major facilitator family transporter |  |  |  |  | 2.311 |  |  |  |  | transport and binding |
| EF1322 | YuaF like protein |  |  |  |  | 2.249 |  |  |  |  | hypothetical |
| EF0236 | aminoacylase |  |  |  | 0.222 | 0.483 |  |  |  |  | peptidase/protease |
| EF2898 | peptidylprolyl isomerase |  |  |  |  | 0.472 |  |  |  |  | protein folding |
| EF0786 | esterase |  |  |  | 0.445 | 0.471 |  |  |  |  | Central and Intermediate metabolism |
| EF3114 | glycerone kinase |  |  |  | 0.411 | 0.453 |  |  |  |  | Central and Intermediate metabolism |
| EF3117 | possible thiamine diphosphokinase | [thiN](http://www.ncbi.nlm.nih.gov/entrez/query.fcgi?cmd=search&db=gene&term=thiN) |  |  | 0.404 | 0.436 |  |  |  |  | Central and Intermediate metabolism |
| EF1694 | ribosomal protein S16 | [rpsP](http://www.ncbi.nlm.nih.gov/entrez/query.fcgi?cmd=search&db=gene&term=rpsP) |  |  | 0.249 | 0.433 |  |  |  |  | ribosomal |
| EF3022 | DAACS family dicarboxylate/amino acid:cation symporter |  |  |  | 0.312 | 0.429 |  |  |  |  | transport and binding |
| OG1RF_11180 | hypothetical protein |  |  |  |  | 0.414 | 0.342 |  |  |  | hypothetical |
| EF1321 | ABC superfamily ATP binding cassette transporter, membrane protein |  |  |  | 0.32 | 0.407 |  |  |  |  | transport and binding |
| EF0785 | MFS family major facilitator transporter |  |  |  | 0.173 | 0.407 |  |  |  |  | antibiotic resistance |
| EF0648 | nitroreductase |  |  |  |  | 0.399 |  |  |  |  | aa metabolism |
| EF0918 | integral membrane protein |  |  |  | 0.408 | 0.398 |  |  |  |  | cell envelope |
| OG1RF_10624 | queuine tRNA-ribosyltransferase | [tgt](http://www.ncbi.nlm.nih.gov/entrez/query.fcgi?cmd=search&db=gene&term=tgt) |  |  |  | 0.398 |  |  |  |  | translation |
| EF1394 | mosc domain protein |  |  |  |  | 0.393 |  |  |  |  | hypothetical |
| EF0744 | DAACS family dicarboxylate/amino acid:sodium (Na+) symporter |  |  |  | 0.183 | 0.391 |  |  |  |  | transport and binding |
| EF0008 | single-strand binding protein | [ssb-1](http://www.ncbi.nlm.nih.gov/entrez/query.fcgi?cmd=search&db=gene&term=ssb-1) |  |  | 0.249 | 0.388 |  |  |  |  | hypothetical |
| EF2218 | response regulator |  |  |  |  | 0.388 |  |  |  |  | Two component system |
| EF2701 | acetyltransferase |  |  |  |  | 0.385 |  |  |  |  | hypothetical |
| EF1806 | tagatose-6-phosphate kinase (phosphotagatokinase) | [lacC-2](http://www.ncbi.nlm.nih.gov/entrez/query.fcgi?cmd=search&db=gene&term=lacC-2) |  |  |  | 0.377 |  |  |  |  | energy metabolism |
| EF1106 | conserved hypothetical protein |  |  |  |  | 0.371 |  |  |  |  | hypothetical |
| EF3031 | possible protein-N(pi)-phosphohistidine--sugar phosphotransferase |  |  |  | 0.384 | 0.361 |  |  |  |  | energy metabolism |
| EF1275 | ribosome-binding factor A | [rbfA](http://www.ncbi.nlm.nih.gov/entrez/query.fcgi?cmd=search&db=gene&term=rbfA) |  |  |  | 0.361 |  |  |  |  | translation |
| EF1259 | HAD superfamily hydrolase |  |  |  | 0.34 | 0.357 |  |  |  |  | hypothetical |
| EF0379 | conserved hypothetical protein |  |  |  |  | 0.355 |  |  |  |  | hypothetical |
| OG1RF_12410 | CDP-diacylglycerol--glycerol-3-phosphate 3-phosphatidyltransferase | [pgsA](http://www.ncbi.nlm.nih.gov/entrez/query.fcgi?cmd=search&db=gene&term=pgsA) |  |  | 0.349 | 0.353 |  |  |  |  | cell envelope |
| EF2694 | MTA/SAH nucleosidase | [pfs](http://www.ncbi.nlm.nih.gov/entrez/query.fcgi?cmd=search&db=gene&term=pfs) |  |  |  | 0.353 |  |  |  |  | aa metabolism |
| EF1618 | ethanolamine utilization protein EutH | [eutH](http://www.ncbi.nlm.nih.gov/entrez/query.fcgi?cmd=search&db=gene&term=eutH) |  |  |  | 0.344 |  |  |  |  | energy metabolism |
| EF1084 | universal stress protein |  |  |  |  | 0.341 |  |  |  |  | stress |
| EF_B0044 | conserved hypothetical protein |  |  |  |  | 0.339 |  |  |  |  | hypothetical |
| EF3201 | tRNA-dihydrouridine synthase |  |  |  | 0.262 | 0.334 |  |  |  |  | translation |
| EF0294 | dihydrofolate reductase family protein |  |  |  |  | 0.324 |  |  |  |  | cofactor biosynthesis |
| EF2047 | conserved hypothetical protein |  |  |  | 0.226 | 0.321 |  |  |  |  | hypothetical |
| EF0089 | cell wall surface anchor protein |  | 0.403 |  | 0.277 | 0.318 |  |  |  |  | cell envelope |
| EF2444 | acyl-CoA thioester hydrolase |  |  |  | 0.359 | 0.315 |  |  |  |  | cofactor biosynthesis |
| EF2068 | conserved hypothetical protein |  |  |  | 0.385 | 0.314 |  |  |  |  | hypothetical |
| EF2932 | thiol peroxidase |  |  |  | 0.352 | 0.312 |  |  |  |  | oxidative stress |
| EF2659 | integral membrane protein |  |  |  | 0.319 | 0.311 |  |  |  |  | cell envelope |
| EF0860 | APC family amino acid-polyamine-organocation transporter |  |  |  | 0.097 | 0.311 |  |  |  |  | cell envelope |
| EF2757 | YbbM family protein |  |  |  | 0.309 | 0.309 |  |  |  |  | hypothetical |
| EF1103 | APC family amino acid-polyamine-organocation transporter |  |  |  | 0.218 | 0.304 |  |  |  |  | transport and binding |
| EF2708 | integral membrane protein |  |  |  |  | 0.299 |  |  |  |  | cell envelope |
| EF1579 | LexA repressor | [lexA](http://www.ncbi.nlm.nih.gov/entrez/query.fcgi?cmd=search&db=gene&term=lexA) |  |  | 0.3 | 0.297 |  |  |  |  | transcriptional regulator |
| EF2607 | F0F1 ATP synthase subunit epsilon | [atpC](http://www.ncbi.nlm.nih.gov/entrez/query.fcgi?cmd=search&db=gene&term=atpC) |  |  | 0.231 | 0.297 |  |  |  |  | energy metbolism |
| EF2558 | possible H(+)-transporting two-sector ATPase |  |  |  | 0.251 | 0.295 |  |  |  |  | transport and binding |
| EF3258 | secreted protein |  |  |  | 0.212 | 0.292 |  |  |  |  | hypothetical |
| EF0296 | Na+/H+ antiporter | [napA](http://www.ncbi.nlm.nih.gov/entrez/query.fcgi?cmd=search&db=gene&term=napA) |  |  | 0.38 | 0.287 |  |  |  |  | transport and binding |
| EF1243 | 6-phospho-beta-galactosidase | [lacG](http://www.ncbi.nlm.nih.gov/entrez/query.fcgi?cmd=search&db=gene&term=lacG) |  |  |  | 0.284 |  |  |  |  | energy metabolism |
| EF0100 | seryl-tRNA synthetase | [serS1](http://www.ncbi.nlm.nih.gov/entrez/query.fcgi?cmd=search&db=gene&term=serS1) |  |  |  | 0.283 |  |  |  |  | translation |
| EF1609 | conserved hypothetical protein |  |  |  | 0.256 | 0.282 |  |  |  |  | hypothetical |
| EF1343 | hypothetical protein |  |  |  |  | 0.275 |  |  |  |  | hypothetical |
| EF3084 | ferric (Fe+3) ABC superfamily ATP binding cassette transporter, membrane protein |  |  |  | 0.184 | 0.274 |  |  |  |  | transport and binding |
| EF3321 | citrate lyase acyl carrier protein gamma subunit |  |  |  |  | 0.271 |  |  |  |  | cell envelope |
| EF1943 | possible bicyclomycin resistance protein |  |  |  | 0.27 | 0.27 |  |  |  |  | antibiotic resistance |
| OG1RF_12429 | hypothetical protein |  |  |  | 0.3 | 0.265 |  |  |  |  | hypothetical |
| EF0660 | MATE efflux family protein |  |  |  | 0.223 | 0.265 |  |  |  |  | transport and binding |
| EF2965 | PTS system, hypothetical pentitol phosphotransferase enzyme IIB component |  |  |  |  | 0.264 |  |  |  |  | energy metabolism |
| EF3208 | ABC superfamily ATP binding cassette transporter, membrane protein |  | 0.39 |  |  | 0.262 |  |  |  |  | transport and binding |
| EF1693 | RNA-binding protein |  |  |  | 0.303 | 0.259 |  |  |  |  | translation |
| EF2809 | posssible bacteriophage holin |  |  |  |  | 0.259 |  |  |  |  | hypothetical |
| EF2668 | magnesium transporter | [mgtE](http://www.ncbi.nlm.nih.gov/entrez/query.fcgi?cmd=search&db=gene&term=mgtE) |  |  | 0.232 | 0.258 |  |  |  |  | transport and binding |
| EF0921 | SulP family sulfate permease |  |  |  | 0.187 | 0.25 |  |  |  |  | transport and binding |
| EF1078 | MFS family major facilitator transporter |  |  |  | 0.125 | 0.245 |  |  |  |  | transport and binding |
| EF1104 | conserved hypothetical protein |  |  |  |  | 0.239 |  |  |  |  | hypothetical |
| EF2569 | PucB family protein |  |  |  | 0.287 | 0.238 |  |  |  |  | hypothetical |
| EF0664 | C_GCAxxG_C_C family protein |  |  |  | 0.164 | 0.238 |  |  |  |  | hypothetical |
| EF3306 | protein-N(pi)-phosphohistidine--sugar phosphotransferase | [srlE](http://www.ncbi.nlm.nih.gov/entrez/query.fcgi?cmd=search&db=gene&term=srlE) | 0.154 | 0.116 |  | 0.233 |  |  |  |  | energy metabolism |
| EF2357 | DoxX family protein |  |  |  |  | 0.23 |  |  |  |  | hypothetical |
| EF2681 | HAD superfamily hydrolase |  |  |  | 0.197 | 0.227 |  |  |  |  | hypothetical |
| EF0420 | MFS family major facilitator transporter |  |  |  | 0.28 | 0.224 |  |  |  |  | antibiotic resistance? |
| EF2935 | NCS2 family nucleobase:cation symporter-2 |  |  |  | 0.148 | 0.22 |  |  |  |  | transport and binding |
| EF3083 | possible phosphonate-transporting ATPase |  |  |  |  | 0.216 |  |  |  |  | transport and binding |
| EF2615 | conserved hypothetical protein |  |  |  | 0.175 | 0.215 |  |  |  |  | hypothetical |
| EF0055 | metal ABC superfamily ATP binding cassette transporter, binding protein |  |  |  | 0.175 | 0.214 |  |  |  |  | transport and binding |
| EF0720 | hypothetical protein |  |  |  |  | 0.198 |  |  |  |  | hypothetical |
| EF0838 | pyridoxal phosphate-dependent enzyme |  |  |  |  | 0.196 |  |  |  |  | hypothetical |
| EF3133 | conserved hypothetical protein |  |  |  |  | 0.189 |  |  |  |  | hypothetical |
| EF0589 | conserved hypothetical protein |  |  |  | 0.321 | 0.187 |  |  |  |  | hypothetical |
| EF2647 | GntP family gluconate:proton (H+) symporter |  | 0.253 | 0.378 |  | 0.184 |  |  |  |  | transport and binding |
| OG1RF_11928 | possible LIV-E family branched chain amino acid exporter AzlD |  |  |  |  | 0.182 |  |  |  |  | transport and binding |
| EF2562 | probable flavodoxin |  | 0.181 |  |  | 0.178 |  |  |  |  | cofactor biosynthesis |
| EF1657 | membrane protein, toxin regulator |  |  |  | 0.235 | 0.176 |  |  |  |  | cell envelope |
| EF0101 | S33 family lysophophospholipase |  |  |  |  | 0.172 |  |  |  |  | hypothetical |
| EF0476 | ferrous iron transport protein B | [feoB](http://www.ncbi.nlm.nih.gov/entrez/query.fcgi?cmd=search&db=gene&term=feoB) |  |  | 0.061 | 0.16 |  |  |  |  | cofactor biosynthesis |
| EF0462 | conserved hypothetical protein |  |  |  | 0.245 | 0.158 |  |  |  |  | hypothetical |
| EF1221 | spermidine/putrescine ABC superfamily ATP binding cassette transporter, spermidine/putrescine-binding protein |  |  |  |  | 0.157 | 0.161 |  |  |  | transport and binding |
| EF2661 | diacylglycerol kinase |  |  |  | 0.19 | 0.149 |  |  |  |  | cell envelope |
| EF2723 | conserved hypothetical protein |  |  |  |  | 0.146 |  |  |  |  | hypothetical |
| EF0757 | conserved hypothetical protein |  | 0.162 |  |  | 0.142 |  |  |  |  | hypothetical |
| EF0958 | PTS system, IIBC component | [exp5](http://www.ncbi.nlm.nih.gov/entrez/query.fcgi?cmd=search&db=gene&term=exp5) |  |  | 0.242 | 0.136 |  |  |  |  | energy metabolism |
| EF1017 | protein-N(pi)-phosphohistidine--sugar phosphotransferase | [celA-5](http://www.ncbi.nlm.nih.gov/entrez/query.fcgi?cmd=search&db=gene&term=celA-5) |  |  |  | 0.136 |  |  |  |  | energy metbolism |
| EF1176 | cell surface protein precursor |  |  |  |  | 0.13 |  |  |  |  | cell envelope |
| EF0193 | ferrichrome ABC superfamily ATP binding cassette transporter, permease protein | [fhuG](http://www.ncbi.nlm.nih.gov/entrez/query.fcgi?cmd=search&db=gene&term=fhuG) |  |  | 0.106 | 0.128 |  |  |  |  | transport and binding |
| EF0192 | iron (Fe3+) ABC superfamily ATP binding cassette transporter, membrane protein |  |  |  | 0.11 | 0.121 |  |  |  |  | transport and binding |
| EF3085 | ABC superfamily ATP binding cassette transporter, membrane protein |  |  |  | 0.078 | 0.106 |  |  |  |  | transport and binding |
| EF3327 | CitMHS family citrate-magnesium (Mg2+):proton (H+) citrate-calcium (Ca2+): proton (H+) symporter |  | 0.123 |  |  | 0.061 |  |  |  |  | transport and binding |
| EF0095 | lipoprotein |  |  |  |  | 0.06 |  |  |  |  | cell envelope |
| EF2987 | conserved hypothetical protein |  |  |  | 31.748 |  |  |  |  |  | hypothetical |
| EF2211 | conserved hypothetical protein |  |  |  | 20.019 |  |  |  |  |  | hypothetical |
| EF2771 | TraX family protein |  |  |  | 13.851 |  |  |  |  |  | hypothetical |
| EF2049 | ABC superfamily ATP binding cassette transporter, permease protein |  |  |  | 9.612 |  |  |  |  |  | transport and binding |
| EF1308 | Dnak protein | [dnaK](http://www.ncbi.nlm.nih.gov/entrez/query.fcgi?cmd=search&db=gene&term=dnaK) |  |  | 9.338 |  |  |  |  |  | stress |
| EF1665 | TraX family protein |  |  |  | 9.053 |  |  |  |  |  | hypothetical |
| EF2914 | transcription elongation factor GreA | [greA](http://www.ncbi.nlm.nih.gov/entrez/query.fcgi?cmd=search&db=gene&term=greA) |  |  | 6.815 |  |  |  |  |  | transcription |
| EF1307 | heat shock protein GrpE | [grpE](http://www.ncbi.nlm.nih.gov/entrez/query.fcgi?cmd=search&db=gene&term=grpE) |  |  | 6.647 |  |  |  |  |  | stress |
| EF0995 | cell division protein FtsQ | [ftsQ](http://www.ncbi.nlm.nih.gov/entrez/query.fcgi?cmd=search&db=gene&term=ftsQ) |  |  | 5.569 |  |  |  |  |  | replication |
| EF1309 | conserved hypothetical protein |  |  |  | 5.38 |  |  |  |  |  | hypothetical |
| EF2458 | conserved hypothetical protein |  |  |  | 5.107 |  |  |  |  |  | hypothetical |
| EF2150 | methicillin resistance factor FemA |  |  |  | 5.082 |  |  |  |  |  | antibiotic resistance |
| EF0925 | conserved hypothetical protein |  |  |  | 4.914 |  |  |  |  |  | hypothetical |
| EF2891 | glycosyltransferase |  |  |  | 4.566 |  |  |  |  |  | glycosyltransferase |
| EF1285 | major tail protein |  |  |  | 4.562 |  |  |  |  |  | hypothetical |
| EF0031 | membrane protein |  |  |  | 4.41 |  |  |  |  |  | cell envelope |
| EF1282 | conserved hypothetical protein |  |  |  | 4.249 |  |  |  |  |  | hypothetical |
| EF3283 | transcriptional regulator CtsR | [ctsR](http://www.ncbi.nlm.nih.gov/entrez/query.fcgi?cmd=search&db=gene&term=ctsR) |  |  | 4.114 |  |  |  |  |  | transcriptional regulator |
| OG1RF_12298 | conserved hypothetical protein |  |  |  | 4.051 |  |  |  |  |  | hypothetical |
| EF1114 | transcriptional regulator |  |  |  | 4.028 |  |  |  |  |  | transcriptional regulator |
| EF1306 | heat-inducible transcription repressor | [hrcA](http://www.ncbi.nlm.nih.gov/entrez/query.fcgi?cmd=search&db=gene&term=hrcA) |  |  | 4.014 |  |  |  |  |  | stress |
| EF2664 | possible phosphoglycerate mutase | [gpmB](http://www.ncbi.nlm.nih.gov/entrez/query.fcgi?cmd=search&db=gene&term=gpmB) |  |  | 3.888 |  |  |  |  |  | energy metabolism |
| EF2642 | glycine betaine/carnitine/choline ABC superfamily ATP binding cassette transporter, membrane/binding protein |  |  |  | 3.844 |  |  |  |  |  | transport and binding |
| EF0263 | possible tRNA(Ile)-lysidine synthase |  |  |  | 3.778 |  |  |  |  |  | translation |
| EF0991 | penicillin-binding protein C | [pbpC](http://www.ncbi.nlm.nih.gov/entrez/query.fcgi?cmd=search&db=gene&term=pbpC) |  |  | 3.715 |  |  |  |  |  | cell envelope |
| EF3062 | rod shape-determining protein MreC | [mreC](http://www.ncbi.nlm.nih.gov/entrez/query.fcgi?cmd=search&db=gene&term=mreC) |  |  | 3.68 |  |  |  |  |  | cell envelope |
| OG1RF_10690 | hypothetical protein |  |  |  | 3.671 |  |  |  |  |  | hypothetical |
| EF1287 | conserved hypothetical protein |  |  |  | 3.656 |  |  |  |  |  | hypothetical |
| EF1279 | DNA replication protein |  |  |  | 3.634 |  |  |  |  |  | replication |
| EF1286 | conserved hypothetical protein |  |  |  | 3.627 |  |  |  |  |  | hypothetical |
| EF0963 | conserved hypothetical protein |  |  |  | 3.593 |  |  |  |  |  | hypothetical |
| EF2422 | homoserine dehydrogenase | [hom](http://www.ncbi.nlm.nih.gov/entrez/query.fcgi?cmd=search&db=gene&term=hom) |  |  | 3.575 |  |  |  |  |  | aa metabolism |
| EF2591 | extradiol dioxygenase |  |  |  | 3.568 |  |  |  |  |  | Aromatic compound metabolism |
| EF0277 | methylated-DNA--protein-cysteine S-methyltransferase | [ogt](http://www.ncbi.nlm.nih.gov/entrez/query.fcgi?cmd=search&db=gene&term=ogt) |  |  | 3.551 |  |  |  |  |  | DNA repair/recombination |
| OG1RF_10823 | possible ATP-binding nuclease |  |  |  | 3.47 |  |  |  |  |  | hypothetical |
| EF1770 | conserved hypothetical protein |  |  |  | 3.454 |  |  |  |  |  | hypothetical |
| EF0638 | hypothetical lipoprotein |  |  |  | 3.435 |  |  |  |  |  | hypothetical |
| EF3179 | ECF anti-sigma factor |  |  |  | 3.422 |  |  |  |  |  | transcription |
| EF1374 | alkylphosphonate utilization operon protein PhnA | [phnA](http://www.ncbi.nlm.nih.gov/entrez/query.fcgi?cmd=search&db=gene&term=phnA) |  |  | 3.222 |  |  |  |  |  | central and intermediate metabolism |
| EF1730 | transcriptional regulator |  |  |  | 3.201 |  |  |  |  |  | transcriptional regulator |
| EF3049 | RpiR family transcriptional regulator |  |  |  | 3.2 |  |  |  |  |  | transcriptional regulator |
| EF3194 | murein hydrolase regulator LrgA |  | 6.906 |  | 3.142 |  |  |  |  |  | cell envelope |
| EF3086 | YueI-like protein |  |  |  | 3.141 |  |  |  |  |  | hypothetical |
| EF1547 | cytidylate kinase | [cmk](http://www.ncbi.nlm.nih.gov/entrez/query.fcgi?cmd=search&db=gene&term=cmk) |  |  | 3.116 |  |  |  |  |  | purine/pyrimidine mebolism |
| EF1291 | conserved hypothetical protein |  |  |  | 3.048 |  |  |  |  |  | hypothetical |
| EF3164 | methionine sulfoxide reductase B | [msrB](http://www.ncbi.nlm.nih.gov/entrez/query.fcgi?cmd=search&db=gene&term=msrB) |  |  | 3.02 |  |  |  |  |  | aa metabolism |
| EF1789 | flotillin |  | 6.429 |  | 2.957 |  |  |  |  |  | hypothetical |
| OG1RF_11110 | hypothetical protein |  |  |  | 2.883 |  |  |  |  |  | hypothetical |
| EF1211 | NADH peroxidase | [npr](http://www.ncbi.nlm.nih.gov/entrez/query.fcgi?cmd=search&db=gene&term=npr) |  |  | 2.835 |  |  |  |  |  | oxidative stress |
| EF0919 | acetyltransferase |  |  |  | 2.821 |  |  |  |  |  | Central and Intermediate metabolism |
| EF0795 | radical SAM superfamily protein |  |  |  | 2.814 |  |  |  |  |  | hypothetical |
| EF1284 | structural protein |  |  |  | 2.783 |  |  |  |  |  | cell envelope |
| EF2910 | Trk family potassium (K+) transporter, NAD+ binding protein |  |  |  | 2.771 |  |  |  |  |  | transport and binding |
| EF2149 | conserved hypothetical protein |  |  |  | 2.759 |  |  |  |  |  | hypothetical |
| EF0264 | hypoxanthine-guanine phosphoribosyltransferase | [hpt](http://www.ncbi.nlm.nih.gov/entrez/query.fcgi?cmd=search&db=gene&term=hpt) |  |  | 2.733 |  |  |  |  |  | purine/pyrimidine metabolism |
| EF1229 | conserved hypothetical protein |  |  |  | 2.641 |  |  |  |  |  | hypothetical |
| EF2206 | nucleoside deaminase |  |  |  | 2.621 |  |  |  |  |  | purine metabolism |
| EF2373 | conserved hypothetical protein |  |  |  | 2.606 |  |  |  |  |  | hypothetical |
| EF0266 | heat shock protein Hsp33 |  |  |  | 2.598 |  |  |  |  |  | stress |
| EF0947 | HAD superfamily hydrolase |  |  |  | 2.528 |  |  |  |  |  | hypothetical |
| EF1700 | signal recognition particle protein | [ffh](http://www.ncbi.nlm.nih.gov/entrez/query.fcgi?cmd=search&db=gene&term=ffh) |  |  | 2.517 |  |  |  |  |  | cell envelope |
| EF1734 | conserved hypothetical protein |  |  |  | 2.507 |  |  |  |  |  | hypothetical |
| EF0038 | gamma-glutamyl kinase | [proB](http://www.ncbi.nlm.nih.gov/entrez/query.fcgi?cmd=search&db=gene&term=proB) |  |  | 2.46 |  |  |  |  |  | aa metabolism |
| EF2744 | possible glutamyl aminopeptidase | [celM](http://www.ncbi.nlm.nih.gov/entrez/query.fcgi?cmd=search&db=gene&term=celM) |  |  | 2.445 |  |  |  |  |  | aa metabolism |
| EF0935 | topoisomerase-primase (TOPRIM) domain protein |  |  |  | 2.405 |  |  |  |  |  | hypothetical |
| EF2739 | peroxiredoxin | [ahpC](http://www.ncbi.nlm.nih.gov/entrez/query.fcgi?cmd=search&db=gene&term=ahpC) |  |  | 2.391 |  |  |  |  |  | oxidative stress |
| EF0377 | ankyrin repeat-containing protein |  |  |  | 2.33 |  |  |  |  |  | hypothetical |
| EF1137 | conserved hypothetical protein |  |  |  | 2.318 |  |  |  |  |  | hypothetical |
| EF1133 | 2,3,4,5-tetrahydropyridine-2-carboxylate N-succinyltransferase | [dapD](http://www.ncbi.nlm.nih.gov/entrez/query.fcgi?cmd=search&db=gene&term=dapD) |  |  | 2.317 |  |  |  |  |  | purine/pyrimidine metabolism |
| EF3002 | MarR family transcriptional regulator |  |  |  | 2.279 |  |  |  |  |  | transcriptional regulator |
| EF1305 | hypothetical protein |  | 3.607 |  | 2.186 |  |  |  |  |  | hypothetical |
| EF1249 | fibronectin binding protein A |  |  |  | 2.125 |  |  |  |  |  | cell envelope |
| EF0768 | protein of hypothetical function DUF199 |  |  |  | 2.107 |  |  |  |  |  | hypothetical |
| EF2353 | GNAT family acetyltransferase |  |  |  | 0.511 |  |  |  |  |  | hypothetical |
| EF0202 | phosphomethylpyrimidine kinase | [thiD-2](http://www.ncbi.nlm.nih.gov/entrez/query.fcgi?cmd=search&db=gene&term=thiD-2) |  |  | 0.493 |  |  |  |  |  | purine/pyrimidine metabolism |
| EF1366 | integral membrane protein |  |  |  | 0.471 |  |  |  |  |  | cell envelope |
| EF0067 | Holliday junction DNA helicase B | [ruvB](http://www.ncbi.nlm.nih.gov/entrez/query.fcgi?cmd=search&db=gene&term=ruvB) |  |  | 0.458 |  |  |  |  |  | replication |
| OG1RF_11645 | possible DNA methyltransferase |  |  |  | 0.441 |  |  |  |  |  | hypothetical |
| EF0083 | conserved hypothetical protein |  |  |  | 0.428 |  |  |  |  |  | hypothetical |
| EF0449 | possible 2-succinyl-6-hydroxy-2,4-cyclohexadiene-1-carboxylate synthase | [menH](http://www.ncbi.nlm.nih.gov/entrez/query.fcgi?cmd=search&db=gene&term=menH) |  |  | 0.424 |  |  |  |  |  | Central and Intermediate metabolism |
| EF0077 | conserved hypothetical protein |  |  |  | 0.411 |  |  |  |  |  | hypothetical |
| EF1522 | RNA polymerase sigma factor RpoD | [sigA](http://www.ncbi.nlm.nih.gov/entrez/query.fcgi?cmd=search&db=gene&term=sigA) |  |  | 0.409 |  |  |  |  |  | transcription |
| EF0076 | possible glucose 1-dehydrogenase |  |  |  | 0.408 |  |  |  |  |  | energy metabolism |
| EF0115 | endoribonuclease L-PSP |  |  |  | 0.403 |  |  |  |  |  | translation |
| EF2199 | ribonuclease BN |  |  |  | 0.402 |  |  |  |  |  | RNAse |
| EF2778 | ThiW family protein |  |  |  | 0.393 |  |  |  |  |  | hypothetical |
| EF0463 | Mn superoxide dismutase | [sodA](http://www.ncbi.nlm.nih.gov/entrez/query.fcgi?cmd=search&db=gene&term=sodA) |  |  | 0.388 |  |  |  |  |  | oxidative stress |
| EF1717 | carbamoyl phosphate synthase small subunit | [pyraA](http://www.ncbi.nlm.nih.gov/entrez/query.fcgi?cmd=search&db=gene&term=pyraA) |  | 0.434 | 0.383 |  |  |  |  |  | aa metabolism |
| EF0576 | ABC superfamily ATP binding cassette transporter, membrane protein |  |  |  | 0.378 |  |  |  |  |  | transport and binding |
| EF0448 | 2-succinyl-6-hydroxy-2, 4-cyclohexadiene-1-carboxylic acid synthase/2-oxoglutarate decarboxylase | [menD](http://www.ncbi.nlm.nih.gov/entrez/query.fcgi?cmd=search&db=gene&term=menD) |  |  | 0.377 |  |  |  |  |  | Central and Intermediate metabolism |
| EF1898 | 50S ribosomal protein L19 | [rplS](http://www.ncbi.nlm.nih.gov/entrez/query.fcgi?cmd=search&db=gene&term=rplS) |  |  | 0.373 |  |  |  |  |  | ribosomal |
| EF3172 | competence/damage-inducible protein CinA | [cinA](http://www.ncbi.nlm.nih.gov/entrez/query.fcgi?cmd=search&db=gene&term=cinA) |  |  | 0.373 |  |  |  |  |  | DNA repair/recombination |
| EF1147 | CTP synthetase | [pyrG](http://www.ncbi.nlm.nih.gov/entrez/query.fcgi?cmd=search&db=gene&term=pyrG) | 0.45 |  | 0.369 |  |  |  |  |  | purine/pyrimidin mebolism |
| EF1516 | N-acetylglucosamine PTS, EIICBA | [ptsG](http://www.ncbi.nlm.nih.gov/entrez/query.fcgi?cmd=search&db=gene&term=ptsG) |  |  | 0.359 |  |  |  |  |  | energy metabolism |
| EF1042 | MFS family major facilitator transporter, multidrug:cation symporter |  |  |  | 0.358 |  |  |  |  |  | transport and binding |
| EF3142 | phosphogluconate dehydrogenase (decarboxylating) |  |  |  | 0.357 |  |  |  |  |  | energy metabolism |
| EF3053 | PTT family thiamin transporter |  |  |  | 0.354 |  |  |  |  |  | transport and binding |
| EF0982 | hemolysin A | [tlyA](http://www.ncbi.nlm.nih.gov/entrez/query.fcgi?cmd=search&db=gene&term=tlyA) |  |  | 0.353 |  |  |  |  |  | virulence factors |
| EF2149B | conserved hypothetical protein |  |  |  | 0.353 |  |  |  |  |  | hypothetical |
| EF2742 | metal-dependent hydrolase |  |  |  | 0.353 |  |  |  |  |  | peptidase/protease |
| EF0717 | PTS system, fructose specific IIABC component | [fruA](http://www.ncbi.nlm.nih.gov/entrez/query.fcgi?cmd=search&db=gene&term=fruA) |  |  | 0.35 |  |  |  |  |  | energy metabolism |
| EF1780 | phosphoribosylformylglycinamidine cyclo-ligase | [purM](http://www.ncbi.nlm.nih.gov/entrez/query.fcgi?cmd=search&db=gene&term=purM) |  |  | 0.344 |  |  |  |  |  | purine/pyrimidine mebolism |
| EF0941 | possible beta-glucan-transporting ATPase |  |  |  | 0.338 |  |  |  |  |  | transport and binding |
| EF2868 | SAM-dependent methyltransferase |  |  |  | 0.337 |  |  |  |  |  | cofacror tmetabolism |
| EF0543 | multitransmembrane protein |  |  |  | 0.334 |  |  |  |  |  | cell envelope |
| EF1240 | LacI family sugar-binding transcriptional regulator |  |  |  | 0.331 |  |  |  |  |  | transcriptional regulator |
| EF2683 | WxL domain surface protein |  |  |  | 0.33 |  |  |  |  |  | cell envelope |
| EF0492 | conserved hypothetical protein |  |  |  | 0.327 |  |  |  |  |  | hypothetical |
| EF1349 | oligo-1,6-glucosidase | [malL-2](http://www.ncbi.nlm.nih.gov/entrez/query.fcgi?cmd=search&db=gene&term=malL-2) |  |  | 0.327 |  |  |  |  |  | energy metabolism |
| EF0172 | transcriptional regulator |  |  |  | 0.325 |  |  |  |  |  | transcriptional regulator |
| EF0179 | ABC superfamily, ATP binding cassette transporter, membrane protein |  |  |  | 0.315 |  |  |  |  |  | transport and binding |
| EF1070 | possible UDP-glucose 4-epimerase |  |  |  | 0.312 |  |  |  |  |  | energy metabolism |
| OG1RF_11539 | possible membrane protein |  |  |  | 0.312 |  |  |  |  |  | cell envelope |
| EF0942 | ABC superfamily ATP binding cassette transporter, membrane protein |  |  |  | 0.31 |  |  |  |  |  | transport and binding |
| EF1901 | NRAMP family manganese (Mn2+) transporter |  |  |  | 0.308 |  |  |  |  |  | transport and binding |
| EF3066 | peptide deformylase | [def-2](http://www.ncbi.nlm.nih.gov/entrez/query.fcgi?cmd=search&db=gene&term=def-2) |  |  | 0.298 |  |  |  |  |  | peptidase/protease |
| EF3013 | Mga family transcriptional regulator |  |  |  | 0.296 |  |  |  |  |  | transcriptional regulator |
| EF0556 | xylose isomerase | [xylA](http://www.ncbi.nlm.nih.gov/entrez/query.fcgi?cmd=search&db=gene&term=xylA) | 0.253 | 0.247 | 0.289 |  |  |  |  |  | energy metabolism |
| EF0922 | integral membrane protein |  |  |  | 0.287 |  |  |  |  |  | cell envelope |
| EF3281 | conserved hypothetical protein |  |  |  | 0.286 |  |  |  |  |  | hypothetical |
| EF0643 | conserved hypothetical protein |  | 0.295 |  | 0.281 |  |  |  |  |  | hypothetical |
| EF1704 | sensor histidine kinase |  |  |  | 0.281 |  |  |  |  |  | Two component system |
| EF1365 | permease |  |  |  | 0.28 |  |  |  |  |  | transport and binding |
| EF3055 | conserved hypothetical protein |  |  |  | 0.28 |  |  |  |  |  | hypothetical |
| EF1139 | possible gamma-glutamyl-gamma-aminobutyrate hydrolase | puuD | 0.18 | 0.419 | 0.276 |  |  |  |  |  | Central and Intermediate metabolism |
| EF1918 | carboxy-cis,cis-muconate cyclase |  |  |  | 0.276 |  |  |  |  |  | Aromatic compound metabolism |
| EF1004 | glucose-6-phosphate 1-dehydrogenase | [zwf](http://www.ncbi.nlm.nih.gov/entrez/query.fcgi?cmd=search&db=gene&term=zwf) |  |  | 0.265 |  |  |  |  |  | energy metabolism |
| EF1529 | PTS family porter component IIC |  |  |  | 0.242 |  |  |  |  |  | energy metabolism |
| EF3323 | conserved hypothetical protein |  |  |  | 0.242 |  |  |  |  |  | hypothetical |
| EF1241 | conserved hypothetical protein |  |  |  | 0.238 |  |  |  |  |  | hypothetical |
| EF1198 | conserved hypothetical protein |  |  |  | 0.236 |  |  |  |  |  | hypothetical |
| EF1916 | GTPase |  |  |  | 0.228 |  |  |  |  |  | central and intermediate metabolism |
| EF0107 | Crp family transcriptional regulator |  |  |  | 0.218 |  |  |  |  |  | transcriptional regulator |
| EF1200 | membrane protein |  |  |  | 0.215 |  |  |  |  |  | cell envelope |
| EF1117 | glutamine ABC superfamily ATP binding cassette transporter, membrane protein |  |  |  | 0.198 |  |  |  |  |  | transport and binding |
| EF0905 | pentapeptide repeat-containing protein |  |  |  | 0.195 |  |  |  |  |  | hypothetical |
| EF2657 | membrane protein |  |  |  | 0.173 |  |  |  |  |  | cell envelope |
| EF0805 | ABC superfamily ATP binding cassette transporter, ABC protein |  |  | 0.198 | 0.171 |  |  |  |  |  | transport and binding |
| EF2060 | cytochrome d ubiquinol oxidase, subunit II | [cydB](http://www.ncbi.nlm.nih.gov/entrez/query.fcgi?cmd=search&db=gene&term=cydB) |  |  | 0.157 |  |  |  |  |  | oxidative stress |
| EF2573 | NCS2 family nucleobase:cation symporter-2 |  |  |  | 0.143 |  |  |  |  |  | transport and binding |
| EF3285 | PTS family oligomeric beta-glucoside porter component IIC |  |  |  | 0.14 |  |  |  |  |  | energy metabolism |
| EF0288 | conserved hypothetical protein |  |  |  | 0.125 |  |  |  |  |  | hypothetical |
| EF0208 | ribosomal protein L23 | [rplW](http://www.ncbi.nlm.nih.gov/entrez/query.fcgi?cmd=search&db=gene&term=rplW) |  | 0.454 |  |  |  |  |  |  | riboosomal protein |
| EF0423 | keto-hydroxyglutarate-aldolase/keto-deoxy- phosphogluconate aldolase | [eda-1](http://www.ncbi.nlm.nih.gov/entrez/query.fcgi?cmd=search&db=gene&term=eda-1) | 0.347 |  |  |  |  |  |  |  | Central and Intermediate metabolism |
| EF0437 | AraC family transcriptional regulator |  | 0.453 |  |  |  |  |  |  |  | transcriptional regulator |
| EF0467 | MgtC family magnesium (Mg2+) transporter-C |  |  | 0.442 |  |  |  |  |  |  | transport and binding |
| EF0539 | transcriptional regulator RpiR |  |  | 0.249 |  |  |  |  |  |  | transcriptional regulator |
| EF0706 | prophage Lp2 protein 7 |  | 0.298 |  |  |  |  |  |  |  | prophage |
| EF0720B | hypothetical protein |  | 0.198 |  |  |  |  |  |  |  | transport and binding |
| EF0781 | cold-shock protein |  | 2.468 |  |  |  |  |  |  |  | stress |
| EF0812 | family 88 glycosyl hydrolase |  |  |  |  |  | 0.229 |  |  |  | hypothetical |
| EF0833 | membrane protein |  | 0.455 | 0.176 |  |  |  |  |  |  | cell envelope |
| EF0854 | signal peptidase I |  | 0.385 |  |  |  |  |  |  |  | peptidase/protease |
| EF1020 | possible beta-glucosidase |  |  | 0.351 |  |  |  |  |  |  | energy metabolism |
| EF1066 | maltose O-acetyltransferase | [maa](http://www.ncbi.nlm.nih.gov/entrez/query.fcgi?cmd=search&db=gene&term=maa) | 0.333 |  |  |  |  |  |  |  | energy metabolism |
| EF1207 | CCS family citrate:cation symporter |  |  |  |  |  | 0.324 |  |  |  | transport and binding |
| EF1236 | cephalosporin-C deacetylase |  |  | 0.411 |  |  |  |  |  |  | antibiotic resistance |
| EF1345 | sugar ABC superfamily ATP binding cassette transporter, sugar-binding protein |  | 0.299 | 0.237 |  |  |  |  |  |  | transport and binding |
| EF1397 | molybdenum ABC superfamily ATP binding cassette transporter, binding protein |  |  | 2.294 |  |  |  |  |  |  | transport and binding |
| EF1551 | hypothetical protein |  | 0.414 |  |  |  |  |  |  |  | hypothetical |
| EF1599B | transcriptional regulator |  | 5.16 |  |  |  |  |  |  |  | transcriptional regulator |
| EF1617 | ethanolamine utilization protein EutQ |  |  |  |  |  | 0.384 |  |  |  | energy metabolism |
| EF1746 | UTP-glucose-1-phosphate uridylyltransferase | [galU](http://www.ncbi.nlm.nih.gov/entrez/query.fcgi?cmd=search&db=gene&term=galU) | 2.211 |  |  |  |  |  |  |  | purine/pyrimidine mebolism |
| EF1915 | conserved hypothetical protein |  | 3.404 |  |  |  |  |  |  |  | hypothetical |
| EF1943B | conserved hypothetical protein |  | 0.262 |  |  |  |  |  |  |  | hypothetical |
| EF2074 | possible phosphonate-transporting ATPase |  | 0.148 |  |  |  |  |  |  |  | transport and binding |
| EF2199 | hypothetical protein |  | 2.437 |  |  |  |  |  |  |  | hypothetical |
| EF2580 | dihydropyrimidinase |  |  | 0.213 |  |  |  |  |  |  | purine/pyrimidine metabolism |
| EF2687 | conserved hypothetical protein |  |  | 0.468 |  |  |  |  |  |  | hypothetical |
| EF2794 | membrane protein |  |  | 0.337 |  |  |  |  |  |  | cell envelope |
| EF2967 | conserved hypothetical protein |  |  | 0.43 |  |  |  |  |  |  | hypothetical |
| EF3240 | possible biotin--[acetyl-CoA-carboxylase] ligase | [birA](http://www.ncbi.nlm.nih.gov/entrez/query.fcgi?cmd=search&db=gene&term=birA) | 0.392 |  |  |  |  |  |  |  | cofactor biosynthesis |
| [OG1RF_10601](http://www.ncbi.nlm.nih.gov/entrez/viewer.fcgi?db=protein&val=327534454) | KUP family potassium (K+) uptake permease |  | 0.196 |  |  |  |  |  |  |  | transport and binding |
| OG1RF_10810 | possible Cna family adhesin |  | 3.051 |  |  |  |  |  |  |  | cell envelope |
