# Supplementary material for: The Cell Wall-Targeting Antibiotic Stimulon of Enterococcus faecalis
Source: PLoS One. 2013 Jun 3;8(6):e64875. doi: 10.1371/journal.pone.0064875 (PMC3670847; doi:10.1371/journal.pone.0064875)
Supplement: Table S2 — Validation of microarrays by qRT-PCR. Data is presented in fold of change relative to control conditions measured by qRT-PCR, and by microarrays in parenthesis. (DOCX) [file pone.0064875.s005.docx]

**Table S2** – Validation of microarrays by qRT-PCR. Data is presented in fold of change relative to control conditions measured by qRT-PCR, and by microarrays in parenthesis.

|  | **Treatment** | | | | | | | |
| --- | --- | --- | --- | --- | --- | --- | --- | --- |
| **Gene Locus** | **Amp 30'** | **Amp 60'** | **Bac 30'** | **Bac 60'** | **Cep 30'** | **Cep 60'** | **Van 30'** | **Van**  **60'** |
| EF3245 | 100.19 (4.29) |  | 167.45 (43.91) |  |  | 83.59 (34.14) |  | 561.51 (112.67) |
| EF0797 |  | 11.38 (8.13) | 58.11 (10.19) |  |  | 17.53 (11.30) |  | 11.32 (10.86) |
| EF1533 |  | 26.06 (11.77) | 514.10 (62.15) |  |  | 107.00 (38.96) | 4.16 (13.64) |  |
| EF0026 |  | 7.31 (9.07) | 18.21 (19.06) |  |  | 4.64 (12.18) |  | 1.44 (11.90) |
| EF1753 | 2.56  (4.41) |  | 63.98 (25.32) |  |  | 2.56 (10.20) |  | 16.44 (18.17) |
| EF0708 | 10.75 (3.66) |  | 10.02 (15.87) |  |  | 6.94 (9.02) |  | 37.73 (16.40) |
| EF3152 | 0.91  (NC) |  | 0.19 (0.34) |  | 0.19 (0.51) |  | 0.13 (0.20) |  |
| EF1258 |  | 4.41 (3.80) |  | 0.14 (11.19) |  | 0.96 (8.48) | 4.30 (23.70) |  |
| EF2554 | 3.55  (NC) |  |  | 0.08 (0.276) |  | 1.04 (0.318) | 1.57 (0.48) |  |
|  |  |  |  |  |  |  |  |  |
| NC=No change | |  |  |  |  |  |  |  |
