# Supplementary material for: The Cell Wall-Targeting Antibiotic Stimulon of Enterococcus faecalis
Source: PLoS One. 2013 Jun 3;8(6):e64875. doi: 10.1371/journal.pone.0064875 (PMC3670847; doi:10.1371/journal.pone.0064875)
Supplement: Table S3 — Quantitative RT-PCR primers used to validate microarray data. (DOCX) [file pone.0064875.s006.docx]

**Table S3 –** Quantitative RT-PCR primers used to validate microarray data**.**

| **Gene** | **Direction** | **Primer** | **Sequence** |
| --- | --- | --- | --- |
| EF3245 | Forward | pm017 | ATGTCATCGCTGGGATGCTCCTAA |
|  | Reverse | pm018 | CAGGTGCCGGCAAATAACGTTGAA |
| EF0708 | Forward | pm019 | GCTGGAGAAATCTCTGAATTAATCGTGG |
|  | Reverse | pm020 | GCGCGGTCAGGTAAAGAAATCCAA |
| EF0797 | Forward | pm021 | GCTCGTGGAAACATCCAAGCAACA |
|  | Reverse | pm022 | TCTCCATGGACAAGAGCGACAGTT |
| EF1753 | Forward | pm023 | TGGAAAGATCAAGCCACAGACACC |
|  | Reverse | pm024 | AAGAATTCTCCCACTTGACCGCCA |
| EF1533 | Forward | pm025 | ACGCAGAGAAGAACTCTGGCGAAT |
|  | Reverse | pm026 | GCGGCTACTTGCGTATAACTAGCA |
| EF1258 | Forward | pm027 | GGTTACTCTACGGAATTAGCAGTGTCG |
|  | Reverse | pm028 | TGCACAGAGAGCTACTCCCACTACTT |
| EF0026 | Forward | pm029 | TGTCGGTTCACTCTTTAGTGCCGT |
|  | Reverse | pm030 | ACAAGCCACACGATAATCGCTACG |
| EF3152 | Forward | pm031 | ACGGCCTTCGTCCTCTTCTTGATT |
|  | Reverse | pm032 | ACCACTTCTTCTTCTGCCGCTTCT |
| EF2554 | Forward | pm033 | AAGTGACCCAGCAGTCATTAGCGA |
|  | Reverse | pm034 | ATCGGCGATAAACCTCAACCGTCT |
